# Supplementary material for: Adolescent mental health difficulties and educational attainment: findings from the UK household longitudinal study
Source: BMJ Open. 2021 Jul 25;11(7):e046792. doi: 10.1136/bmjopen-2020-046792 (PMC8372813; doi:10.1136/bmjopen-2020-046792)
Supplement: Supplementary data [file bmjopen-2020-046792supp001.pdf]

Supplementary table A: Odds ratios for low attainment at Key Stage 4 by total mental health difficulties and domain scores, adjusted stepwise for explanatory factors, by sex.

	Emotional		Peer		Conduct		Hyperactivity		Total score	
	Males	Females	Males	Females	Males	Females	Males	Females	Males	Females
Model 1	**3.07** [1.48,6.38]**	1.49 [0.91,2.43]	**2.36** [1.39,4.02]**	**2.55** [1.45,4.48]**	**1.65* [1.03,2.66]**	**2.17** [1.22,3.86]**	**2.35*** [1.49,3.71]**	**2.63*** [1.59,4.35]**	**3.16*** [1.79,5.60]**	**3.36*** [1.97,5.71]**
Model 2	**3.22** [1.55,6.71]**	1.54 [0.95,2.50]	**2.43** [1.43,4.14]**	**2.78*** [1.59,4.86]**	**1.71* [1.06,2.76]**	**2.15* [1.18,3.89]**	**2.40*** [1.51,3.79]**	**2.61*** [1.56,4.37]**	**3.30*** [1.85,5.87]**	**3.47*** [2.03,5.92]**
Model 3	**2.91* [1.17,7.22]**	1.41 [0.77,2.57]	**2.34* [1.20,4.56]**	1.31 [0.61,2.79]	1.56 [0.86,2.85]	**2.67** [1.35,5.26]**	**2.40** [1.36,4.24]**	**3.45*** [1.90,6.27]**	**3.39*** [1.74,6.62]**	**3.91*** [2.04,7.51]**
Model 4	**2.89* [1.05,7.92]**	1.23 [0.63,2.42]	**2.23* [1.07,4.63]**	1.26 [0.55,2.90]	1.44 [0.76,2.72]	**2.42* [1.16,5.05]**	**2.68** [1.43,5.04]**	**3.43*** [1.75,6.73]**	**3.38** [1.64,6.98]**	**3.52*** [1.69,7.32]**
Model 5	2.37 [0.85,6.59]	0.98 [0.49,1.97]	1.90 [0.93,3.87]	1.12 [0.48,2.60]	1.03 [0.50,2.12]	1.82 [0.85,3.93]	**2.23* [1.15,4.31]**	**2.64** [1.35,5.18]**	**2.66* [1.25,5.70]**	**2.61* [1.22,5.57]**
Model 6	2.51 [0.87,7.28]	0.76 [0.35,1.65]	1.85 [0.88,3.90]	1.09 [0.45,2.64]	0.98 [0.45,2.14]	1.35 [0.54,3.32]	**2.17* [1.13,4.19]**	**2.73* [1.24,6.03]**	**2.86** [1.30,6.29]**	1.79 [0.76,4.25]
Model 7	2.36 [0.83,6.64]	0.73 [0.34,1.57]	1.79 [0.83,3.84]	0.99 [0.41,2.40]	0.93 [0.42,2.05]	1.29 [0.52,3.18]	**2.17* [1.11,4.23]**	**2.85** [1.30,6.23]**	**2.77* [1.24,6.16]**	1.69 [0.72,3.95]

Note: Imputed model, Males N=550; Females N=560
Model 1: unadjusted odds of socioemotional difficulties; Model 2: adjusts for Model 1 + age, ethnicity; Model 3: adjusts for Model 2 + prior attainment at KS2; Model 4: adjusts for Model 3 + household social class, maternal education, household poverty, family composition Model 5: adjusts for Model 4 + happy with school work, happy with school; Model 6: adjusts Model 5 + parental interest in school, parents attend parent evening, family support, quarrels with parents; Model 7: adjusts for Model 6 +parental mental and physical health.
Significant odds ratios (95% confidence interval) shown in bold text.
