# Supplementary material for: Adolescent mental health difficulties and educational attainment: findings from the UK household longitudinal study
Source: BMJ Open. 2021 Jul 25;11(7):e046792. doi: 10.1136/bmjopen-2020-046792 (PMC8372813; doi:10.1136/bmjopen-2020-046792)
Supplement: Author's manuscript [file bmjopen-2020-046792.draft_revisions.pdf]

BMJ Open

BMJ Open is committed to open peer review. As part of this commitment we make the peer review history of every article we publish publicly available.

When an article is published we post the peer reviewers' comments and the authors' responses online. We also post the versions of the paper that were used during peer review. These are the versions that the peer review comments apply to.

The versions of the paper that follow are the versions that were submitted during the peer review process. They are not the versions of record or the final published versions. They should not be cited or distributed as the published version of this manuscript.

BMJ Open is an open access journal and the full, final, typeset and author-corrected version of record of the manuscript is available on our site with no access controls, subscription charges or pay-per-view fees (http://bmjopen.bmj.com).

If you have any questions on BMJ Open's open peer review process please email info.bmjopen@bmj.com

Adolescent mental health difficulties and educational attainment: findings from the UK Household Longitudinal Study

Journal:	*BMJ Open*
Manuscript ID	bmjopen-2020-046792
Article Type:	Original research
Date Submitted by the Author:	11-Nov-2020

Complete List of Authors:	SMITH, NEIL; NatCen Social Research, Policy Research Centre<br>Marshall, Lydia; NatCen Social Research, Policy Research Centre<br>Albakri, Muslihah; NatCen Social Research, Policy Research Centre<br>Smuk, Melanie; London School of Hygiene & Tropical Medicine<br>Hagell, Ann; Association for Young People's Health<br>Stansfeld, Stephen; Barts and the London, Queen Marys School of Medicine and Dentistry, Psychiatry

Keywords:	MENTAL HEALTH, PUBLIC HEALTH, Child & adolescent psychiatry < PSYCHIATRY, EPIDEMIOLOGY

SCHOLARONE™
Manuscripts

I, the Submitting Author has the right to grant and does grant on behalf of all authors of the Work (as defined in the below author licence), an exclusive licence and/or a non-exclusive licence for contributions from authors who are: i) UK Crown employees; ii) where BMJ has agreed a CC-BY licence shall apply, and/or iii) in accordance with the terms applicable for US Federal Government officers or employees acting as part of their official duties; on a worldwide, perpetual, irrevocable, royalty-free basis to BMJ Publishing Group Ltd ("BMJ") its licensees and where the relevant Journal is co-owned by BMJ to the co-owners of the Journal, to publish the Work in this journal and any other BMJ products and to exploit all rights, as set out in our licence.

The Submitting Author accepts and understands that any supply made under these terms is made by BMJ to the Submitting Author unless you are acting as an employee on behalf of your employer or a postgraduate student of an affiliated institution which is paying any applicable article publishing charge ("APC") for Open Access articles. Where the Submitting Author wishes to make the Work available on an Open Access basis (and intends to pay the relevant APC), the terms of reuse of such Open Access shall be governed by a Creative Commons licence – details of these licences and which Creative Commons licence will apply to this Work are set out in our licence referred to above.

Other than as permitted in any relevant BMJ Author's Self Archiving Policies, I confirm this Work has not been accepted for publication elsewhere, is not being considered for publication elsewhere and does not duplicate material already published. I confirm all authors consent to publication of this Work and authorise the granting of this licence.

For peer review only

Adolescent mental health difficulties and educational attainment: findings from the UK Household Longitudinal Study

Corresponding author:
Neil R Smith, PhD
NatCen Social Research
Policy Research Centre
35 Northampton Square
London
EC1V 0AX, UK
Neil.Smith@natcen.ac.uk

Lydia Marshall, PhD
NatCen Social Research
Policy Research Centre
35 Northampton Square
London
EC1V 0AX, UK
Lydia.Marshall@natcen.ac.uk

Muslihah Albakri
NatCen Social Research
Policy Research Centre
35 Northampton Square
London
EC1V 0AX, UK
Muslihah.Albakri@natcen.ac.uk

Melanie Smuk, PhD
London School of Hygiene & Tropical Medicine
Keppel Street
London
WC1E 7HT, UK
Melanie.Smuk@lshtm.ac.uk

Ann Hagell, PhD
Association for Young People's Health
7-14 Great Dover Street
London
SE1 4YR, UK
Ann@youngpeopleshealth.org.uk

Stephen Stansfeld, PhD
Centre for Psychiatry
Bart's and the London School of Medicine and Dentistry
London
EC1M 6BQ, UK
S.A.Stansfeld@qmul.ac.uk

2,859 words
Keywords: mental health, adolescence, education, social epidemiology, life course

For peer review only

ABSTRACT

Objective: This study examines whether there is an independent association between mental health in adolescence and educational attainment at age 16, after accounting for range of risk factors which might explain poor mental health and lower levels of educational attainment.

Design: Longitudinal study.

Setting: Nationally representative data from the UK Household Longitudinal Study (UKHLS) were linked to the National Pupil Database for England.

Participants: Respondents (N=1,100) to the UKHLS in 2009-2012 were linked to the National Pupil Database to investigate longitudinal associations between mental health at ages 11-14 and educational attainment at age 16 (GCSE).

Primary outcome measure: Not gaining five or more GCSE qualifications at age 16, including English and maths.

Results: Poor mental health measured using the Strengths and Difficulties Questionnaire at ages 11-14 predicted low levels of educational attainment at age 16 (OR: 3.11 (95% CI: [2.11, 4.57]). This association was maintained after controlling for prior attainment, individual demographic and household social and economic factors (3.20, 95% CI [1.90,5.37]. Controlling for parental engagement with school, parent-child relationships and happiness with school(/work) partially attenuated the association which was significant in the fully adjusted model (2.05, 95% CI: [1.15,3.68]). The association was maintained in the fully adjusted model for males only but not for females.

Conclusion: Mental health at ages 11-14 was independently linked to educational success at age 16, highlighting an important pathway through which health in adolescence can determine young people's life chances.

For peer review only

STRENGTHS AND LIMITATIONS OF THIS STUDY.

- This is a large, nationally representative longitudinal cohort study containing self-assessed measures of mental health among young people linked to a National Pupil Database of educational records.
- The study captures a diverse range of social, demographic, economic and behavioural factors affecting young people in their home and school environment, permitting statistical adjustment for multiple confounding relationships which might explain the association between mental health and educational attainment.
- Consent to data linkage between the longitudinal study and the National Pupil Database was incomplete, though factors which predicted patterns of non-consent were controlled for within our models.
- Missing data was accounted for using multiple imputation methods which exploited the wide range of associations within the observed data to minimise errors within estimates of effect.

For peer review only

INTRODUCTION

Growing evidence of the prevalence of poor child and adolescent mental health has led to this issue becoming a key policy priority in the UK. The mental health of children and young people in England declines with age with around 14.4% of 11-16 years experiencing a mental disorder compared to 5.5% in their pre-school counterparts aged 2-4 years.(1) With 75% of adult mental health problems (excluding dementia) starting by the age of 18,(2) adolescence is a key period in the development of long-lasting mental health difficulties. The *Future in Mind* report presented an important economic case for investment in early prevention of mental ill health to mitigate against the costs of longer-term support for health needs. However, this argument neglects the impact that early life mental health potentially has on other early life outcomes fundamental in determining life chances, such as educational attainment.(3) Educational outcomes are closely associated with later-life chances with well-established links to employment, income, housing and offending as well as physical health and on-going mental health disorders. If poor mental health diminishes the capacity for individuals to fulfil their academic potential, mental health itself is likely to be a driver of educational inequality and consequent on-going social inequality.

There are a number of mechanisms through which poor mental health might be expected to lead to lower educational attainment, including for example absence from school (1,4) or poor classroom behaviour.(5,6) On the other hand, the association between mental health and educational outcomes might not be direct, but rather incorporate the influence of confounding factors. A range of demographic and socioeconomic factors, such as gender, ethnicity, socioeconomic disadvantage and maternal education and parental health (7–10) have well-established relationships with educational attainment and must be accounted for when assessing the impact of poor mental health. There are also indirect pathways which may moderate the effects of poor mental health on attainment. For example, the home environment and specifically parental interest in schooling has been associated with higher attainment,(11) as have positive environmental “school effects”.(12) What is less clear is the extent to which differential exposure to these factors also underpin disparities in mental health, and whether resulting differences in mental health might mediate differences in attainment.

International research has demonstrated numerous associations between mental health and educational attainment. (5,13–16) However, the majority of these studies are cross-sectional, making an assessment of causality problematic. Longitudinal data are required to better understand the competing pathways of influence. There is some limited evidence of longitudinal associations between psychological distress in early adolescence and achievement at GCSE.(17,18) Similarly, poor mental health between ages 13 and 15 has been shown to be associated with low GCSE attainment and later

For peer review only

unemployment , demonstrating how the effects of poor early life mental health can extend into adulthood.

Though these findings support the association between mental health and educational outcomes, however, they are contextually limited to England in 2004 (6) or of low generalisability being based on regional data.(17) More recent studies have been based on self-selected samples of respondents and were unable to account for a range of potentially explanatory factors.(18) There is need for an up-to-date examination of nationally representative data. Therefore, this study uses the contemporary nationally representative UK Household Longitudinal Study linked to official education records to test association poor mental health and poor educational attainment. The study is significant in estimating the extent to which mental health in early adolescence has an independent association with attainment at age 16. Robust evidence of a causal relationship between poor mental health and lower academic attainment could be crucial in inspiring investment in researching “what works” in supporting children and adolescents’ mental health. Although schools already appreciate the importance of supporting pupils’ health and wellbeing,(20) a proven link to academic outcomes could also encourage education policymakers and schools to invest more in mental health.

For peer review only

METHODS

UK Household Longitudinal Study (UKHLS)

The UKHLS is a nationally representative household panel survey which began in 2009, aiming to understand social and economic change in Britain at the household and individual levels. Each wave of the survey collects information on approximately 100,000 individuals from 40,000 households, with adult household residents (aged 16 and over) responding using computer-assisted interview and self-completion questionnaire. Young people aged between 10-15 were offered a self-completion questionnaire. Further detail on the sampling design and data collection is available.(21) Administrative national educational records from the from the National Pupil Database (NPD) (22) for school-age children between ages 3 and 18 were linked to the UKHLS if parents and their children were living in England and consented to linkage at wave 1. Linkage consent rates do not differ systematically by parental class, or parental education though they are lower within ethnic minority groups which is consistent with other cohort studies.(23)

This analysis used a nationally representative sample of 11 to 14 year olds present at wave 1 (2009-2011) and wave 3 (2011-2013) linked to the NPD. Where respondents were present at both waves, data from wave 3 was selected as the respondent was further into adolescence. Over half (58%) of the eligible sample of 11 to 14 year olds at wave 1 or wave 3 of UKHLS consented to having their data linked (N=3675) while 18% of the eligible sample was successfully linked (N=1110).

Educational attainment

The primary outcome was a binary variable indicating low educational attainment, defined as whether the young person achieved 5 or more grades A*-C for the General Certificate of Secondary Education (GCSE), including English and maths. This was the benchmark measure of educational attainment at secondary schools in England during the study period.(24)

Mental health - socioemotional difficulties

Young people completed the Strengths and Difficulties questionnaire (SDQ) validated for ages 4-15 years.(25) The SDQ asks questions about five domains of behaviour, namely: conduct problems; hyperactivity; emotional symptoms; peer problems and pro-social behaviour. Scores from the conduct problems, hyperactivity, emotional symptoms and peer problems subscales were summed to construct a total difficulties score, where a higher score refers to a greater level of socioemotional difficulties. A clinically relevant cut point defining difficulties was assigned at 18 or more out of a possible 40.

For peer review only

Explanatory variables

We focussed on risk factors where the literature has established potentially causal associations with educational attainment and mental health respectively. All analyses were controlled for gender, age, ethnic group as well as the household's highest parental social class, household deprivation and mother's educational qualifications.(26–28) Parents' highest current or previous occupational social class was based on the National Statistics Socioeconomic Classification (NS-SEC). This schema was collapsed into a three-tier hierarchical scale,(29) with an additional category for parents who had never held a job. The mother's highest qualification was summarised on a three-tier hierarchical scale, with an additional category for overseas or no qualifications. Household poverty was derived based on income poverty, material poverty, subjective poverty and the receipt of benefits and was categorised into 'not at all deprived', 'somewhat deprived' or 'highly deprived'.(30) Additionally, family type was grouped into two parent households, lone parent household or other family types.(26)

Parental relationships were assessed using a binary measures of young people's self-reports on how interested their parent(s) are at how they do at school, attendance at parents' evenings, frequency of quarrelling with either parent(s) and how often they feel supported by their family.(11) Parental physical and mental health was assessed (31) using the SF-12 Physical and Mental Component Summary respectively,(32) with scores in lowest quintile representing poor physical health and a mental health score of >=45.6 representing poor mental health.(33)

Young people reported levels of happiness specifically with school-work as well as with school generally on a 7-point scale with a score of 5 or greater indicating happiness.(34) Prior attainment was measured based on whether young people achieved the expected level 4 reading, writing and mathematics at Key Stage 2 (ages 7 to 11 years).(35)

The final sample consisted of all youth panel respondents aged 11 to 14 years with data on mental health and life satisfaction in wave 1 or wave 3 of UKHLS as well as NPD data on GCSE scores at ages 15 or 16 years (N=1110). The analytic sample covers England only due to the limited geographical coverage of the NPD.

Statistical analysis

Multiple imputation was used to account for missing data under the missing at random assumption. All explanatory variables with missing data were imputed. Given that the proportion of missing values ranged from 1% to 16% of the final sample linked to GCSE data, twenty imputed datasets were created. Data on GCSE grades were not imputed due to a high proportion of missing data (70%) due to a lack of linkage consent, and for ethical reasons given these individuals had not consented to their data being used for educational research.

For peer review only

Logistic regression was used to estimate the impact of mental health and other explanatory factors on the odds of not achieving 5 A*-C GCSE grades including English and mathematics. Stepwise regression models adjusted these odds to examine the relative impact of prior attainment, sociodemographic factors, parent-child relationships, young person's happiness with school and parental health on educational attainment. Data was weighted using the cross-sectional self-completion weights in the UKHLS youth panel in wave 1 and wave 3. All analyses were performed in Stata v16.1 (StataCorp, College Station, TX, USA).

For peer review only

Results

The proportion of young people not achieving the KS4 benchmark of 5 GCSEs A*-C including English and maths varied by selected characteristics (Table 1). Low prior attainment at KS2 was most strongly associated with not reaching educational benchmark at KS4. Low attainment was associated with lower social class, lower maternal education, higher household poverty scores and poorer parent-child relationships as well as poor parental mental and physical health. Reported unhappiness with school and school work, and lower parental involvement in schooling was also significantly associated with low attainment.

Table 1: Prevalence % and odds ratio (95% confidence intervals) for low educational attainment at Key Stage 4 by sociodemographic and parental characteristics.

	Unweighted N	Low attainment %	Odds ratio	95% CI
Sex				
Male	550	42.0	1	Reference
Female	560	31.5	0.64***	[0.49,0.83]
Age (years)				
11	14	65.5	3.42*	[1.05,11.15]
12	111	38.4	1.12	[0.72,1.76]
13	432	37.3	1.07	[0.81,1.42]
14	553	35.7	1.00	Reference
Ethnic group				
White British	839	36.9	1	Reference
Other ethnic group	271	37.0	1.00	[0.72,1.40]
Parental highest social class (NS-SEC)				
Management & professional	439	23.4	1.00	Reference
Intermediate	253	34.2	1.70**	[1.19,2.44]
Routine & manual	345	53.6	3.79***	[2.74,5.25]
Unemployed	53	61.3	5.18***	[2.60,10.35]
Mother's highest qualification				
Degree or higher	351	24.0	1	Reference
A-level or equivalent	185	21.8	0.88	[0.57,1.38]
GCSE or equivalent	309	41.3	2.23***	[1.57,3.19]
None/other	239	65.4	6.00***	[4.06,8.86]
Household poverty score				
Not at all deprived	179	16.2	1	Reference
Somewhat deprived	493	35.8	2.89***	[1.84,4.56]
Highly deprived	266	56.5	6.74***	[4.08,11.13]
Family composition				
Two-parent	759	32.9	1	Reference
Single parent	321	47.6	1.86***	[1.39,2.47]
Other	30	suppressed	-	-
Happy with school-work				

For peer review only

Happy	840	29.6	1	Reference
Not happy	263	58.6	3.38***	[2.49,4.57]
Happy with school				
Happy	876	32.0	1	Reference
Not happy	220	54.7	2.57***	[1.86,3.53]
Parental interest in school				
Always or nearly always	871	34.4	1	Reference
Sometimes or rarely	220	46.4	1.66**	[1.20,2.28]
Regularly attends parents' evenings				
Always or nearly always	896	29.6	1	Reference
Sometimes or rarely	199	68.0	5.05***	[3.56,7.16]
Feels supported by family				
Always or mostly	837	34.7	1	Reference
Not supported	269	44.1	1.49*	[1.10,2.02]
Regularly quarrels with either parent				
Less than once a week	662	33.1	1	Reference
More than once a week	423	42.6	1.50**	[1.14,1.97]
Either parent has poor mental health				
No	539	30.0	1	Reference
Yes	423	46.0	1.98***	[1.50,2.62]
Either parent has poor physical health				
No	564	32.9	1	Reference
Yes	402	42.6	1.52**	[1.15,2.00]
Low attainment at Key Stage 2 Maths				
No	169	26.6	1	Reference
Yes	860	85.9	16.92***	[10.65,26.87]
Low attainment at Key Stage 2 Writing				
No	270	22.2	1	Reference
Yes	759	73.9	9.96***	[7.14,13.90]
Low attainment at Key Stage 2 Reading				
No	74	32.4	1	Reference
Yes	947	91.5	22.65***	[9.85,52.09]

Notes: Prevalence and odds ratios (95% CIs) based on imputed and weighted values; low educational attainment defined as < 5 GCSEs at A*-C including English and maths; some values are suppressed due to small base sizes and risk of disclosure

For peer review only

There was a similar patterning to the prevalence of poor mental health. Poorer household socioeconomic circumstances, parental engagement with school and health, parent-child relationships and young person's happiness with school and school work were all significantly associated with increased odds of being classified with poor mental health. However, there was no significant difference in the prevalence of mental health difficulties by sex, and the association between prior attainment and current socioemotional difficulties was relatively weak and significant only for writing at KS2.

Table 2: Prevalence % and odds ratio (95% confidence intervals) of mental health difficulties by selected characteristics (weighted %).

	Unweighted N	SDQ total score>=18 %	Odds ratio	95% CI
Sex				
Male	550	12.1	1	Ref
Female	560	15.0	1.28	[0.88,1.86]
Age (years)				
11	14	35.3	3.79*	[1.11,12.93]
12	111	18.4	1.57	[0.86,2.86]
13	432	12.9	1.03	[0.68,1.55
14	553	12.6	1	Ref
Ethnic group				
White British	839	14.1	1	Ref
Other ethnic group	271	9.6	0.65	[0.37,1.11]
Parental highest social class (NS-SEC)				
Management & professional	439	9.0	1	Ref
Intermediate	253	14.0	1.64	[0.99,2.74]
Routine & manual	345	17.3	2.11**	[1.34,3.33]
Unemployed	53	26.9	3.71**	[1.56,8.84]
Mother's highest qualification				
Degree or higher	351	11.1	1	Ref
A-level or equivalent	185	11.1	1.00	[0.55,1.84]
GCSE or equivalent	309	13.3	1.23	[0.75,2.01]
None/other	239	20.0	2.00**	[1.20,3.33]
Household poverty score				
Not at all deprived	179	8.0	1	Ref
Somewhat deprived	493	11.6	1.50	[0.78,2.88]
Highly deprived	266	22.1	3.26***	[1.67,6.36]
Family composition				
Two-parent	759	12.0	1	Ref
Single parent	321	18.5	1.66*	[1.12,2.47]
Other	30	suppressed	-	-
Happy with school-work				
Happy	840	9.0	1	Ref
Not happy	263	26.8	3.71***	[2.52,5.47]

For peer review only

Happy with school				
Happy	876	9.3	1	Ref
Not happy	220	28.9	3.96***	[2.66,5.90]
Parental interest in school				
Always or nearly always	871	10.6	1	Ref
Sometimes or rarely	220	24.4	2.73***	[1.81,4.10]
Regularly attends parents' evenings				
Always or nearly always	896	10.8	1	Ref
Sometimes or rarely	199	24.9	2.73***	[1.79,4.16]
Feels supported by family				
Always or mostly	837	9.0	1	Ref
Not supported	269	27.8	3.87***	[2.62,5.71]
Regularly quarrels with either parent				
Less than once a week	662	7.5	1	Ref
More than once a week	423	22.5	3.59***	[2.40,5.36]
Either parent has poor mental health				
No	539	11.3	1	Ref
Yes	423	16.4	1.55*	[1.02,2.36]
Either parent has poor physical health				
No	564	11.3	1	Ref
Yes	402	16.6	1.57*	[1.04,2.37]
Low attainment at Key Stage 2 Maths				
No	169	12.5	1	Ref
Yes	860	18.2	1.56	[0.98,2.48]
Low attainment at Key Stage 2 Writing				
No	270	11.5	1	Ref
Yes	759	18.4	1.72**	[1.15,2.58]
Low attainment at Key Stage 2 Reading				
No	74	13.4	1	Ref
Yes	947	15.1	1.15	[0.56,2.37]

Notes: Prevalence and odds ratios (95% CIs) based on imputed and weighted values; some values are suppressed due to small base sizes and risk of disclosure

Young people classified with mental health difficulties were over three times more likely to not reach the KS4 GCSE benchmark (OR 3.11, 95% CI [2.11-4.57]) in the unadjusted model. Incrementally controlling for prior attainment and household socioeconomic factors did not attenuate this risk. Controlling for a young person's happiness with school and school-work (Model 5) and parental relationships and support (Model 6) partially diminished this risk. However, the fully adjusted model demonstrated that young people with poor mental health were over twice as likely (OR 2.05, 95% CI [1.15-3.68]) to not reach the educational benchmark than their counterparts with sub-clinical difficulties. Within individual sub-domains, the fully adjusted model could not account for the higher odds of not reaching the educational benchmark for those with hyperactivity disorder (OR 2.38, 95% CI [1.48-3.82]). For emotional and peer disorders, these risks were no longer significant once adjusted for prior attainment and

sociodemographic factors, and conduct disorder no longer predicted lower attainment following adjustment for happiness with school and school-work.

Table 3: Odds ratios for low attainment at Key Stage 4 by total mental health difficulties and domain scores, adjusted stepwise for explanatory factors.

	Model 1	Model 2	Model 3	Model 4	Model 5	Model 6	Model 7
**Emotional**	1.64* [1.11,2.41]	1.88** [1.27,2.78]	1.75* [1.07,2.85]	1.55 [0.91,2.65]	1.22 [0.71,2.10]	1.12 [0.63,1.99]	1.07 [0.61,1.90]
**Peer**	2.44*** [1.66,3.58]	2.45*** [1.66,3.61]	1.67* [1.02,2.75]	1.50 [0.88,2.55]	1.31 [0.78,2.20]	1.26 [0.74,2.16]	1.20 [0.70,2.08]
**Conduct**	1.92*** [1.33,2.76]	1.83** [1.26,2.65]	1.91** [1.22,3.01]	1.65* [1.02,2.67]	1.25 [0.74,2.11]	1.10 [0.62,1.94]	1.07 [0.60,1.90]
**Hyperactivity**	2.52*** [1.80,3.52]	2.46*** [1.75,3.45]	2.77*** [1.84,4.18]	2.94*** [1.89,4.57]	2.39*** [1.52,3.78]	2.35*** [1.46,3.78]	2.38*** [1.48,3.82]
**Total score**	3.11*** [2.11,4.57]	3.25*** [2.20,4.80]	3.55*** [2.22,5.70]	3.20*** [1.90,5.37]	2.38** [1.38,4.12]	2.10* [1.17,3.77]	2.05* [1.15,3.68]

Note: Imputed model, N=1100
Model 1: unadjusted odds of low KS4 attainment
Model 2: adjusts for Model 1 + age, sex, ethnicity
Model 3: adjusts for Model 2 + prior attainment at KS2
Model 4: adjusts for Model 3 + household social class, maternal education, household poverty, family composition
Model 5: adjusts for Model 4 + happy with school work, happy with school
Model 6: adjusts Model 5 + parental interest in school, parents attend parent evening, family support, quarrels with parents
Model 7: adjusts for Model 6 +parental mental and physical health

Table 4 describes the sex-specific association between mental health difficulties and attainment to explore the well-established and significantly lower level of attainment in males than females observed in table 1. There was an independent relationship between poor mental health and low attainment in males after controlling for all explanatory variables (OR 2.77, [1.30 to 6.29]). For females, the relationship between poor mental health and low attainment was no longer significant once prior attainment, sociodemographic factors and school enjoyment and parental support and engagement with school was controlled for.

For peer review only

For both sexes there were significant and generally strong associations between sub-domains of mental health and attainment. The single noteworthy exception was a lack of association with attainment in females with emotional disorder (OR 1.49, [0.91-2.43]). With exception to hyperactivity, there were no significant associations with attainment in males and females after adjusting for sociodemographic factors and school enjoyment. Hyperactivity predicted poor academic attainment for males (OR 2.17, 95% CI 1.13 to 4.19) and females (OR 2.85, 95% CI 1.24 to 6.03) after controlling for the effects of all explanatory variables.

For peer review only

Table 4: Sex differences in odds ratios for low attainment at Key Stage 4 by total mental health difficulties and domain scores, adjusted stepwise for explanatory factors.

	Emotional		Peer		Conduct		Hyperactivity		Total score	
	Males	Females	Males	Females	Males	Females	Males	Females	Males	Females
Model 1	3.07** [1.48,6.38]	1.49 [0.91,2.43]	2.36** [1.39,4.02]	2.55** [1.45,4.48]	1.65* [1.03,2.66]	2.17** [1.22,3.86]	2.35*** [1.49,3.71]	2.63*** [1.59,4.35]	3.16*** [1.79,5.60]	3.36*** [1.97,5.71]
Model 2	3.22** [1.55,6.71]	1.54 [0.95,2.50]	2.43** [1.43,4.14]	2.78*** [1.59,4.86]	1.71* [1.06,2.76]	2.15* [1.18,3.89]	2.40*** [1.51,3.79]	2.61*** [1.56,4.37]	3.30*** [1.85,5.87]	3.47*** [2.03,5.92]
Model 3	2.91* [1.17,7.22]	1.41 [0.77,2.57]	2.34* [1.20,4.56]	1.31 [0.61,2.79]	1.56 [0.86,2.85]	2.67** [1.35,5.26]	2.40** [1.36,4.24]	3.45*** [1.90,6.27]	3.39*** [1.74,6.62]	3.91*** [2.04,7.51]
Model 4	2.89* [1.05,7.92]	1.23 [0.63,2.42]	2.23* [1.07,4.63]	1.26 [0.55,2.90]	1.44 [0.76,2.72]	2.42* [1.16,5.05]	2.68** [1.43,5.04]	3.43*** [1.75,6.73]	3.38** [1.64,6.98]	3.52*** [1.69,7.32]
Model 5	2.37 [0.85,6.59]	0.98 [0.49,1.97]	1.9 [0.93,3.87]	1.12 [0.48,2.60]	1.03 [0.50,2.12]	1.82 [0.85,3.93]	2.23* [1.15,4.31]	2.64** [1.35,5.18]	2.66* [1.25,5.70]	2.61* [1.22,5.57]
Model 6	2.51 [0.87,7.28]	0.76 [0.35,1.65]	1.85 [0.88,3.90]	1.09 [0.45,2.64]	0.98 [0.45,2.14]	1.35 [0.54,3.32]	2.17* [1.13,4.19]	2.73* [1.24,6.03]	2.86** [1.30,6.29]	1.79 [0.76,4.25]
Model 7	2.36 [0.83,6.64]	0.73 [0.34,1.57]	1.79 [0.83,3.84]	0.99 [0.41,2.40]	0.93 [0.42,2.05]	1.29 [0.52,3.18]	2.17* [1.11,4.23]	2.85** [1.30,6.23]	2.77* [1.24,6.16]	1.69 [0.72,3.95]

Note: Imputed model, Males N=550; Females N=560
Model 1: unadjusted odds of socioemotional difficulties; Model 2: adjusts for Model 1 + age, ethnicity; Model 3: adjusts for Model 2 + prior attainment at KS2; Model 4: adjusts for Model 3 + household social class, maternal education, household poverty, family composition Model 5: adjusts for Model 4 + happy with school work, happy with school; Model 6: adjusts Model 5 + parental interest in school, parents attend parent evening, family support, quarrels with parents; Model 7: adjusts for Model 6 +parental mental and physical health

For peer review only - http://bmjopen.bmj.com/site/about/guidelines.xhtml

For peer review only

DISCUSSION

This nationally representative sample of adolescents observed a strong longitudinal association between mental health difficulties between the ages of 11 and 14 and later educational attainment at age 16. After accounting for a range of confounding and mediating variables, young people with poor mental health were twice as likely to not reach the educational benchmark in England.

It is noteworthy that although prior attainment and family socioeconomic circumstances are well-established predictors of later performance at school (36) they did not explain the independent association between poor mental health difficulties and later attainment. This suggests that the impact of poor mental health in previous high achievers is likely to be as great as it is in those with previously low levels of attainment. In the same way, poor mental health is associated with educational performance to the same extent in young people from more advantaged social backgrounds as it is in those from poorer backgrounds. This implies that improving mental health in early adolescence may be an effective, indirect mechanism for narrowing the socioeconomic gap in attainment. Although the association between poorer mental health and lower attainment operates regardless of socioeconomic background, interventions to improve mental health will disproportionately involve those from disadvantaged backgrounds as they are more likely to experience mental health difficulties, potentially increasing average attainment levels within this group to a greater extent than within the majority population who are not disadvantaged. The potential effect at a population level would be to reduce the average difference in attainment between socioeconomic groups, and narrow educational and consequent social inequalities.

These data also suggest that parental engagement with school, parent-young person relationships and the extent to which young people were happy with school and school-work partially explain the link between socioemotional health and attainment. Though it is possible that happiness with school and school-work is correlated with the overall level socioemotional difficulties and possibly mediates the association with attainment, the proportion classified as having difficulties and also unhappy with school was broadly similar to those who also had poor relationships with their parents and whose parents were disengaged with school. Previous work suggests an independent relationship between socioemotional health and school functioning with the two factors instead mediated by overall life satisfaction,(37) this implies that happiness with school and school-work is unlikely to represent the same construct as socioemotional health.These factors could be explored as future intervention points to improve attainment irrespective of a young person's socioemotional health, operating independently of wider social determinants such as parental education or social deprivation.

For peer review only

While the prevalence of socioemotional difficulties did not vary significantly by sex, there were a significant differences between males and females in the manner in which these difficulties were assiociated with educational attainment. Although males and females were equally likely to not achieve the GCSE benchmark if they were in poor socioemotional health, the likelihood of not achieving the benchmark diminishes for females after controlling for explanatory factors, whereas the relationship remains significant for males. This is concurrent with previous work on the same sample assessing educational attainment at age 18,(38) which controlled for similar explanatory factors. However, in contrast to our findings, females at age 18 exhibited a weak relationship between socioemotional difficulties and attainment than males even though they were significantly more likely to experience poor socioemotional health, with females being more likely to be conscientious high achievers suggested as a possible explanation. Although the reason for this difference needs further investigation, these findings confirm important age and sex differences which ought to be accounted for when devising interventions aimed at promoting adolescent socioemotional health.

Limitations

Consent to data linkage and successful linkage between the UKHLS and the NPD was predicted ethnicity, household structure and social class. The inclusion of these variables in the imputation and the final models may mitigate against some of these selection effects, the lack of an analytic weight and the ethical limitation of being unable to impute missing data for sensitive information which has been actively protected by the respondent means that data is unlikely to be nationally representative; prevalence estimates should be interpreted cautiously. This does not, however, diminish confidence in the potentially causal associations identified by the prospective approach taken, bolstered through adjusting for prior attainment within explanatory models. The use of socioemotional data collected from young people rather than their parents was a strength of this study but other measures of wellbeing and mental health ought to be considered in future analysis as associations with different constructs may differ those presented here.

Contributions

NS and LM designed the analysis which was carried out by LM and MA under guidance from MS and NS. AH and SS contributed to the study design and drafting of the manuscript.

Declarations of interest

None

Funding. The project was funded by the Economic and Social Research Council Secondary Data Analysis Initiative (ES/R005400/1 to NS).

Competing interests: None declared.

Patient consent: Obtained.

Patient and public involvement:
It was not appropriate or possible to involve patients or the public in the design, or conduct, or reporting, or dissemination plans of our research.

Provenance and peer review: Not commissioned; externally peer reviewed.

For peer review only

For peer review only

For peer review only

For peer review only

For peer review only

For peer review only

1. NHS Digital. Mental Health of Children and Young People in England, 2017. [Internet]. 2018. Available from: https://digital.nhs.uk/data-and-information/publications/statistical/mental-health-of-children-and-young-people-in-england/2017/2017

2. Department of Health. Future in mind - Promoting, protecting and improving our children and young people's mental health and wellbeing [Internet]. 2015 p. 81–81. Available from: www.gov.uk/dh

3. Chowdry H, McBride T. Disadvantage, Behaviour And Cognitive Outcomes: Longitudinal Analysis From Age 5 To 16. London; 2017.

4. Sagatun A, Heyerdahl S, Wentzel-Larsen T, Lien L. Mental health problems in the 10th grade and non-completion of upper secondary school: the mediating role of grades in a population-based longitudinal study. BMC public health. 2014;14(1):16–16.

5. Woodward LJ, Fergusson DM. Childhood peer relationship problems and later risks of educational under-achievement and unemployment. Journal of child psychology and psychiatry, and allied disciplines. 2000 Feb;41(2):191–201.

6. Hale DR, Viner RM. How adolescent health influences education and employment: investigating longitudinal associations and mechanisms. J Epidemiol Community Health. 2018 Jun 1;72(6):465–70.

7. Crawford C, Greaves E. Socio-economic, ethnic and gender differences in HE participation Socio-economic, ethnic and gender differences in HE participation Report Authors Socio-economic, ethnic and gender differences in HE participation. 2015 p. 116–116.

8. Department for Education. GCSE and equivalent results: 2017 to 2018 (provisional) [Internet]. GOV.UK. 2019 [cited 2020 Apr 7]. Available from: https://www.gov.uk/government/statistics/gcse-and-equivalent-results-2017-to-2018-provisional

9. Lessof C, Ross A, Brind R. Multiple disadvantage and KS4 attainment: evidence from LSYPE2 [Internet]. GOV.UK. 2019 [cited 2020 Apr 7]. Available from: https://www.gov.uk/government/publications/multiple-disadvantage-and-ks4-attainment-evidence-from-lsype2

10. Schoon I. Planning for the Future: Changing Education Expectations in Three British Cohorts. Historical Social Research [Internet]. 2010;35(2). Available from: http://www.jstor.org/stable/20762452?seq=1#page_scan_tab_contents

11. Flouri E. Parental interest in children's education, children's self-esteem and locus of control, and later educational attainment: twenty-six year follow-up of the 1970 British Birth Cohort. The British journal of educational psychology. 2006;76(Pt 1):41–55.

12. Sellström E, Bremberg S. Is there a "school effect" on pupil outcomes? A review of multilevel studies. J Epidemiol Community Health. 2006 Feb;60(2):149–55.

For peer review only

13. Biederman J, Monuteaux MC, Doyle AE, Seidman LJ, Wilens TE, Ferrero F, et al. Impact of Executive Function Deficits and Attention-Deficit/Hyperactivity Disorder (ADHD) on Academic Outcomes in Children. Journal of Consulting and Clinical Psychology. 2004 Oct;72(5):757–66.

14. Fergusson DM, Woodward LJ, D W, M K, PM L, AC P, et al. Mental Health, Educational, and Social Role Outcomes of Adolescents With Depression. Archives of General Psychiatry. 2002 Mar;59(3):225–225.

15. Kessler C, Saunders B, Ph D, Foster L, Stang PE, Al E. Social Consequences of Psychiatric Disorders, I: Educational Attainment. American Journal of Psychiatry. 1995;15(July):1026–32.

16. Fleming M, Fitton CA, Steiner MFC, McLay JS, Clark D, King A, et al. Educational and health outcomes of children and adolescents receiving antidepressant medication: Scotland-wide retrospective record linkage cohort study of 766 237 schoolchildren. Int J Epidemiol [Internet]. 2020 Feb 19 [cited 2020 Apr 7]; Available from: https://academic.oup.com/ije/advance-article/doi/10.1093/ije/dyaa002/5740768

17. Rothon C, Head J, Clark C, Klineberg E, Cattell V, Stansfeld S. The impact of psychological distress on the educational achievement of adolescents at the end of compulsory education. Social Psychiatry and Psychiatric Epidemiology. 2009 May 1;44(5):421–7.

18. Deighton J, Humphrey N, Belsky J, Boehnke J, Vostanis P, Patalay P. Longitudinal pathways between mental health difficulties and academic performance during middle childhood and early adolescence. The British journal of developmental psychology. 2018 Mar 1;36(1):110–26.

19. Hale D, Viner R. G492[Health in adolescence influences educational attainments and life chances: longitudinal associations in the longitudinal study of young people in england (LSYPE). Archives of Disease in Childhood. 2015 Apr 27;100(Suppl 3):A210.2-A211.

20. Marshall L, Wishart R, Dunatchik A, Smith N. Supporting Mental Health in Schools and Colleges [Internet]. 2017 [cited 2020 Jul 4]. Available from: https://assets.publishing.service.gov.uk/government/uploads/system/uploads/attachment_data/file/634726/Supporting_Mental-Health_survey_report.pdf

21. Institute for Social and Economic Research. Understanding Society: The UK Household Longitudinal Study, Waves 1-9 - User Guide [Internet]. University of Essex; 2019 [cited 2020 Sep 4]. Available from: https://www.understandingsociety.ac.uk/sites/default/files/downloads/documentation/mainstage/user-guides/mainstage-user-guide.pdf

22. Jay MA, Grath-Lone LM, Gilbert R. Data Resource: the National Pupil Database (NPD). International Journal of Population Data Science [Internet]. 2019 Mar 20 [cited 2020 Apr 9];4(1). Available from: https://ijpds.org/article/view/1101

23. Knies G, Burton J. Analysis of four studies in a comparative framework reveals: health linkage consent rates on British cohort studies higher than

on UK household panel surveys. BMC medical research methodology. 2014 Nov;14:125–125.

24. Department for Education. GCSE and equivalent results: 2011 to 2012 (revised) [Internet]. 2013 Jan [cited 2020 Apr 14]. Available from: https://www.gov.uk/government/statistics/revised-gcse-and-equivalent-results-in-england-academic-year-2011-to-2012

25. Goodman R. The Strengths and Difficulties Questionnaire: a research note. Journal of Child Psychology and Psychiatry. 1997;38(5):581–6.

26. Strand S. The limits of social class in explaining ethnic gaps in educational attainment. British Educational Research Journal. 2011;37(2):197–229.

27. Goodman A, Gregg P. Poorer children's educational attainment: how important are attitudes and behaviour? [Internet]. JRF. 2010 [cited 2020 Apr 7]. Available from: https://www.jrf.org.uk/report/poorer-children%E2%80%99s-educational-attainment-how-important-are-attitudes-and-behaviour

28. Carneiro P, Meghir C, Parey M. Maternal Education, Home Environments, and the Development of Children and Adolescents. Journal of the European Economic Association. 2013 Jan 1;11(suppl_1):123–60.

29. Rose D, Pevalin DJ. The National Statistics socio-economic ,unifying official and sociological approaches to the conceptualisation and measurement of social class. ISER working P 2001-4 ISER working P no 2001-4. Institute for Social and Economic Research, University of Essex,; 2001.

30. Knies G. Income effects on children's life satisfaction: Longitudinal evidence for England [Internet]. ISER Working Paper Series; 2017 [cited 2020 Apr 17]. Report No.: 2017–02. Available from: https://www.econstor.eu/handle/10419/163545

31. Smith M. Parental mental health: disruptions to parenting and outcomes for children. Child & Family Social Work. 2004 Feb 1;9(1):3–11.

32. Ware Jr JE, Kosinski M, Keller SD. A 12-Item Short-Form Health Survey: construction of scales and preliminary tests of reliability and validity. Medical care. 1996;34(3):220–33.

33. Vilagut G, Forero CG, Pinto-Meza A, Haro JM, de Graaf R, Bruffaerts R, et al. The Mental Component of the Short-Form 12 Health Survey (SF-12) as a Measure of Depressive Disorders in the General Population: Results with Three Alternative Scoring Methods. Value in Health. 2013 Jun 1;16(4):564–73.

34. Suldo SM, Riley KN, Shaffer EJ. Academic Correlates of Children and Adolescents' Life Satisfaction. School Psychology International. 2006 Dec 1;27(5):567–82.

For peer review only

35. Department for Education. How do pupils progress through Key Stages 2 and 3? [Internet]. Department for Education; [cited 2020 Apr 17]. Available from: https://assets.publishing.service.gov.uk/government/uploads/system/uploads/attachment_data/file/182413/DFE-RR096.pdf

36. Tom Benton TS. Analysis of use of Key Stage 2 data in GCSE predictions [Internet]. OFQUAL; 2014 [cited 2020 Jul 8]. Available from: https://assets.publishing.service.gov.uk/government/uploads/system/uploads/attachment_data/file/429074/2014-06-16-analysis-of-use-of-key-stage-2-data-in-gcse-predictions.pdf

37. Guzmán J, Green JG, Oblath R, Holt MK. Life Satisfaction Mediates the Association Between Mental Health Risk and Perceptions of School Functioning Among Children and Adolescents. Contemp School Psychol [Internet]. 2019 Sep 3 [cited 2020 Aug 7]; Available from: https://doi.org/10.1007/s40688-019-00257-w

38. Tregaskis O, Nandi A, Watson D. An examination of the relationship between adolescent mental health and educational outcomes in early adulthood: Secondary analysis of Understanding Society data wave 1 to wave 8. 2020.

For peer review only

STROBE 2007 (v4) Statement—Checklist of items that should be included in reports of *cohort studies*

Section/Topic	Item #	Recommendation	Reported on page #
**Title and abstract**	1	(*a*) Indicate the study's design with a commonly used term in the title or the abstract	
		(*b*) Provide in the abstract an informative and balanced summary of what was done and what was found	
**Introduction**			
Background/rationale	2	Explain the scientific background and rationale for the investigation being reported	
Objectives	3	State specific objectives, including any prespecified hypotheses	
**Methods**			
Study design	4	Present key elements of study design early in the paper	
Setting	5	Describe the setting, locations, and relevant dates, including periods of recruitment, exposure, follow-up, and data collection	
Participants	6	(*a*) Give the eligibility criteria, and the sources and methods of selection of participants. Describe methods of follow-up	
		(*b*) For matched studies, give matching criteria and number of exposed and unexposed	
Variables	7	Clearly define all outcomes, exposures, predictors, potential confounders, and effect modifiers. Give diagnostic criteria, if applicable	
Data sources/ measurement	8*	For each variable of interest, give sources of data and details of methods of assessment (measurement). Describe comparability of assessment methods if there is more than one group	
Bias	9	Describe any efforts to address potential sources of bias	
Study size	10	Explain how the study size was arrived at	
Quantitative variables	11	Explain how quantitative variables were handled in the analyses. If applicable, describe which groupings were chosen and why	
Statistical methods	12	(*a*) Describe all statistical methods, including those used to control for confounding	
		(*b*) Describe any methods used to examine subgroups and interactions	
		(*c*) Explain how missing data were addressed	
		(*d*) If applicable, explain how loss to follow-up was addressed	
		(*e*) Describe any sensitivity analyses	
**Results**			

For peer review only

Participants	13*	(a) Report numbers of individuals at each stage of study—eg numbers potentially eligible, examined for eligibility, confirmed eligible, included in the study, completing follow-up, and analysed	
		(b) Give reasons for non-participation at each stage	
		(c) Consider use of a flow diagram	
Descriptive data	14*	(a) Give characteristics of study participants (eg demographic, clinical, social) and information on exposures and potential confounders	
		(b) Indicate number of participants with missing data for each variable of interest	
		(c) Summarise follow-up time (eg, average and total amount)	
Outcome data	15*	Report numbers of outcome events or summary measures over time	
Main results	16	(*a*) Give unadjusted estimates and, if applicable, confounder-adjusted estimates and their precision (eg, 95% confidence interval). Make clear which confounders were adjusted for and why they were included	
		(*b*) Report category boundaries when continuous variables were categorized	
		(*c*) If relevant, consider translating estimates of relative risk into absolute risk for a meaningful time period	
Other analyses	17	Report other analyses done—eg analyses of subgroups and interactions, and sensitivity analyses	
**Discussion**			
Key results	18	Summarise key results with reference to study objectives	
**Limitations**			
Interpretation	20	Give a cautious overall interpretation of results considering objectives, limitations, multiplicity of analyses, results from similar studies, and other relevant evidence	
Generalisability	21	Discuss the generalisability (external validity) of the study results	
**Other information**			
Funding	22	Give the source of funding and the role of the funders for the present study and, if applicable, for the original study on which the present article is based	

*Give information separately for cases and controls in case-control studies and, if applicable, for exposed and unexposed groups in cohort and cross-sectional studies.

Note: An Explanation and Elaboration article discusses each checklist item and gives methodological background and published examples of transparent reporting. The STROBE checklist is best used in conjunction with this article (freely available on the Web sites of PLoS Medicine at http://www.plosmedicine.org/, Annals of Internal Medicine at http://www.annals.org/, and Epidemiology at http://www.epidem.com/). Information on the STROBE Initiative is available at www.strobe-statement.org.

For peer review only

Adolescent mental health difficulties and educational attainment: findings from the UK Household Longitudinal Study

Journal:	*BMJ Open*
Manuscript ID	bmjopen-2020-046792.R1
Article Type:	Original research
Date Submitted by the Author:	12-May-2021
Complete List of Authors:	SMITH, NEIL; NatCen Social Research, Policy Research Centre<br>Marshall, Lydia; NatCen Social Research, Policy Research Centre<br>Albakri, Muslihah; NatCen Social Research, Policy Research Centre<br>Smuk, Melanie; London School of Hygiene & Tropical Medicine<br>Hagell, Ann; Association for Young People's Health<br>Stansfeld, Stephen; Barts and the London, Queen Marys School of Medicine and Dentistry, Psychiatry
**Primary Subject Heading**:	Epidemiology
Secondary Subject Heading:	Mental health
Keywords:	MENTAL HEALTH, PUBLIC HEALTH, Child & adolescent psychiatry < PSYCHIATRY, EPIDEMIOLOGY

SCHOLARONE™ Manuscripts

I, the Submitting Author has the right to grant and does grant on behalf of all authors of the Work (as defined in the below author licence), an exclusive licence and/or a non-exclusive licence for contributions from authors who are: i) UK Crown employees; ii) where BMJ has agreed a CC-BY licence shall apply, and/or iii) in accordance with the terms applicable for US Federal Government officers or employees acting as part of their official duties; on a worldwide, perpetual, irrevocable, royalty-free basis to BMJ Publishing Group Ltd ("BMJ") its licensees and where the relevant Journal is co-owned by BMJ to the co-owners of the Journal, to publish the Work in this journal and any other BMJ products and to exploit all rights, as set out in our licence.

The Submitting Author accepts and understands that any supply made under these terms is made by BMJ to the Submitting Author unless you are acting as an employee on behalf of your employer or a postgraduate student of an affiliated institution which is paying any applicable article publishing charge ("APC") for Open Access articles. Where the Submitting Author wishes to make the Work available on an Open Access basis (and intends to pay the relevant APC), the terms of reuse of such Open Access shall be governed by a Creative Commons licence – details of these licences and which Creative Commons licence will apply to this Work are set out in our licence referred to above.

Other than as permitted in any relevant BMJ Author's Self Archiving Policies, I confirm this Work has not been accepted for publication elsewhere, is not being considered for publication elsewhere and does not duplicate material already published. I confirm all authors consent to publication of this Work and authorise the granting of this licence.

For peer review only

Adolescent mental health difficulties and educational attainment: findings from the UK Household Longitudinal Study

Corresponding author:
Neil R Smith, PhD
NatCen Social Research
Policy Research Centre
35 Northampton Square
London
EC1V 0AX, UK
Neil.Smith@natcen.ac.uk

Lydia Marshall, PhD
NatCen Social Research
Policy Research Centre
35 Northampton Square
London
EC1V 0AX, UK
Lydia.Marshall@natcen.ac.uk

Muslihah Albakri
NatCen Social Research
Policy Research Centre
35 Northampton Square
London
EC1V 0AX, UK
Muslihah.Albakri@natcen.ac.uk

Melanie Smuk, PhD
London School of Hygiene & Tropical Medicine
Keppel Street
London
WC1E 7HT, UK
Melanie.Smuk@lshtm.ac.uk

Ann Hagell, PhD
Association for Young People's Health
7-14 Great Dover Street
London
SE1 4YR, UK
Ann@youngpeopleshealth.org.uk

Stephen Stansfeld, PhD
Centre for Psychiatry
Bart's and the London School of Medicine and Dentistry
London
EC1M 6BQ, UK
S.A.Stansfeld@qmul.ac.uk

2,961 words
Keywords: mental health, adolescence, education, social epidemiology, life course

For peer review only

ABSTRACT

Objective: This study examines whether there is an independent association between mental difficulties in adolescence and educational attainment at age 16.

Design: Longitudinal study.

Setting: Nationally representative data from the UK Household Longitudinal Study (UKHLS) were linked to the National Pupil Database for England.

Participants: Respondents (N=1,100) to the UKHLS between 2009-2012 were linked to the National Pupil Database to investigate longitudinal associations between mental difficulties at ages 11-14 and educational attainment at age 16 (GCSE).

Primary outcome measure: Not gaining five or more GCSE qualifications at age 16, including English and maths at grade A*-C.

Results: An atypical total mental health difficulties score measured using the Strengths and Difficulties Questionnaire at ages 11-14 predicted low levels of educational attainment at age 16 (OR: 3.11 (95% CI: [2.11, 4.57]). Controlling for prior attainment and family sociodemographic factors, happiness with school(/work) and parental health, school engagement and relationship with the child partially attenuated the association which was significant in the fully adjusted model (2.05, 95% CI: [1.15,3.68]). The association was maintained in the fully adjusted model for males only (OR: 2.77 (95% CI: [1.24, 6.16]) but not for females. Hyperactivity disorder strongly predicted lower attainment for males (OR: 2.17 (95% CI: [1.11, 4.23]) and females (OR: 2.85 (95% CI: [1.30, 6.23]).

Conclusion: Mental difficulties at ages 11-14 was independently linked to educational success at age 16, highlighting an important pathway through which health in adolescence can determine young people's life chances.

For peer review only

STRENGTHS AND LIMITATIONS OF THIS STUDY.

- This is a large, nationally representative longitudinal cohort study containing self-assessed measures of mental health among young people linked to a National Pupil Database of educational records.
- The study captures a diverse range of social, demographic, economic and behavioural factors affecting young people in their home and school environment, permitting statistical adjustment for multiple confounding relationships which might explain the association between mental health and educational attainment.
- Consent to data linkage between the longitudinal study and the National Pupil Database was incomplete, though factors which predicted patterns of non-consent were controlled for within our models.
- Missing data was accounted for using multiple imputation methods which exploited the wide range of associations within the observed data to minimise errors within estimates of effect.

For peer review only

INTRODUCTION

Growing evidence of the prevalence of poor child and adolescent mental health has led to this issue becoming a key policy priority in the UK. Mental ill-health in children and young people in England increases age with around 14.4% of 11-16 years experiencing a mental disorder compared to 5.5% in their pre-school counterparts aged 2-4 years.(1) With 75% of adult mental health problems (excluding dementia) starting by the age of 18,(2) adolescence is a key period in the development of long-lasting mental health difficulties. The UK government's *Future in Mind* report (2) presented an important economic case for investment in early prevention of mental ill-health to mitigate against the costs of longer-term support for health needs. However, this argument neglects the impact that early life mental health potentially has on other early life outcomes fundamental in determining life chances, such as educational attainment.(3) Educational outcomes are closely associated with later-life chances with well-established links to employment, income, housing and offending as well as physical health and on-going mental health disorders. If poor mental health diminishes the capacity for individuals to fulfil their academic potential, mental health itself is likely to be a driver of educational inequality and consequent on-going social inequality.

On the other hand, the association between mental health and educational outcomes might not be direct, but rather incorporate the influence of confounding factors. A range of demographic and socioeconomic factors, such as gender, ethnicity, socioeconomic disadvantage and maternal education and parental health(4–6) have known relationships with educational attainment and must be accounted for when assessing the impact of poor mental health. Similarly, the home environment and specifically parental interest in schooling has been associated with higher attainment,(7) as have positive environmental "school effects",(8) whereas lower attainment has been associated with absence from school (1) or poor classroom behaviours. (9) What is less clear is the extent to which differential exposure to these factors also underpin disparities in mental health, and whether resulting differences in mental health might influence differences in attainment.

International research has demonstrated numerous associations between mental health and educational attainment.(10–12). The evidence base for England is less well-established which is of particular relevance during a time of policy interest in boosting mental health provision in schools.(13) There is some evidence of longitudinal associations between psychological distress in early adolescence and achievement at GCSE in England.(14,15) Similarly, poor mental health between ages 13 and 15 has been shown to be associated with low GCSE attainment and later unemployment, (9) demonstrating how the effects of poor early life mental health can extend into adulthood (16). Though many of these findings support the association between mental health and educational outcomes, they are often of low generalisability being based on regional data or non-probability samples(14) or unable to account for a range of potentially explanatory factors.(15) There appears to be a strengthening of the relationship between adolescent mental health and

For peer review only - http://bmjopen.bmj.com/site/about/guidelines.xhtml

educational outcomes in recent generations (17) so there is a pressing need for an up-to-date examination of nationally representative data for England.

Therefore, this study uses a novel and contemporary data linkage between the nationally representative UK Household Longitudinal Study linked to objectively measured official education records, to test associations between poor mental health and poor educational attainment. The study is significant in estimating the extent to which mental health in early adolescence has an independent association with attainment at age 16 in England in males and females. Robust evidence of a causal relationship between poor mental health and lower academic attainment could be crucial in inspiring investment in researching “what works” in supporting children and adolescents’ mental health. Although schools already appreciate the importance of supporting pupils’ health and wellbeing,(18) a proven link to academic outcomes could also encourage education and public health policymakers to invest more in mental health.

For peer review only

METHODS

UK Household Longitudinal Study (UKHLS)

The UKHLS is a nationally representative household panel survey (19) which began in 2009, aiming to understand social and economic change in Britain at the household and individual levels. Each wave of the survey collects information on approximately 100,000 individuals from 40,000 households, with adult household residents (aged 16 and over) responding using computer-assisted interview and self-completion questionnaire. Young people aged between 10-15 were offered a self-completion questionnaire. Further detail on the sampling design and data collection is available.(20) National educational records from the from the National Pupil Database (NPD) (21) for school-age children between ages 3 and 18 were linked to the UKHLS if parents and their children were living in England and consented to linkage at wave 1. Linkage consent rates did not differ systematically by parental class, or parental education though they were lower within ethnic minority groups which is consistent with other cohort studies.(22)

This analysis used a nationally representative sample of 11 to 14-year olds present at wave 1 (2009-2011) and wave 3 (2011-2013) linked to the NPD. Wave 2 (2010-2012) was excluded as it did not ask for information about mental health. Where respondents were present at both waves, data from wave 3 was selected as the respondent was further into adolescence. Figure 1 tracks the study population down to the final analytic sample.

The final sample consisted of all consenting youth panel respondents aged 11 to 14 years with data on mental health in wave 1 or wave 3 of UKHLS as well as NPD data on GCSE scores at ages 15 or 16 years (N=1110). The analytic sample covers England only due to the limited geographical coverage of the NPD.

Educational attainment

The primary outcome was a binary variable indicating low educational attainment, defined as whether the young person achieved 5 or more grades A*-C for the General Certificate of Secondary Education (GCSE), including English and maths. This was the benchmark measure of educational attainment at Key Stage 4 (KS4) at secondary schools in England during the study period.(23)

Mental difficulties

Young people completed the Strengths and Difficulties questionnaire (SDQ) validated for ages 4-15 years.(24) The SDQ asks questions about four domains of negative behaviours which have varying strengths of association with educational attainment, namely: conduct problems (11); hyperactivity (25); emotional symptoms (14); peer problems.(26) Scores from the four subscales were summed to construct a total difficulties score, where a higher score refers to a greater level of mental difficulties. Binary measures of mental difficulties were derived based on developer guidance. (24) An “atypical” level of total difficulties was derived from the top 10% of the population

For peer review only - http://bmjopen.bmj.com/site/about/guidelines.xhtml

scores (>=18 out of 40) and individual SDQ domains used validated "atypical" cut points which have also been used in a recent prevalence survey in England. (27)

Explanatory variables

We focussed on risk factors where the literature has established potentially causal associations with educational attainment and mental health respectively. All analyses were controlled for gender, age, ethnic group as well as the household's highest parental social class, household deprivation and mother's educational qualifications.(28–30) Parents' highest current or previous occupational social class was based on the National Statistics Socioeconomic Classification (NS-SEC). This schema was collapsed into a three-tier hierarchical scale, with an additional category for parents who had never held a job. The mother's highest qualification was summarised on a three-tier hierarchical scale, with an additional category for overseas or no qualifications. Household poverty was derived based on income poverty, material poverty, subjective poverty and the receipt of benefits and was categorised into 'not at all deprived', 'somewhat deprived' or 'highly deprived'.(31) Additionally, family type was grouped into two parent households, lone parent household or other family types.(28)

Parental relationships were assessed using a binary measures of young people's self-reports on how interested their parent(s) are at how they do at school, attendance at parents' evenings, frequency of quarrelling with either parent(s) and how often they feel supported by their family. (7) Parental physical and mental health was assessed using the SF-12 Physical and Mental Component Summary respectively,(32) with a score from either parent in the lowest quintile representing poor physical health and a mental health score of >=45.6 representing poor mental health.

Young people reported levels of happiness specifically with school-work as well as with school generally on a seven-point scale with a score of five or greater indicating happiness.(33) Prior attainment was measured based on whether young people achieved the expected level 4 reading, writing and mathematics at Key Stage 2 (KS2) (ages 10-11 years).

All non-educational attainment measures were taken at the time adolescent mental health was assessed.

Statistical analysis

Multiple imputation was used to account for missing data under the missing at random assumption. Complete data was available was available for age, sex, ethnicity and family composition and all variables shown in Table 1 were used in the imputation. Missing values for explanatory variables ranged from 1% to 13%, and 0.2% of values for mental difficulties were imputed. Given the low level of missingness, twenty imputed datasets were created. Data on GCSE grades were not imputed due to a high proportion of missing data (70%) due to a lack of linkage consent, and for ethical reasons given these individuals had not consented to their data being used for educational research.

For peer review only

Logistic regression was used to estimate the impact of mental health and other explanatory factors on the odds of not achieving 5 A*-C GCSE grades including English and mathematics. Stepwise regression models adjusted these odds to examine the relative impact of prior attainment, sociodemographic factors, parent-child relationships, young person's happiness with school and parental health on educational attainment. Models were stratified to explore gender differences in mental difficulties. Data was weighted using the cross-sectional self-completion weights in the UKHLS youth panel in wave 1 and wave 3. All analyses were performed in Stata v16.1 (StataCorp, College Station, TX, USA).

For peer review only

For peer review only - http://bmjopen.bmj.com/site/about/guidelines.xhtml

RESULTS

The analytic sample was evenly split by gender and the overwhelming majority were aged 13 to 14 years old. Respondents tended to be from relatively socioeconomically advantaged. A third of mothers were degree educated and 41.8% of households belonged to the highest social class. Over three-quarters of the sample reported high parental engagement with school and happiness with school-work. Prior attainment levels were positive for reading (93.3%), writing (82.6%) and maths (71.5%).

The proportion of young people not achieving the Key Stage 4 (KS4) benchmark of 5 GCSEs A*-C including English and maths varied by selected characteristics (Table 1). Low prior attainment at Key Stage 2 (KS2) was most strongly associated with not reaching the educational benchmark at KS4. Low attainment at KS4 was also associated with lower social class, lower maternal education, higher household poverty scores and poorer parent-child relationships as well as poor parental mental and physical health. Reported unhappiness with school and school-work, and lower parental involvement in schooling was also significantly associated with low attainment.

There was a similar patterning to the prevalence of poor mental health (Table 1). Poorer household socioeconomic circumstances, parental engagement with school and health, parent-child relationships and young person's happiness with school and school-work were all significantly associated with increased odds of being classified with poor mental health. However, there was no significant difference in the prevalence of mental health difficulties by sex, and the association between prior attainment and current mental difficulties was relatively weak and significant only for writing at KS2.

Table 1: Prevalence % of low educational attainment at Key Stage 4 and mental difficulties by sociodemographic and parental characteristics.

	% (N)	Low attainment %	SDQ total score>=18 %
Sex			
Male	51.6 (550)	42.0	12.1
Female	48.4 (560)	31.5***	15.0
Age (years)			
11	1.1 (14)	65.5*	35.3*
12	9.7 (111)	38.4	18.4
13	38.9 (432)	37.3	12.9
14	50.4 (553)	35.7	12.6
Ethnic group			
White British	86.1 (839)	36.9	14.1
Other ethnic group	13.9 (271)	37.0	9.6
Parental highest social class (NS-SEC)			
Management & professional	41.8 (439)	23.4	9.0
Intermediate	22.7 (253)	34.2**	14.0
Routine & manual	31.0 (345)	53.6**	17.3**

For peer review only

Unemployed	4.4 (53)	61.3**	26.9**
Mother's highest qualification			
Degree or higher	33.2 (351)	24.0	11.1
A-level or equivalent	17.5 (185)	21.8	11.1
GCSE or equivalent	29.5 (309)	41.3***	13.3
None/other	19.8 (239)	65.4***	20.0**
Household poverty score			
Not at all deprived	20.9 (179)	16.2	8.0
Somewhat deprived	54.0 (493)	35.8***	11.6
Highly deprived	25.1 (266)	56.5***	22.1***
Family composition			
Two-parent	69.7 (759)	32.9	12.0
Single parent	27.8 (321)	47.6***	18.5*
Other	2.5 (30)	suppressed	suppressed
Happy with school-work			
Happy	74.7 (840)	29.6	9.0
Not happy	25.3 (263)	58.6***	26.8***
Happy with school			
Happy	78.6 (876)	32.0	9.3
Not happy	21.4 (220)	54.7***	28.9***
Parental interest in school			
Always or nearly always	79.0 (871)	34.4	10.6
Sometimes or rarely	21.0 (220)	46.4**	24.4***
Regularly attends parents' evenings			
Always or nearly always	81.1 (896)	29.6	10.8
Sometimes or rarely	18.9 (199)	68.0***	24.9***
Feels supported by family			
Always or mostly	76.3 (837)	34.7	9.0
Not supported	23.7 (269)	44.1*	27.8***
Regularly quarrels with either parent			
Less than once a week	60.0 (662)	33.1	7.5
More than once a week	40.0 (423)	42.6**	22.5***
Parental mental health			
Not poor	56.8 (539)	30.0	11.3
Poor	43.2 (423)	46.0***	16.4*
Parental physical health			
Not poor	58.6 (564)	32.9	11.3
Poor	41.4 (402)	42.6**	16.6*
Attainment at Key Stage 2 Maths			
Not Low	71.5 (860)	26.6	12.5
Low	17.4 (169)	85.9***	18.2
Attainment at Key Stage 2 Writing			
Not Low	82.6 (270)	22.2	11.5
Low	28.4 (759)	73.9***	18.4**

For peer review only - http://bmjopen.bmj.com/site/about/guidelines.xhtml

Attainment at Key Stage 2 Reading			
Not Low	92.3 (947)	32.4	13.4
Low	7.7 (74)	91.5***	15.1

Notes: Unweighted N; Imputed and weighted percentages shown; low educational attainment defined as < 5 GCSEs at A*-C including English and maths; some values are suppressed due to small base sizes and risk of disclosure; *** p<0.001, **p<0.01, *p<0.05

Young people classified with mental health difficulties were over three times more likely to not reach the KS4 GCSE benchmark (OR 3.11, 95% CI [2.11-4.57]) in the unadjusted model (Table 2). Incrementally controlling for prior attainment and household socioeconomic factors did not attenuate this risk. Controlling for a young person's happiness with school and school-work (Model 5) and parental relationships and support (Model 6) partially diminished this risk. However, the fully adjusted model demonstrated that young people with poor mental health were over twice as likely (OR 2.05, 95% CI [1.15-3.68]) to not reach the educational benchmark than their counterparts with sub-clinical difficulties. Within individual sub-domains, the fully adjusted model could not account for the higher odds of not reaching the educational benchmark for those with hyperactivity disorder (OR 2.38, 95% CI [1.48-3.82]), implying that hyperactivity is the behaviour which largely drives the association between mental difficulties scores and lower attainment. For emotional and peer disorders, these risks were no longer significant once adjusted for prior attainment and sociodemographic factors, and conduct disorder no longer predicted lower attainment following adjustment for happiness with school and school-work.

Table 2: Odds ratios for low attainment at Key Stage 4 by total mental health difficulties and domain scores, adjusted stepwise for explanatory factors.

	**Model 1**	**Model 2**	**Model 3**	**Model 4**	**Model 5**	**Model 6**	**Model 7**
**Emotional**	1.64* [1.11,2.41]	1.88** [1.27,2.78]	1.75* [1.07,2.85]	1.55 [0.91,2.65]	1.22 [0.71,2.10]	1.12 [0.63,1.99]	1.07 [0.61,1.90]
**Peer**	2.44*** [1.66,3.58]	2.45*** [1.66,3.61]	1.67* [1.02,2.75]	1.50 [0.88,2.55]	1.31 [0.78,2.20]	1.26 [0.74,2.16]	1.20 [0.70,2.08]
**Conduct**	1.92*** [1.33,2.76]	1.83** [1.26,2.65]	1.91** [1.22,3.01]	1.65* [1.02,2.67]	1.25 [0.74,2.11]	1.10 [0.62,1.94]	1.07 [0.60,1.90]
**Hyperactivity**	2.52*** [1.80,3.52]	2.46*** [1.75,3.45]	2.77*** [1.84,4.18]	2.94*** [1.89,4.57]	2.39*** [1.52,3.78]	2.35*** [1.46,3.78]	2.38*** [1.48,3.82]
**Total score**	3.11*** [2.11,4.57]	3.25*** [2.20,4.80]	3.55*** [2.22,5.70]	3.20*** [1.90,5.37]	2.38** [1.38,4.12]	2.10* [1.17,3.77]	2.05* [1.15,3.68]

Note: Imputed model, N=1100
Model 1: unadjusted odds of low KS4 attainment

For peer review only

Model 2: adjusts for Model 1 + age, sex, ethnicity
Model 3: adjusts for Model 2 + prior attainment at KS2
Model 4: adjusts for Model 3 + household social class, maternal education, household poverty, family composition
Model 5: adjusts for Model 4 + happy with school work, happy with school
Model 6: adjusts Model 5 + parental interest in school, parents attend parent evening, family support, quarrels with parents
Model 7: adjusts for Model 6 +parental mental and physical health
*** p<0.001, **p<0.01, *p<0.05

Table 3 describes the sex-specific association between mental health difficulties and attainment to explore the well-established and significantly lower level of attainment in males than females observed in Table 1. There was an independent relationship between poor mental health and low attainment in males after controlling for all explanatory variables (OR 2.77, [1.30 to 6.29]). For females, the relationship between poor mental health and low attainment was no longer significant once prior attainment, sociodemographic factors and school enjoyment and parental support and engagement with school was controlled for.

For both sexes there were significant and generally strong associations between sub-domains of mental health and attainment. The single noteworthy exception was a lack of association with attainment in females with emotional disorder (OR 1.49, [0.91-2.43]). With exception to hyperactivity, there were no significant associations with attainment in males and females after adjusting for sociodemographic factors and happiness with school. Hyperactivity predicted poor academic attainment for males (OR 2.17, 95% CI 1.13 to 4.19) and females (OR 2.85, 95% CI 1.24 to 6.03) after controlling for the effects of all explanatory variables.

Table 3: Sex differences in odds ratios for low attainment at Key Stage 4 by total mental health difficulties and domain scores, adjusted for explanatory factors.

		Unadjusted		Fully adjusted	
Emotional	Male	3.07**	[1.48,6.38]	2.36	[0.83,6.64]
	Female	1.49	[0.91,2.43]	0.73	[0.34,1.57]
Peer	Male	2.36**	[1.39,4.02]	1.79	[0.83,3.84]
	Female	2.55**	[1.45,4.48]	0.99	[0.41,2.40]
Conduct	Male	1.65*	[1.03,2.66]	0.93	[0.42,2.05]
	Female	2.17**	[1.22,3.86]	1.29	[0.52,3.18]
Hyperactivity	Male	2.35***	[1.49,3.71]	2.17*	[1.11,4.23]
	Female	2.63***	[1.59,4.35]	2.85**	[1.30,6.23]
Total score	Male	3.16***	[1.79,5.60]	2.77*	[1.24,6.16]
	Female	3.36***	[1.97,5.71]	1.69	[0.72,3.95]

Note: Imputed model, Males N=550; Females N=560
Unadjusted: unadjusted odds of mental difficulties

For peer review only - http://bmjopen.bmj.com/site/about/guidelines.xhtml

Fully adjusted: odds of mental difficulties controlling for age, ethnicity, prior attainment at KS2, household social class, maternal education, household poverty, family composition, happy with school work, happy with school, parental interest in school, parents attend parent evening, family support, quarrels with parents, parental mental and physical health. *** $p<0.001$, ** $p<0.01$, * $p<0.05$

Results for the stepwise adjustment towards the full model are found in supplementary table A

For peer review only

DISCUSSION

This longitudinal sample of adolescents observed a strong association between mental health difficulties between the ages of 11 and 14 and later educational attainment at age 16. After accounting for the confounding effects of a range of socioeconomic, school-based and parenting factors known to predict lower attainment, young people with poor mental health were twice as likely to not reach the educational benchmark in England.

It is noteworthy that although prior attainment and family socioeconomic circumstances are well-established predictors of later performance at school (34) they did not explain the independent association between poor mental health difficulties and later attainment. This suggests that the impact of poor mental health in previous high achievers is likely to be as great as it is in those with previously low levels of attainment. In the same way, poor mental health is associated with educational performance to the same extent in young people from more advantaged social backgrounds as it is in those from poorer backgrounds. This implies that improving mental health in early adolescence may be an effective, indirect mechanism for narrowing the socioeconomic gap in attainment. Although the association between poorer mental health and lower attainment operates regardless of socioeconomic background, interventions to improve mental health will disproportionately involve those from disadvantaged backgrounds as they are more likely to experience mental health difficulties, potentially increasing average attainment levels within this group to a greater extent than within the majority population who are not disadvantaged. The potential effect at a population level would be to reduce the average difference in attainment between socioeconomic groups, and narrow educational and consequent social inequalities.

The association between lower attainment and overall mental difficulties was largely driven by the presence of hyperactivity disorder which remained highly significant after accounting for other explanatory factors. The relationship between hyperactivity disorder and lower attainment is has been documented eslewhere (35). Our data support the on-going development of school-based early interventions targeted towards hyperactivity disorders (36) focussing on meeting the specific needs of children and young people to enable them to reach their academic potential.

All four domains of mental difficulties were significantly related to lower attainment for males and females in unadjusted models, apart from emotional disorder in females. In terms of overall mental difficulties, males and females were equally likely to not achieve the GCSE benchmark, but the likelihood of not achieving the benchmark diminished for females after controlling for explanatory factors while this relationship remained significant for males. This is concurrent with previous work on the same sample assessing educational

For peer review only - http://bmjopen.bmj.com/site/about/guidelines.xhtml

attainment at age 18,(37) which controlled for similar explanatory factors. However, in contrast to our findings, females at age 18 exhibited a weaker relationship between mental difficulties and attainment than males even though they were significantly more likely to experience poor mental health - females being more likely to be conscientious high achievers was suggested as a possible explanation. Although the reason for this difference needs further investigation, these findings confirm important age and sex differences which ought to be accounted for when devising interventions aimed at promoting adolescent mental health.

Limitations

Consent to data linkage and successful linkage between the UKHLS and the NPD was predicted by ethnicity, household structure and social class. The inclusion of these variables in the imputation and the final models may mitigate against some of these selection effects, the lack of an analytic weight and the ethical limitation of being unable to impute missing data for sensitive information which has been actively protected by the respondent means that data is unlikely to be representative; prevalence estimates should be interpreted cautiously and may not be generalisable to the English population. This does not, however, diminish confidence in the associations identified by the prospective approach taken, bolstered through adjusting for prior attainment within explanatory models. Although the collection of mental difficulties data from young people is preferable than from their parents, this information was self-reported rather than a clinical diagnosis. Other measures of wellbeing and mental health ought to be considered in future analysis as associations with different constructs may differ from those presented here. Lastly, mediation analysis has not been conducted in this study though predictors of attainment such as happiness with school may be candidate variables. Caution should be applied to interpreting these candidate mediators as current estimates of the effect of mental difficulties on attainment may be considered overadjusted.

For peer review only

Contributions

NS and LM designed the analysis which was carried out by LM and MA under guidance from MS and NS. AH and SS contributed to the study design and drafting of the manuscript.

Declarations of interest

None

Funding. The project was funded by the Economic and Social Research Council Secondary Data Analysis Initiative (ES/R005400/1 to NS).

Competing interests: None declared.

Patient consent: Obtained.

Patient and public involvement:
It was not appropriate or possible to involve patients or the public in the design, or conduct, or reporting, or dissemination plans of our research.

Provenance and peer review: Not commissioned; externally peer reviewed.

Data Sharing Statement: Data are available in a public, open access repository. All data are hosted by the UK Data Service (UKDS):

National Pupil Database data is available under secure access licence agreement to registered and approved researchers.
10.5255/UKDA-SN-7642-2

Understanding Society Main Survey Data are available to registered users under standard terms of the UKDS End User Licence Agreement.
http://doi.org/10.5255/UKDA-SN-6614-13.

Ethics Statement: The data used are publicly available via UK Data Service repository (study numbers 6614 and 8644), and do not require ethical assessment for academic research purposes. The University of Essex Ethics Committee approved the survey data collection. No ethics approval number was produced. Ethics approval for data collection was granted by letter dated 6 July 2007 for Waves 1 and 2 and by letter dated 17 December 2010 for Waves 3 to 5

https://www.understandingsociety.ac.uk/documentation/mainstage/user-guides/main-survey-user-guide/ethics

Figure 1: Flow chart describing the breakdown of the combined Wave 1 and Wave 3 study population of the UKHLS into the analytic sample

For peer review only - http://bmjopen.bmj.com/site/about/guidelines.xhtml

1. NHS Digital. Mental Health of Children and Young People in England, 2017. [Internet]. 2018. Available from: https://digital.nhs.uk/data-and-information/publications/statistical/mental-health-of-children-and-young-people-in-england/2017/2017

2. Department of Health. Future in mind - Promoting, protecting and improving our children and young people's mental health and wellbeing [Internet]. 2015 p. 81–81. Available from: https://assets.publishing.service.gov.uk/government/uploads/system/uploads/attachment_data/file/414024/Childrens_Mental_Health.pdf

3. Chowdry H, McBride T. Disadvantage, Behaviour And Cognitive Outcomes: Longitudinal Analysis From Age 5 To 16. London; 2017.

4. Crawford C, Greaves E. Socio-economic, ethnic and gender differences in HE participation. [Internet]. Department for Business, Innovation and Skills; 2015 p. 116–116. Available from: https://assets.publishing.service.gov.uk/government/uploads/system/uploads/attachment_data/file/474273/BIS-15-85-socio-economic-ethnic-and-gender-differences.pdf

5. Lessof C, Ross A, Brind R. Multiple disadvantage and KS4 attainment: evidence from LSYPE2 [Internet]. GOV.UK. 2019 [cited 2020 Apr 7]. Available from: https://www.gov.uk/government/publications/multiple-disadvantage-and-ks4-attainment-evidence-from-lsype2

6. Schoon I. Planning for the Future: Changing Education Expectations in Three British Cohorts. Historical Social Research [Internet]. 2010;35(2). Available from: http://www.jstor.org/stable/20762452?seq=1#page_scan_tab_contents

7. Flouri E. Parental interest in children's education, children's self-esteem and locus of control, and later educational attainment: twenty-six year follow-up of the 1970 British Birth Cohort. The British journal of educational psychology. 2006;76(Pt 1):41–55.

8. Sellström E, Bremberg S. Is there a "school effect" on pupil outcomes? A review of multilevel studies. J Epidemiol Community Health. 2006 Feb;60(2):149–55.

9. Hale DR, Viner RM. How adolescent health influences education and employment: investigating longitudinal associations and mechanisms. J Epidemiol Community Health. 2018 Jun 1;72(6):465–70.

10. Dalsgaard S, McGrath J, Østergaard SD, Wray NR, Pedersen CB, Mortensen PB, et al. Association of Mental Disorder in Childhood and Adolescence With Subsequent Educational Achievement. JAMA Psychiatry. 2020 Aug 1;77(8):797–805.

11. Kessler C, Saunders B, Ph D, Foster L, Stang PE, Al E. Social Consequences of Psychiatric Disorders, I: Educational Attainment. American Journal of Psychiatry. 1995;15(July):1026–32.

For peer review only

12. Veldman K, Bültmann U, Stewart RE, Ormel J, Verhulst FC, Reijneveld SA. Mental Health Problems and Educational Attainment in Adolescence: 9-Year Follow-Up of the TRAILS Study. PLOS ONE. 2014 Jul 21;9(7):e101751.

13. L Marshall RW A Dunatchik, NR Smith. Supporting Mental Health in Schools and Colleges. https://www.gov.uk/government/publications/supporting-mental-health-in-schools-and-colleges. 2017;(ISBN 978-1-78105-757-5, DFE-RR697b).

14. Rothon C, Head J, Clark C, Klineberg E, Cattell V, Stansfeld S. The impact of psychological distress on the educational achievement of adolescents at the end of compulsory education. Social Psychiatry and Psychiatric Epidemiology. 2009 May 1;44(5):421–7.

15. Deighton J, Humphrey N, Belsky J, Boehnke J, Vostanis P, Patalay P. Longitudinal pathways between mental health difficulties and academic performance during middle childhood and early adolescence. The British journal of developmental psychology. 2018 Mar 1;36(1):110–26.

16. Clayborne ZM, Varin M, Colman I. Systematic Review and Meta-Analysis: Adolescent Depression and Long-Term Psychosocial Outcomes. J Am Acad Child Adolesc Psychiatry. 2019 Jan;58(1):72–9.

17. Sellers R, Warne N, Pickles A, Maughan B, Thapar A, Collishaw S. Cross-cohort change in adolescent outcomes for children with mental health problems. Journal of Child Psychology and Psychiatry. 2019 Jul 31;60(7):813–21.

18. Marshall L, Wishart R, Dunatchik A, Smith N. Supporting Mental Health in Schools and Colleges [Internet]. 2017 [cited 2020 Jul 4]. Available from: https://assets.publishing.service.gov.uk/government/uploads/system/uploads/attachment_data/file/634726/Supporting_Mental-Health_survey_report.pdf

19. University of Essex, Institute for Social and Economic Research, NatCen Social Research, Kantar Public. Understanding Society: Waves 1-9, 2009-2018 and Harmonised BHPS: Waves 1-18, 1991-2009. [data collection]. 12th Edition. UK Data Service. 2019. 2019.

20. Institute for Social and Economic Research. Understanding Society: The UK Household Longitudinal Study, Waves 1-9 - User Guide [Internet]. University of Essex; 2019 [cited 2020 Sep 4]. Available from: https://www.understandingsociety.ac.uk/sites/default/files/downloads/documentation/mainstage/user-guides/mainstage-user-guide.pdf

21. Jay MA, Grath-Lone LM, Gilbert R. Data Resource: the National Pupil Database (NPD). International Journal of Population Data Science [Internet]. 2019 Mar 20 [cited 2020 Apr 9];4(1). Available from: https://ijpds.org/article/view/1101

22. Knies G, Burton J. Analysis of four studies in a comparative framework reveals: health linkage consent rates on British cohort studies higher than

For peer review only - http://bmjopen.bmj.com/site/about/guidelines.xhtml

on UK household panel surveys. BMC medical research methodology. 2014 Nov;14:125–125.

23. Department for Education. GCSE and equivalent results: 2011 to 2012 (revised) [Internet]. 2013 Jan [cited 2020 Apr 14]. Available from: https://www.gov.uk/government/statistics/revised-gcse-and-equivalent-results-in-england-academic-year-2011-to-2012

24. Goodman R. The Strengths and Difficulties Questionnaire: a research note. Journal of Child Psychology and Psychiatry. 1997;38(5):581–6.

25. Biederman J, Monuteaux MC, Doyle AE, Seidman LJ, Wilens TE, Ferrero F, et al. Impact of Executive Function Deficits and Attention-Deficit/Hyperactivity Disorder (ADHD) on Academic Outcomes in Children. Journal of Consulting and Clinical Psychology. 2004 Oct;72(5):757–66.

26. Woodward LJ, Fergusson DM. Childhood peer relationship problems and later risks of educational under-achievement and unemployment. Journal of child psychology and psychiatry, and allied disciplines. 2000 Feb;41(2):191–201.

27. Deighton J, Lereya ST, Casey P, Patalay P, Humphrey N, Wolpert M. Prevalence of mental health problems in schools: poverty and other risk factors among 28 000 adolescents in England. British Journal of Psychiatry. 2019;215(3):565–7.

28. Strand S. The limits of social class in explaining ethnic gaps in educational attainment. British Educational Research Journal. 2011;37(2):197–229.

29. Goodman A, Gregg P. Poorer children’s educational attainment: how important are attitudes and behaviour? [Internet]. JRF. 2010 [cited 2020 Apr 7]. Available from: https://www.jrf.org.uk/report/poorer-children%E2%80%99s-educational-attainment-how-important-are-attitudes-and-behaviour

30. Carneiro P, Meghir C, Parey M. Maternal Education, Home Environments, and the Development of Children and Adolescents. Journal of the European Economic Association. 2013 Jan 1;11(suppl_1):123–60.

31. Knies G. Income effects on children’s life satisfaction: Longitudinal evidence for England [Internet]. ISER Working Paper Series; 2017 [cited 2020 Apr 17]. Report No.: 2017–02. Available from: https://www.econstor.eu/handle/10419/163545

32. Ware Jr JE, Kosinski M, Keller SD. A 12-Item Short-Form Health Survey: construction of scales and preliminary tests of reliability and validity. Medical care. 1996;34(3):220–33.

33. Suldo SM, Riley KN, Shaffer EJ. Academic Correlates of Children and Adolescents’ Life Satisfaction. School Psychology International. 2006 Dec 1;27(5):567–82.

For peer review only

34. Benton T, Such, T. Analysis of use of Key Stage 2 data in GCSE predictions [Internet]. OFQUAL; 2014 [cited 2020 Jul 8]. Available from: https://assets.publishing.service.gov.uk/government/uploads/system/uploads/attachment_data/file/429074/2014-06-16-analysis-of-use-of-key-stage-2-data-in-gcse-predictions.pdf

35. Birchwood J, Daley D. Brief report: The impact of Attention Deficit Hyperactivity Disorder (ADHD) symptoms on academic performance in an adolescent community sample. Journal of Adolescence. 2012 Feb 1;35(1):225–31.

36. May F, Ford T, Janssens A, Newlove-Delgado T, Russell AE, Salim J, et al. Attainment, attendance, and school difficulties in UK primary schoolchildren with probable ADHD. British Journal of Educational Psychology. 2021;91(1):442–62.

37. Tregaskis O, Nandi A, Watson D. An examination of the relationship between adolescent mental health and educational outcomes in early adulthood: Secondary analysis of Understanding Society data wave 1 to wave 8. 2020.

For peer review only

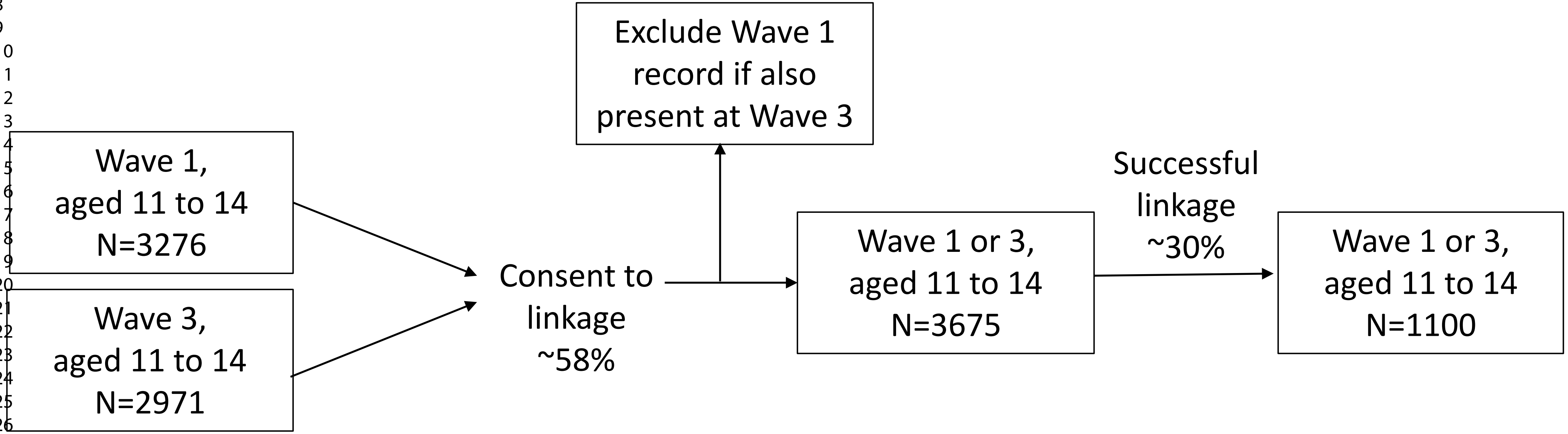
Exclude Wave 1 record if also present at Wave 3
Wave 1, aged 11 to 14 N=3276
Wave 3, aged 11 to 14 N=2971
Consent to linkage ~58%
Wave 1 or 3, aged 11 to 14 N=3675
Successful linkage ~30%
Wave 1 or 3, aged 11 to 14 N=1100

Supplementary table A: Odds ratios for low attainment at Key Stage 4 by total mental health difficulties and domain scores, adjusted stepwise for explanatory factors, by sex.

	Emotional		Peer		Conduct		Hyperactivity		Total score	
	Males	Females	Males	Females	Males	Females	Males	Females	Males	Females
Model 1	**3.07****<br>**[1.48,6.38]**	1.49<br>[0.91,2.43]	**2.36****<br>**[1.39,4.02]**	**2.55****<br>**[1.45,4.48]**	**1.65***<br>**[1.03,2.66]**	**2.17****<br>**[1.22,3.86]**	**2.35*****<br>**[1.49,3.71]**	**2.63*****<br>**[1.59,4.35]**	**3.16*****<br>**[1.79,5.60]**	**3.36*****<br>**[1.97,5.71]**
Model 2	**3.22****<br>**[1.55,6.71]**	1.54<br>[0.95,2.50]	**2.43****<br>**[1.43,4.14]**	**2.78*****<br>**[1.59,4.86]**	**1.71***<br>**[1.06,2.76]**	**2.15***<br>**[1.18,3.89]**	**2.40*****<br>**[1.51,3.79]**	**2.61*****<br>**[1.56,4.37]**	**3.30*****<br>**[1.85,5.87]**	**3.47*****<br>**[2.03,5.92]**
Model 3	**2.91***<br>**[1.17,7.22]**	1.41<br>[0.77,2.57]	**2.34***<br>**[1.20,4.56]**	1.31<br>[0.61,2.79]	1.56<br>[0.86,2.85]	**2.67****<br>**[1.35,5.26]**	**2.40****<br>**[1.36,4.24]**	**3.45*****<br>**[1.90,6.27]**	**3.39*****<br>**[1.74,6.62]**	**3.91*****<br>**[2.04,7.51]**
Model 4	**2.89***<br>**[1.05,7.92]**	1.23<br>[0.63,2.42]	**2.23***<br>**[1.07,4.63]**	1.26<br>[0.55,2.90]	1.44<br>[0.76,2.72]	**2.42***<br>**[1.16,5.05]**	**2.68****<br>**[1.43,5.04]**	**3.43*****<br>**[1.75,6.73]**	**3.38****<br>**[1.64,6.98]**	**3.52*****<br>**[1.69,7.32]**
Model 5	2.37<br>[0.85,6.59]	0.98<br>[0.49,1.97]	1.90<br>[0.93,3.87]	1.12<br>[0.48,2.60]	1.03<br>[0.50,2.12]	1.82<br>[0.85,3.93]	**2.23***<br>**[1.15,4.31]**	**2.64****<br>**[1.35,5.18]**	**2.66***<br>**[1.25,5.70]**	**2.61***<br>**[1.22,5.57]**
Model 6	2.51<br>[0.87,7.28]	0.76<br>[0.35,1.65]	1.85<br>[0.88,3.90]	1.09<br>[0.45,2.64]	0.98<br>[0.45,2.14]	1.35<br>[0.54,3.32]	**2.17***<br>**[1.13,4.19]**	**2.73***<br>**[1.24,6.03]**	**2.86****<br>**[1.30,6.29]**	1.79<br>[0.76,4.25]
Model 7	2.36<br>[0.83,6.64]	0.73<br>[0.34,1.57]	1.79<br>[0.83,3.84]	0.99<br>[0.41,2.40]	0.93<br>[0.42,2.05]	1.29<br>[0.52,3.18]	**2.17***<br>**[1.11,4.23]**	**2.85****<br>**[1.30,6.23]**	**2.77***<br>**[1.24,6.16]**	1.69<br>[0.72,3.95]

Note: Imputed model, Males N=550; Females N=560
Model 1: unadjusted odds of socioemotional difficulties; Model 2: adjusts for Model 1 + age, ethnicity; Model 3: adjusts for Model 2 + prior attainment at KS2; Model 4: adjusts for Model 3 + household social class, maternal education, household poverty, family composition Model 5: adjusts for Model 4 + happy with school work, happy with school; Model 6: adjusts Model 5 + parental interest in school, parents attend parent evening, family support, quarrels with parents; Model 7: adjusts for Model 6 +parental mental and physical health.
Significant odds ratios (95% confidence interval) shown in bold text.

For peer review only

For peer review only

STROBE 2007 (v4) Statement—Checklist of items that should be included in reports of *cohort studies*

Section/Topic	Item #	Recommendation	Reported on page #
**Title and abstract**	1	(*a*) Indicate the study's design with a commonly used term in the title or the abstract	2
		(*b*) Provide in the abstract an informative and balanced summary of what was done and what was found	2
**Introduction**			
Background/rationale	2	Explain the scientific background and rationale for the investigation being reported	4
Objectives	3	State specific objectives, including any prespecified hypotheses	5
**Methods**			
Study design	4	Present key elements of study design early in the paper	6
Setting	5	Describe the setting, locations, and relevant dates, including periods of recruitment, exposure, follow-up, and data collection	6
Participants	6	(*a*) Give the eligibility criteria, and the sources and methods of selection of participants. Describe methods of follow-up	6,7
		(*b*) For matched studies, give matching criteria and number of exposed and unexposed	n/a
Variables	7	Clearly define all outcomes, exposures, predictors, potential confounders, and effect modifiers. Give diagnostic criteria, if applicable	7,8
Data sources/ measurement	8*	For each variable of interest, give sources of data and details of methods of assessment (measurement). Describe comparability of assessment methods if there is more than one group	7,8
Bias	9	Describe any efforts to address potential sources of bias	6, 15
Study size	10	Explain how the study size was arrived at	6
Quantitative variables	11	Explain how quantitative variables were handled in the analyses. If applicable, describe which groupings were chosen and why	4 7,8
Statistical methods	12	(*a*) Describe all statistical methods, including those used to control for confounding	8
		(*b*) Describe any methods used to examine subgroups and interactions	8
		(*c*) Explain how missing data were addressed	8
		(*d*) If applicable, explain how loss to follow-up was addressed	6,8
		(*e*) Describe any sensitivity analyses	n/a
**Results**			

For peer review only

Participants	13*	(a) Report numbers of individuals at each stage of study—eg numbers potentially eligible, examined for eligibility, confirmed eligible, included in the study, completing follow-up, and analysed	6,9
		(b) Give reasons for non-participation at each stage	6
		(c) Consider use of a flow diagram	6
Descriptive data	14*	(a) Give characteristics of study participants (eg demographic, clinical, social) and information on exposures and potential confounders	9,10
		(b) Indicate number of participants with missing data for each variable of interest	9,10
		(c) Summarise follow-up time (eg, average and total amount)	n/a
Outcome data	15*	Report numbers of outcome events or summary measures over time	
Main results	16	(*a*) Give unadjusted estimates and, if applicable, confounder-adjusted estimates and their precision (eg, 95% confidence interval). Make clear which confounders were adjusted for and why they were included	11,12
		(*b*) Report category boundaries when continuous variables were categorized	7
		(*c*) If relevant, consider translating estimates of relative risk into absolute risk for a meaningful time period	n/a
Other analyses	17	Report other analyses done—eg analyses of subgroups and interactions, and sensitivity analyses	12
**Discussion**			
Key results	18	Summarise key results with reference to study objectives	14
**Limitations**			
Interpretation	20	Give a cautious overall interpretation of results considering objectives, limitations, multiplicity of analyses, results from similar studies, and other relevant evidence	14,15
Generalisability	21	Discuss the generalisability (external validity) of the study results	15
**Other information**			
Funding	22	Give the source of funding and the role of the funders for the present study and, if applicable, for the original study on which the present article is based	15,16

*Give information separately for cases and controls in case-control studies and, if applicable, for exposed and unexposed groups in cohort and cross-sectional studies.

Note: An Explanation and Elaboration article discusses each checklist item and gives methodological background and published examples of transparent reporting. The STROBE checklist is best used in conjunction with this article (freely available on the Web sites of PLoS Medicine at http://www.plosmedicine.org/, Annals of Internal Medicine at http://www.annals.org/, and Epidemiology at http://www.epidem.com/). Information on the STROBE Initiative is available at www.strobe-statement.org.

For peer review only

Adolescent mental health difficulties and educational attainment: findings from the UK Household Longitudinal Study

Journal:	*BMJ Open*
Manuscript ID	bmjopen-2020-046792.R2
Article Type:	Original research
Date Submitted by the Author:	12-Jun-2021
Complete List of Authors:	SMITH, NEIL; NatCen Social Research, Policy Research Centre<br>Marshall, Lydia; NatCen Social Research, Policy Research Centre<br>Albakri, Muslihah; NatCen Social Research, Policy Research Centre<br>Smuk, Melanie; London School of Hygiene & Tropical Medicine<br>Hagell, Ann; Association for Young People's Health<br>Stansfeld, Stephen; Barts and the London, Queen Marys School of Medicine and Dentistry, Psychiatry
**Primary Subject Heading**:	Epidemiology
Secondary Subject Heading:	Mental health
Keywords:	MENTAL HEALTH, PUBLIC HEALTH, Child & adolescent psychiatry < PSYCHIATRY, EPIDEMIOLOGY

SCHOLARONE™
Manuscripts

I, the Submitting Author has the right to grant and does grant on behalf of all authors of the Work (as defined in the below author licence), an exclusive licence and/or a non-exclusive licence for contributions from authors who are: i) UK Crown employees; ii) where BMJ has agreed a CC-BY licence shall apply, and/or iii) in accordance with the terms applicable for US Federal Government officers or employees acting as part of their official duties; on a worldwide, perpetual, irrevocable, royalty-free basis to BMJ Publishing Group Ltd ("BMJ") its licensees and where the relevant Journal is co-owned by BMJ to the co-owners of the Journal, to publish the Work in this journal and any other BMJ products and to exploit all rights, as set out in our licence.

The Submitting Author accepts and understands that any supply made under these terms is made by BMJ to the Submitting Author unless you are acting as an employee on behalf of your employer or a postgraduate student of an affiliated institution which is paying any applicable article publishing charge ("APC") for Open Access articles. Where the Submitting Author wishes to make the Work available on an Open Access basis (and intends to pay the relevant APC), the terms of reuse of such Open Access shall be governed by a Creative Commons licence – details of these licences and which Creative Commons licence will apply to this Work are set out in our licence referred to above.

Other than as permitted in any relevant BMJ Author's Self Archiving Policies, I confirm this Work has not been accepted for publication elsewhere, is not being considered for publication elsewhere and does not duplicate material already published. I confirm all authors consent to publication of this Work and authorise the granting of this licence.

For peer review only

Adolescent mental health difficulties and educational attainment: findings from the UK Household Longitudinal Study

Corresponding author:
Neil R Smith, PhD
NatCen Social Research
Policy Research Centre
35 Northampton Square
London
EC1V 0AX, UK
Neil.Smith@natcen.ac.uk

Lydia Marshall, PhD
NatCen Social Research
Policy Research Centre
35 Northampton Square
London
EC1V 0AX, UK
Lydia.Marshall@natcen.ac.uk

Muslihah Albakri
NatCen Social Research
Policy Research Centre
35 Northampton Square
London
EC1V 0AX, UK
Muslihah.Albakri@natcen.ac.uk

Melanie Smuk, PhD
London School of Hygiene & Tropical Medicine
Keppel Street
London
WC1E 7HT, UK
Melanie.Smuk@lshtm.ac.uk

Ann Hagell, PhD
Association for Young People's Health
7-14 Great Dover Street
London
SE1 4YR, UK
Ann@youngpeopleshealth.org.uk

Stephen Stansfeld, PhD
Centre for Psychiatry
Bart's and the London School of Medicine and Dentistry
London
EC1M 6BQ, UK
S.A.Stansfeld@qmul.ac.uk

3076 words

Keywords: mental health, adolescence, education, social epidemiology, life course

For peer review only

ABSTRACT

Objective: This study examines whether there is an independent association between mental difficulties in adolescence and educational attainment at age 16.

Design: Longitudinal study.

Setting: Nationally representative data from the UK Household Longitudinal Study (UKHLS) were linked to the National Pupil Database for England.

Participants: Respondents (N=1,100) to the UKHLS between 2009-2012 were linked to the National Pupil Database to investigate longitudinal associations between mental difficulties at ages 11-14 and educational attainment at age 16 (GCSE).

Primary outcome measure: Not gaining five or more GCSE qualifications at age 16, including English and maths at grade A*-C.

Results: An atypical total mental health difficulties score measured using the Strengths and Difficulties Questionnaire at ages 11-14 predicted low levels of educational attainment at age 16 (OR: 3.11 (95% CI: [2.11, 4.57]). Controlling for prior attainment and family sociodemographic factors, happiness with school(/work) and parental health, school engagement and relationship with the child partially attenuated the association which was significant in the fully adjusted model (2.05, 95% CI: [1.15,3.68]). The association was maintained in the fully adjusted model for males only (OR: 2.77 (95% CI: [1.24, 6.16]) but not for females. Hyperactivity disorder strongly predicted lower attainment for males (OR: 2.17 (95% CI: [1.11, 4.23]) and females (OR: 2.85 (95% CI: [1.30, 6.23]).

Conclusion: Mental difficulties at ages 11-14 were independently linked to educational success at age 16, highlighting an important pathway through which health in adolescence can determine young people's life chances.

For peer review only

For peer review only - http://bmjopen.bmj.com/site/about/guidelines.xhtml

STRENGTHS AND LIMITATIONS OF THIS STUDY.

- This is a large, nationally representative longitudinal cohort study containing self-assessed measures of mental health among young people linked to a National Pupil Database of educational records.
- The study captures a diverse range of social, demographic, economic and behavioural factors affecting young people in their home and school environment, permitting statistical adjustment for multiple confounding relationships which might explain the association between mental health and educational attainment.
- Consent to data linkage between the longitudinal study and the National Pupil Database was incomplete, though factors which predicted patterns of non-consent were controlled for within our models.
- Missing data was accounted for using multiple imputation methods which exploited the wide range of associations within the observed data to minimise errors within estimates of effect.

For peer review only

INTRODUCTION

Growing evidence of the prevalence of poor child and adolescent mental health has led to this issue becoming a key policy priority in the UK. Mental ill-health in children and young people in England increases age with around 14.4% of 11-16 years experiencing a mental disorder compared to 5.5% in their pre-school counterparts aged 2-4 years.(1) With 75% of adult mental health problems (excluding dementia) starting by the age of 18,(2) adolescence is a key period in the development of long-lasting mental health difficulties. The UK government's *Future in Mind* report (2) presented an important economic case for investment in early prevention of mental ill-health to mitigate against the costs of longer-term support for health needs. However, this argument neglects the impact that early life mental health potentially has on other early life outcomes fundamental in determining life chances, such as educational attainment.(3) Educational outcomes are closely associated with later-life chances with well-established links to employment, income, housing and offending as well as physical health and on-going mental health disorders. If poor mental health diminishes the capacity for individuals to fulfil their academic potential, mental health itself is likely to be a driver of educational inequality and consequent on-going social inequality.

On the other hand, the association between mental health and educational outcomes might not be direct, but rather incorporate the influence of confounding factors. A range of demographic and socioeconomic factors, such as gender, ethnicity, socioeconomic disadvantage and maternal education and parental health(4–6) have known relationships with educational attainment and must be accounted for when assessing the impact of poor mental health. Similarly, the home environment and specifically parental interest in schooling has been associated with higher attainment,(7) as have positive environmental "school effects", (8) whereas lower attainment has been associated with absence from school (1) or poor classroom behaviours. (9) What is less clear is the extent to which differential exposure to these factors also underpin disparities in mental health, and whether resulting differences in mental health might influence differences in attainment.

International research has demonstrated numerous associations between mental health and educational attainment.(10–12). The evidence base for England is less well-established which is of particular relevance during a time of policy interest in boosting mental health provision in schools.(13) There is some evidence of longitudinal associations between psychological distress in early adolescence and achievement at GCSE in England.(14,15) Similarly, poor mental health between ages 13 and 15 has been shown to be associated with low GCSE attainment and later unemployment, (9) demonstrating how the effects of poor early life mental health can extend into adulthood (16). Though many of these findings support the association between mental health and educational outcomes, they are often of low generalisability being based on

For peer review only - http://bmjopen.bmj.com/site/about/guidelines.xhtml

regional data or non-probability samples (14) or unable to account for a range of potentially explanatory factors. (15) There appears to be a strengthening of the relationship between adolescent mental health and educational outcomes in recent generations (17) so there is a pressing need for an up-to-date examination of nationally representative data for England.

Therefore, this study uses a novel and contemporary data linkage between the nationally representative UK Household Longitudinal Study linked to objectively measured official education records, to test associations between poor mental health and poor educational attainment. The study is significant in estimating the extent to which mental health in early adolescence has an independent association with attainment at age 16 in England in males and females. Robust evidence of a causal relationship between poor mental health and lower academic attainment could be crucial in inspiring investment in researching “what works” in supporting children and adolescents’ mental health. Although schools already appreciate the importance of supporting pupils’ health and wellbeing, (18) a proven link to academic outcomes could also encourage education and public health policymakers to invest more in mental health.

For peer review only - http://bmjopen.bmj.com/site/about/guidelines.xhtml

METHODS

UK Household Longitudinal Study (UKHLS)

The UKHLS is a nationally representative household panel survey (19) which began in 2009, aiming to understand social and economic change in Britain at the household and individual levels. Each wave of the survey collects information on approximately 100,000 individuals from 40,000 households, with adult household residents (aged 16 and over) responding using computer-assisted interview and self-completion questionnaire. Young people aged between 10-15 were offered a self-completion questionnaire. Further detail on the sampling design and data collection is available. (20) National educational records from the from the National Pupil Database (NPD) (21) for school-age children between ages 3 and 18 were linked to the UKHLS if parents and their children were living in England and consented to linkage at wave 1. Linkage consent rates did not differ systematically by parental class, or parental education though they were lower within ethnic minority groups which is consistent with other cohort studies. (22)

This analysis used a nationally representative sample of 11 to 14 year olds present at wave 1 (2009-2011) and wave 3 (2011-2013) linked to the NPD. Wave 2 (2010-2012) was excluded as it did not ask for information about mental health. Where respondents were present at both waves, data from wave 3 was selected as the respondent was further into adolescence. Figure 1 tracks the study population down to the final analytic sample.

The final sample consisted of all consenting youth panel respondents aged 11 to 14 years with data on mental health in wave 1 or wave 3 of UKHLS as well as NPD data on GCSE scores at ages 15 or 16 years (N=1110). The analytic sample covers England only due to the limited geographical coverage of the NPD.

Educational attainment

The primary outcome was a binary variable indicating low educational attainment, defined as whether the young person did not achieve 5 or more grades A*-C for the General Certificate of Secondary Education (GCSE), including English and maths. This was the benchmark measure of educational attainment at Key Stage 4 (KS4) at secondary schools in England during the study period. (23)

Mental difficulties

Young people completed the Strengths and Difficulties questionnaire (SDQ) validated for ages 4-15 years. (24) The SDQ asks questions about four domains of negative behaviours which have varying strengths of association with educational attainment, namely: conduct problems (11); hyperactivity (25); emotional symptoms (14); peer problems. (26) Scores from the four subscales were summed to construct a total difficulties score, where a higher score refers to a greater level of mental difficulties. Binary measures of mental difficulties were

For peer review only - http://bmjopen.bmj.com/site/about/guidelines.xhtml

derived based on developer guidance. (24) An “atypical” level of total difficulties was derived from the top 10% of the population scores (>=18 out of 40) and individual SDQ domains used validated “atypical” cut points which have also been used in a recent prevalence survey in England. (27)

Explanatory variables

We focussed on risk factors where the literature has established potentially causal associations with educational attainment and mental health respectively. All analyses were controlled for gender, age, ethnic group as well as the three tiered classification household’s highest parental occupational class, household deprivation and mother’s highest educational qualifications. (28–30) Parents’ highest current or previous occupational class was based on the National Statistics Socioeconomic Classification (NS-SEC) which was collapsed into a three-tier hierarchical scale (professional/managerial; intermediate; manual/routine) (31) with an additional category for overseas or no qualifications. The mother’s highest qualification was summarised on a three-tier hierarchical scale (degree or higher; A-level or equivalent; GCSE or equivalent with a separate category for none or other. Household poverty was derived based on income poverty, material poverty, subjective poverty and the receipt of benefits and was categorised into ‘not at all deprived’, ‘somewhat deprived’ or ‘highly deprived’.(32) Additionally, family type was grouped into two parent households, lone parent household or other family types.(28)

Parental relationships were assessed using a binary measures of young people’s self-reports on how interested their parent(s) were at how they did at school, attendance at parents’ evenings, frequency of quarrelling with either parent(s) and how often they feel supported by their family. (7) Parental physical and mental health was assessed using the SF-12 Physical and Mental Component Summary respectively, (33) with a score from either parent in the lowest quintile representing poor physical health and a mental health score of >=45.6 representing poor mental health.

Young people reported levels of happiness specifically with school-work as well as with school generally on a 7-point scale with a score of 5 or greater indicating happiness. (34) Prior attainment was measured based on whether young people achieved the expected level 4 reading, writing and mathematics at Key Stage 2 (KS2) (ages 10-11 years).

All non-educational attainment measures were taken at the time adolescent mental health was assessed.

Statistical analysis

Complete data was available was available for age, sex, ethnicity and family composition. Missing data was most common for household poverty (13%) so data was imputed under the missing at random assumption as poverty was associated with poorer explanatory outcomes, specifically lower level of

For peer review only - http://bmjopen.bmj.com/site/about/guidelines.xhtml

occupational class, maternal education, family composition and prior attainment. Given the overall low level of missingness, twenty imputed datasets were created. All explanatory variables and measures of mental difficulties shown in Table 1 were used in the imputation and missing data for explanatory variables (ranging between 1% and 13%) and mental difficulties (0.2%) was imputed. Data on GCSE grades were not imputed due to a high proportion of missing data (70%) due to a lack of linkage consent, and for ethical reasons given these individuals had not consented to their data being used for research.

The prevalence of low attainment and mental difficulties are described separately according to a range of selected socioeconomic, demographic and parent-related factors. Data was weighted using the cross-sectional self-completion weights in the UKHLS youth panel in wave 1 and wave 3.

Logistic regression was used to estimate separately the odds ratio of not achieving 5 A*-C GCSE grades including English and mathematics and of being classed as having mental difficulties. Stepwise regression models adjusted the odds ratios of having total mental difficulties and difficulties within each domain to examine the relative impact of prior attainment, sociodemographic factors, parent-child relationships, young person’s happiness with school and parental health on educational attainment. Models were stratified to explore gender differences in total and domain specific mental difficulties. All analyses were performed in Stata v16.1 (StataCorp, College Station, TX, USA).

For peer review only

RESULTS

The analytic sample was evenly split by gender and the overwhelming majority were aged 13 or 14 years old. Respondents tended to be from relatively socioeconomically advantaged backgrounds. A third of mothers were degree educated and 41.8% of households belonged to the highest social class. Over three-quarters of the sample reported high parental engagement with school and happiness with school-work. Prior attainment levels were positive for reading (93.3%), writing (82.6%) and maths (71.5%).

The proportion of young people not achieving the Key Stage 4 (KS4) benchmark of 5 GCSEs A*-C including English and maths varied by selected characteristics (Table 2). Low prior attainment at Key Stage 2 (KS2) was most strongly associated with not reaching the educational benchmark at KS4. Low attainment at KS4 was also associated with lower social class, lower maternal education, higher household poverty scores and poorer parent-child relationships as well as poor parental mental and physical health. Reported unhappiness with school and school-work, and lower parental involvement in schooling was also significantly associated with low attainment.

Table 1: Prevalence % of low educational attainment at Key Stage 4 by sociodemographic and parental characteristics.

	% (N)	Low attainment %	Odds ratio	95% CI
Sex				
Male	51.6 (550)	42.0	1	Ref
Female	48.4 (560)	31.5***	0.64***	[0.49,0.83]
Age (years)				
11	1.1 (14)	65.5*	3.42*	[1.05,11.15]
12	9.7 (111)	38.4	1.12	[0.72,1.76]
13	38.9 (432)	37.3	1.07	[0.81,1.42]
14	50.4 (553)	35.7	1.00	Ref
Ethnic group				
White British	86.1 (839)	36.9	1	Ref
Other ethnic group	13.9 (271)	37.0	1.00	[0.72,1.40]
Parental highest social class (NS-SEC)				
Management & professional	41.8 (439)	23.4	1.00	Ref
Intermediate	22.7 (253)	34.2**	1.70**	[1.19,2.44]
Routine & manual	31.0 (345)	53.6**	3.79***	[2.74,5.25]
Unemployed	4.4 (53)	61.3**	5.18***	[2.60,10.35]
Mother's highest qualification				
Degree or higher	33.2 (351)	24.0	1	Ref
A-level or equivalent	17.5 (185)	21.8	0.88	[0.57,1.38]

For peer review only - http://bmjopen.bmj.com/site/about/guidelines.xhtml

GCSE or equivalent	29.5 (309)	41.3***	2.23***	[1.57,3.19]
None/other	19.8 (239)	65.4***	6.00***	[4.06,8.86]
Household poverty score				
Not at all deprived	20.9 (179)	16.2	1	Ref
Somewhat deprived	54.0 (493)	35.8***	2.89***	[1.84,4.56]
Highly deprived	25.1 (266)	56.5***	6.74***	[4.08,11.13]
Family composition				
Two-parent	69.7 (759)	32.9	1	Ref
Single parent	27.8 (321)	47.6***	1.86***	[1.39,2.47]
Other	2.5 (30)	suppressed	-	-
Happy with school-work				
Happy	74.7 (840)	29.6	1	Ref
Not happy	25.3 (263)	58.6***	3.38***	[2.49,4.57]
Happy with school				
Happy	78.6 (876)	32.0	1	Ref
Not happy	21.4 (220)	54.7***	2.57***	[1.86,3.53]
Parental interest in school				
Always or nearly always	79.0 (871)	34.4	1	Ref
Sometimes or rarely	21.0 (220)	46.4**	1.66**	[1.20,2.28]
Regularly attends parents' evenings				
Always or nearly always	81.1 (896)	29.6	1	Ref
Sometimes or rarely	18.9 (199)	68.0***	5.05***	[3.56,7.16]
Feels supported by family				
Always or mostly	76.3 (837)	34.7	1	Ref
Not supported	23.7 (269)	44.1*	1.49*	[1.10,2.02]
Regularly quarrels with either parent				
Less than once a week	60.0 (662)	33.1	1	Ref
More than once a week	40.0 (423)	42.6**	1.50**	[1.14,1.97]
Parental mental health				
Not poor	56.8 (539)	30.0	1	Ref
Poor	43.2 (423)	46.0***	1.98***	[1.50,2.62]
Parental physical health				
Not poor	58.6 (564)	32.9	1	Ref
Poor	41.4 (402)	42.6**	1.52**	[1.15,2.00]
Attainment at Key Stage 2 Maths				
Achieved level 4	71.5 (860)	26.6	1	Ref
Did not achieve level 4	17.4 (169)	85.9***	16.92***	[10.65,26.87]
Attainment at Key Stage 2 Writing				
Achieved level 4	82.6 (270)	22.2	1	Ref
Did not achieve level 4	28.4 (759)	73.9***	9.96***	[7.14,13.90]
Attainment at Key Stage 2 Reading				
Achieved level 4	92.3 (947)	32.4	1	Ref
Did not achieve level 4	7.7 (74)	91.5***	22.65***	[9.85,52.09]

For peer review only - http://bmjopen.bmj.com/site/about/guidelines.xhtml

Notes: Ref=Reference group; Unweighted N; Imputed and weighted percentages shown; low educational attainment defined as < 5 GCSEs at A*-C including English and maths; some values are suppressed due to small base sizes and risk of disclosure; *** p<0.001, **p<0.01, *p<0.05

There was a similar patterning to the prevalence of mental difficulties (Table 2). Poorer household socioeconomic circumstances, parental engagement with school and health, parent-child relationships and the young person's happiness with school and school-work were all significantly associated with increased odds of being classified with mental difficulties. However, there was no significant difference in the prevalence of mental health difficulties by sex, and the association between prior attainment and current mental difficulties was relatively weak and significant only for writing at KS2.

Table 2: Prevalence % of mental difficulties by sociodemographic and parental characteristics.

	% (N)	SDQ score >=18 %	Odds ratio	95% CI
Sex				
Male	51.6 (550)	12.1	1	Ref
Female	48.4 (560)	15.0	1.28	[0.88,1.86]
Age (years)				
11	1.1 (14)	35.3*	3.79*	[1.11,12.93]
12	9.7 (111)	18.4	1.57	[0.86,2.86]
13	38.9 (432)	12.9	1.03	[0.68,1.55
14	50.4 (553)	12.6	1	Ref
Ethnic group				
White British	86.1 (839)	14.1	1	Ref
Other ethnic group	13.9 (271)	9.6	0.65	[0.37,1.11]
Parental highest social class (NS-SEC)				
Management & professional	41.8 (439)	9.0	1	Ref
Intermediate	22.7 (253)	14.0	1.64	[0.99,2.74]
Routine & manual	31.0 (345)	17.3**	2.11**	[1.34,3.33]
Unemployed	4.4 (53)	26.9**	3.71**	[1.56,8.84]
Mother's highest qualification				
Degree or higher	33.2 (351)	11.1	1	Ref
A-level or equivalent	17.5 (185)	11.1	1.00	[0.55,1.84]
GCSE or equivalent	29.5 (309)	13.3	1.23	[0.75,2.01]
None/other	19.8 (239)	20.0**	2.00**	[1.20,3.33]
Household poverty score				
Not at all deprived	20.9 (179)	8.0	1	Ref
Somewhat deprived	54.0 (493)	11.6	1.50	[0.78,2.88]
Highly deprived	25.1 (266)	22.1***	3.26***	[1.67,6.36]
Family composition				
Two-parent	69.7 (759)	12.0	1	Ref

For peer review only - http://bmjopen.bmj.com/site/about/guidelines.xhtml

For peer review only

Single parent	27.8 (321)	18.5*	1.66*	[1.12,2.47]
Other	2.5 (30)	suppressed	-	-
Happy with school-work				
Happy	74.7 (840)	9.0	1	Ref
Not happy	25.3 (263)	26.8***	3.71***	[2.52,5.47]
Happy with school				
Happy	78.6 (876)	9.3	1	Ref
Not happy	21.4 (220)	28.9***	3.96***	[2.66,5.90]
Parental interest in school				
Always or nearly always	79.0 (871)	10.6	1	Ref
Sometimes or rarely	21.0 (220)	24.4***	2.73***	[1.81,4.10]
Regularly attends parents' evenings				
Always or nearly always	81.1 (896)	10.8	1	Ref
Sometimes or rarely	18.9 (199)	24.9***	2.73***	[1.79,4.16]
Feels supported by family				
Always or mostly	76.3 (837)	9.0	1	Ref
Not supported	23.7 (269)	27.8***	3.87***	[2.62,5.71]
Regularly quarrels with either parent				
Less than once a week	60.0 (662)	7.5	1	Ref
More than once a week	40.0 (423)	22.5***	3.59***	[2.40,5.36]
Parental mental health				
Not poor	56.8 (539)	11.3	1	Ref
Poor	43.2 (423)	16.4*	1.55*	[1.02,2.36]
Parental physical health				
Not poor	58.6 (564)	11.3	1	Ref
Poor	41.4 (402)	16.6*	1.57*	[1.04,2.37]
Attainment at Key Stage 2 Maths				
Achieved level 4	71.5 (860)	12.5	1	Ref
Did not achieve level 4	17.4 (169)	18.2	1.56	[0.98,2.48]
Attainment at Key Stage 2 Writing				
Achieved level 4	82.6 (270)	11.5	1	Ref
Did not achieve level 4	28.4 (759)	18.4**	1.72**	[1.15,2.58]
Attainment at Key Stage 2 Reading				
Achieved level 4	92.3 (947)	13.4	1	Ref
Did not achieve level 4	7.7 (74)	15.1	1.15	[0.56,2.37]

Notes: Ref=Reference group; Unweighted N; Imputed and weighted percentages shown; some values are suppressed due to small base sizes and risk of disclosure; *** p<0.001, **p<0.01, *p<0.05

Young people classified with mental health difficulties were over three times more likely to not reach the KS4 GCSE benchmark (OR 3.11, 95% CI [2.11-4.57]) in the unadjusted model (Table 3). Incrementally controlling for prior attainment and

For peer review only

household socioeconomic factors did not attenuate this risk. Controlling for a young person's happiness with school and school-work (Model 5) and parental relationships and support (Model 6) partially diminished this risk. However, the fully adjusted model demonstrated that young people with poor mental health were over twice as likely (OR 2.05, 95% CI [1.15-3.68]) to not reach the educational benchmark than their counterparts with sub-clinical difficulties. Within individual sub-domains, the fully adjusted model could not account for the higher odds of not reaching the educational benchmark for those with hyperactivity disorder (OR 2.38, 95% CI [1.48-3.82]), implying that hyperactivity disorder largely drives the association between mental difficulties scores and lower attainment. For emotional and peer disorders, these risks were no longer significant once adjusted for prior attainment and sociodemographic factors, and conduct disorder no longer predicted lower attainment following adjustment for happiness with school and school-work.

Table 3: Odds ratios for low attainment at Key Stage 4 by total mental health difficulties and domain scores, adjusted stepwise for explanatory factors.

	Model 1	Model 2	Model 3	Model 4	Model 5	Model 6	Model 7
**Emotional**	1.64* [1.11,2.41]	1.88** [1.27,2.78]	1.75* [1.07,2.85]	1.55 [0.91,2.65]	1.22 [0.71,2.10]	1.12 [0.63,1.99]	1.07 [0.61,1.90]
**Peer**	2.44*** [1.66,3.58]	2.45*** [1.66,3.61]	1.67* [1.02,2.75]	1.50 [0.88,2.55]	1.31 [0.78,2.20]	1.26 [0.74,2.16]	1.20 [0.70,2.08]
**Conduct**	1.92*** [1.33,2.76]	1.83** [1.26,2.65]	1.91** [1.22,3.01]	1.65* [1.02,2.67]	1.25 [0.74,2.11]	1.10 [0.62,1.94]	1.07 [0.60,1.90]
**Hyperactivity**	2.52*** [1.80,3.52]	2.46*** [1.75,3.45]	2.77*** [1.84,4.18]	2.94*** [1.89,4.57]	2.39*** [1.52,3.78]	2.35*** [1.46,3.78]	2.38*** [1.48,3.82]
**Total score**	3.11*** [2.11,4.57]	3.25*** [2.20,4.80]	3.55*** [2.22,5.70]	3.20*** [1.90,5.37]	2.38** [1.38,4.12]	2.10* [1.17,3.77]	2.05* [1.15,3.68]

Note: Imputed model, N=1100
Model 1: unadjusted odds of low KS4 attainment
Model 2: adjusts for Model 1 + age, sex, ethnicity
Model 3: adjusts for Model 2 + prior attainment at KS2
Model 4: adjusts for Model 3 + household social class, maternal education, household poverty, family composition
Model 5: adjusts for Model 4 + happy with school work, happy with school

For peer review only

Model 6: adjusts Model 5 + parental interest in school, parents attend parent evening, family support, quarrels with parents
Model 7: adjusts for Model 6 +parental mental and physical health
*** p<0.001, **p<0.01, *p<0.05

Table 4 describes the sex-specific association between mental health difficulties and attainment to explore the well-established and significantly lower level of attainment in males than females observed in Table 1. There was an independent relationship between poor mental health and low attainment in males after controlling for all explanatory variables (OR 2.77, [1.30 to 6.29]). For females, the relationship between poor mental health and low attainment was no longer significant once prior attainment, sociodemographic factors and school enjoyment and parental support and engagement with school was controlled for.

For both sexes there were significant and generally strong associations between sub-domains of mental health and attainment. The single noteworthy exception was a lack of association with attainment in females with emotional disorder (OR 1.49, [0.91-2.43]). With exception to hyperactivity disorder, there were no significant associations with attainment in males and females after adjusting for sociodemographic factors and happiness with school. Hyperactivity disorder predicted poor academic attainment for males (OR 2.17, 95% CI 1.13 to 4.19) and females (OR 2.85, 95% CI 1.24 to 6.03) after controlling for the effects of all explanatory variables.

Table 4: Sex differences in odds ratios for low attainment at Key Stage 4 by total mental health difficulties and domain scores, adjusted for explanatory factors.

		Unadjusted		Fully adjusted	
Emotional	Male	3.07**	[1.48,6.38]	2.36	[0.83,6.64]
	Female	1.49	[0.91,2.43]	0.73	[0.34,1.57]
Peer	Male	2.36**	[1.39,4.02]	1.79	[0.83,3.84]
	Female	2.55**	[1.45,4.48]	0.99	[0.41,2.40]
Conduct	Male	1.65*	[1.03,2.66]	0.93	[0.42,2.05]
	Female	2.17**	[1.22,3.86]	1.29	[0.52,3.18]
Hyperactivity	Male	2.35***	[1.49,3.71]	2.17*	[1.11,4.23]
	Female	2.63***	[1.59,4.35]	2.85**	[1.30,6.23]
Total score	Male	3.16***	[1.79,5.60]	2.77*	[1.24,6.16]
	Female	3.36***	[1.97,5.71]	1.69	[0.72,3.95]

Note: Imputed model, Males N=550; Females N=560
Unadjusted: unadjusted odds of mental difficulties
Fully adjusted: odds of mental difficulties controlling for age, ethnicity, prior attainment at KS2, household social class, maternal education, household poverty, family composition, happy with school work, happy with school, parental interest in school, parents attend parent evening, family support,

For peer review only - http://bmjopen.bmj.com/site/about/guidelines.xhtml

quarrels with parents, parental mental and physical health. *** p<0.001, **p<0.01, *p<0.05

Results for the stepwise adjustment towards the full model are found in supplementary table A

For peer review only

DISCUSSION

This longitudinal sample of adolescents observed a strong association between mental health difficulties between the ages of 11 and 14 and later educational attainment at age 16. After accounting for the confounding effects of a range of socioeconomic, school-based and parenting factors known to predict lower attainment, young people with mental difficulties were twice as likely to not reach the educational benchmark in England.

The association between lower attainment and overall mental difficulties was largely driven by the presence of hyperactivity disorder which remained highly significant after accounting for other explanatory factors. The relationship between hyperactivity disorder and lower attainment is has been documented elsewhere (35). Our data support the on-going development early interventions targeted towards hyperactivity disorders (36) focussing on meeting the specific needs of children and young people to enable them to reach their academic potential. Importantly, these interventions are and ought to continue to be school-based as it offers a suitable medium for universal support and equal access to provision to nearly all young people. (37)

While males and females with overall mental difficulties were equally likely to not achieve the GCSE benchmark, this relationship was only significant for males after controlling for explanatory factors. This is concurrent with previous work on the same sample assessing educational attainment at older ages, (38) which demonstrated that females at age 18 exhibited a weaker relationship between mental difficulties and attainment than males. However, in contrast to our findings at ages 11 to 14 years, females at age 18 were significantly more likely to experience poor mental health than males - females being more likely to be conscientious high achievers was suggested as a possible explanation. Although the reason for this difference needs further investigation, these findings confirm important age and sex differences which ought to be accounted for when devising interventions aimed at promoting adolescent mental health.

It is noteworthy that although family socioeconomic circumstances are well-established predictors of later performance at school (39) the association with mental health difficulties was robust to adjustment. Although the association between poorer mental health and lower attainment operated regardless of socioeconomic background, interventions to improve mental health delivered via universal and inclusive mainstream or alternative education-based settings are likely to disproportionately impact those from disadvantaged backgrounds as they are more likely to experience mental health difficulties. Based on findings presented here, improving mental health could possibly increase average attainment levels within this group to a greater extent than within the majority population who are not disadvantaged. The potential effect at a population level would be to reduce the average difference in attainment

For peer review only - http://bmjopen.bmj.com/site/about/guidelines.xhtml

between socioeconomic groups, and narrow educational and consequent social inequalities.

Overall, these data are of interest to a range for stakeholders as they offer a contemporary and contextually rich data useful for wider policymaking and practice. Furthermore, showing the strong association between social factors with attainment and mental health makes the fully adjusted independent link between mental health and attainment all the more striking highlighting that they are both important predictors of attainment.

Limitations

Consent to data linkage and successful linkage between the UKHLS and the NPD was predicted by ethnicity, household structure and social class. The inclusion of these variables in the imputation and the final models may mitigate against some of these selection effects, the lack of an analytic weight and the ethical limitation of being unable to impute missing data for sensitive information which has been actively protected by the respondent means that data may not be representative; prevalence estimates should be interpreted cautiously and may not be generalisable to the English population. This does not, however, diminish confidence in the associations identified by the prospective approach taken. Although the collection of mental difficulties data from young people is preferable than from their parents, this information was self-reported rather than a clinical diagnosis. Other measures of wellbeing and mental health ought to be considered in future analysis as associations with different constructs may differ from those presented here. Cut points for the SDQ are contested with researchers in different contexts opting for different thresholds. The SDQ developer adds the caveats to a recently devised set of cut-points that these systems “only provide a rough-and-ready way of screening for disorders”. (40) Lastly, mediation analysis has not been conducted in this study though predictors of attainment such as happiness with school may be candidate variables. Caution should be applied to interpreting these candidate mediators as current estimates of the effect of mental difficulties on attainment may be considered overadjusted.

Contributions

NS and LM designed the analysis which was carried out by LM and MA under guidance from MS and NS. NS drafted the manuscript. AH and SS contributed to the study design and drafting of the manuscript. We thank the peer-reviewers for the constructive comments on this manuscript.

Declarations of interest

None

Funding. The project was funded by the Economic and Social Research Council Secondary Data Analysis Initiative (ES/R005400/1 to NS).

For peer review only - http://bmjopen.bmj.com/site/about/guidelines.xhtml

Competing interests: None declared.

Patient consent: Obtained.

Patient and public involvement:
It was not appropriate or possible to involve patients or the public in the design, or conduct, or reporting, or dissemination plans of our research.

Provenance and peer review: Not commissioned; externally peer reviewed.

Data Sharing Statement: Data are available in a public, open access repository. All data are hosted by the UK Data Service (UKDS):

National Pupil Database data is available under secure access licence agreement to registered and approved researchers.
10.5255/UKDA-SN-7642-2

Understanding Society Main Survey Data are available to registered users under standard terms of the UKDS End User Licence Agreement.
http://doi.org/10.5255/UKDA-SN-6614-13.

Ethics Statement: The data used are publicly available via UK Data Service repository (study numbers 6614 and 8644), and do not require ethical assessment for academic research purposes. The University of Essex Ethics Committee approved the survey data collection. No ethics approval number was produced. Ethics approval for data collection was granted by letter dated 6 July 2007 for Waves 1 and 2 and by letter dated 17 December 2010 for Waves 3 to 5

https://www.understandingsociety.ac.uk/documentation/mainstage/user-guides/main-survey-user-guide/ethics

Figure 1: Flow chart describing the breakdown of the combined Wave 1 and Wave 3 study population of the UKHLS into the analytic sample

For peer review only - http://bmjopen.bmj.com/site/about/guidelines.xhtml

1. NHS Digital. Mental Health of Children and Young People in England, 2017. [Internet]. 2018. Available from: https://digital.nhs.uk/data-and-information/publications/statistical/mental-health-of-children-and-young-people-in-england/2017/2017

2. Department of Health. Future in mind - Promoting, protecting and improving our children and young people's mental health and wellbeing [Internet]. 2015 p. 81–81. Available from: https://assets.publishing.service.gov.uk/government/uploads/system/uploads/attachment_data/file/414024/Childrens_Mental_Health.pdf

3. Chowdry H, McBride T. Disadvantage, Behaviour And Cognitive Outcomes: Longitudinal Analysis From Age 5 To 16. London; 2017.

4. Crawford C, Greaves E. Socio-economic, ethnic and gender differences in HE participation. [Internet]. Department for Business, Innovation and Skills; 2015 p. 116–116. Available from: https://assets.publishing.service.gov.uk/government/uploads/system/uploads/attachment_data/file/474273/BIS-15-85-socio-economic-ethnic-and-gender-differences.pdf

5. Lessof C, Ross A, Brind R. Multiple disadvantage and KS4 attainment: evidence from LSYPE2 [Internet]. GOV.UK. 2019 [cited 2020 Apr 7]. Available from: https://www.gov.uk/government/publications/multiple-disadvantage-and-ks4-attainment-evidence-from-lsype2

6. Schoon I. Planning for the Future: Changing Education Expectations in Three British Cohorts. Historical Social Research [Internet]. 2010;35(2). Available from: http://www.jstor.org/stable/20762452?seq=1#page_scan_tab_contents

7. Flouri E. Parental interest in children's education, children's self-esteem and locus of control, and later educational attainment: twenty-six year follow-up of the 1970 British Birth Cohort. The British journal of educational psychology. 2006;76(Pt 1):41–55.

8. Sellström E, Bremberg S. Is there a "school effect" on pupil outcomes? A review of multilevel studies. J Epidemiol Community Health. 2006 Feb;60(2):149–55.

9. Hale DR, Viner RM. How adolescent health influences education and employment: investigating longitudinal associations and mechanisms. J Epidemiol Community Health. 2018 Jun 1;72(6):465–70.

10. Dalsgaard S, McGrath J, Østergaard SD, Wray NR, Pedersen CB, Mortensen PB, et al. Association of Mental Disorder in Childhood and Adolescence With Subsequent Educational Achievement. JAMA Psychiatry. 2020 Aug 1;77(8):797–805.

11. Kessler C, Saunders B, Ph D, Foster L, Stang PE, Al E. Social Consequences of Psychiatric Disorders, I: Educational Attainment. American Journal of Psychiatry. 1995;15(July):1026–32.

For peer review only - http://bmjopen.bmj.com/site/about/guidelines.xhtml

12. Veldman K, Bültmann U, Stewart RE, Ormel J, Verhulst FC, Reijneveld SA. Mental Health Problems and Educational Attainment in Adolescence: 9-Year Follow-Up of the TRAILS Study. PLOS ONE. 2014 Jul 21;9(7):e101751.

13. L Marshall RW A Dunatchik, NR Smith. Supporting Mental Health in Schools and Colleges. https://www.gov.uk/government/publications/supporting-mental-health-in-schools-and-colleges. 2017;(ISBN 978-1-78105-757-5, DFE-RR697b).

14. Rothon C, Head J, Clark C, Klineberg E, Cattell V, Stansfeld S. The impact of psychological distress on the educational achievement of adolescents at the end of compulsory education. Social Psychiatry and Psychiatric Epidemiology. 2009 May 1;44(5):421–7.

15. Deighton J, Humphrey N, Belsky J, Boehnke J, Vostanis P, Patalay P. Longitudinal pathways between mental health difficulties and academic performance during middle childhood and early adolescence. The British journal of developmental psychology. 2018 Mar 1;36(1):110–26.

16. Clayborne ZM, Varin M, Colman I. Systematic Review and Meta-Analysis: Adolescent Depression and Long-Term Psychosocial Outcomes. J Am Acad Child Adolesc Psychiatry. 2019 Jan;58(1):72–9.

17. Sellers R, Warne N, Pickles A, Maughan B, Thapar A, Collishaw S. Cross-cohort change in adolescent outcomes for children with mental health problems. Journal of Child Psychology and Psychiatry. 2019 Jul 31;60(7):813–21.

18. Marshall L, Wishart R, Dunatchik A, Smith N. Supporting Mental Health in Schools and Colleges [Internet]. 2017 [cited 2020 Jul 4]. Available from: https://assets.publishing.service.gov.uk/government/uploads/system/uploads/attachment_data/file/634726/Supporting_Mental-Health_survey_report.pdf

19. University of Essex, Institute for Social and Economic Research, NatCen Social Research, Kantar Public. Understanding Society: Waves 1-9, 2009-2018 and Harmonised BHPS: Waves 1-18, 1991-2009. [data collection]. 12th Edition. UK Data Service. 2019. 2019.

20. Institute for Social and Economic Research. Understanding Society: The UK Household Longitudinal Study, Waves 1-9 - User Guide [Internet]. University of Essex; 2019 [cited 2020 Sep 4]. Available from: https://www.understandingsociety.ac.uk/sites/default/files/downloads/documentation/mainstage/user-guides/mainstage-user-guide.pdf

21. Jay MA, Grath-Lone LM, Gilbert R. Data Resource: the National Pupil Database (NPD). International Journal of Population Data Science [Internet]. 2019 Mar 20 [cited 2021 Jun 7];4(1). Available from: https://ijpds.org/article/view/1101

22. Knies G, Burton J. Analysis of four studies in a comparative framework reveals: health linkage consent rates on British cohort studies higher than

For peer review only

For peer review only - http://bmjopen.bmj.com/site/about/guidelines.xhtml

on UK household panel surveys. BMC medical research methodology. 2014 Nov;14:125–125.

23. Department for Education. GCSE and equivalent results: 2011 to 2012 (revised) [Internet]. 2013 Jan [cited 2021 Jun 7]. Available from: https://www.gov.uk/government/statistics/revised-gcse-and-equivalent-results-in-england-academic-year-2011-to-2012

24. Goodman R. The Strengths and Difficulties Questionnaire: a research note. Journal of Child Psychology and Psychiatry. 1997;38(5):581–6.

25. Biederman J, Monuteaux MC, Doyle AE, Seidman LJ, Wilens TE, Ferrero F, et al. Impact of Executive Function Deficits and Attention-Deficit/Hyperactivity Disorder (ADHD) on Academic Outcomes in Children. Journal of Consulting and Clinical Psychology. 2004 Oct;72(5):757–66.

26. Woodward LJ, Fergusson DM. Childhood peer relationship problems and later risks of educational under-achievement and unemployment. Journal of child psychology and psychiatry, and allied disciplines. 2000 Feb;41(2):191–201.

27. Deighton J, Lereya ST, Casey P, Patalay P, Humphrey N, Wolpert M. Prevalence of mental health problems in schools: poverty and other risk factors among 28 000 adolescents in England. British Journal of Psychiatry. 2019;215(3):565–7.

28. Strand S. The limits of social class in explaining ethnic gaps in educational attainment. British Educational Research Journal. 2011;37(2):197–229.

29. Goodman A, Gregg P. Poorer children's educational attainment: how important are attitudes and behaviour? [Internet]. JRF. 2010 [cited 2021 Jun 7]. Available from: https://www.jrf.org.uk/report/poorer-children%E2%80%99s-educational-attainment-how-important-are-attitudes-and-behaviour

30. Carneiro P, Meghir C, Parey M. Maternal Education, Home Environments, and the Development of Children and Adolescents. Journal of the European Economic Association. 2013 Jan 1;11(suppl_1):123–60.

31. Rose D, Pevalin DJ. The National Statistics Socio-economic Classification (NS-SEC rebased on the SOC2010). Palgrave Macmillan; 2011.

32. Knies G. Income effects on children's life satisfaction: Longitudinal evidence for England [Internet]. ISER Working Paper Series; 2017 [cited 2021 Jun 7]. Report No.: 2017–02. Available from: https://www.econstor.eu/handle/10419/163545

33. Ware Jr JE, Kosinski M, Keller SD. A 12-Item Short-Form Health Survey: construction of scales and preliminary tests of reliability and validity. Medical care. 1996;34(3):220–33.

For peer review only - http://bmjopen.bmj.com/site/about/guidelines.xhtml

34. Suldo SM, Riley KN, Shaffer EJ. Academic Correlates of Children and Adolescents' Life Satisfaction. School Psychology International. 2006 Dec 1;27(5):567–82.

35. Birchwood J, Daley D. Brief report: The impact of Attention Deficit Hyperactivity Disorder (ADHD) symptoms on academic performance in an adolescent community sample. Journal of Adolescence. 2012 Feb 1;35(1):225–31.

36. May F, Ford T, Janssens A, Newlove-Delgado T, Russell AE, Salim J, et al. Attainment, attendance, and school difficulties in UK primary schoolchildren with probable ADHD. British Journal of Educational Psychology. 2021;91(1):442–62.

37. Department for Education, Department of Health and Social Care. Transforming children and young people's mental health provision: a green paper [Internet]. GOV.UK. 2018 [cited 2021 Jun 7]. Available from: https://www.gov.uk/government/consultations/transforming-children-and-young-peoples-mental-health-provision-a-green-paper

38. Tregaskis O, Nandi A, Watson D. An examination of the relationship between adolescent mental health and educational outcomes in early adulthood: Secondary analysis of Understanding Society data wave 1 to wave 8. 2020.

39. Benton T, Such, T. Analysis of use of Key Stage 2 data in GCSE predictions [Internet]. OFQUAL; 2014 [cited 2021 Jun 7]. Available from: https://assets.publishing.service.gov.uk/government/uploads/system/uploads/attachment_data/file/429074/2014-06-16-analysis-of-use-of-key-stage-2-data-in-gcse-predictions.pdf

40. Goodman R. Scoring the Strengths & Difficulties Questionnaire for age 4-17 or 18+ [Internet]. Youth in Mind. [cited 2021 Jun 7]. Available from: https://www.sdqinfo.org/py/sdqinfo/b3.py?language=Englishqz(UK)

For peer review only

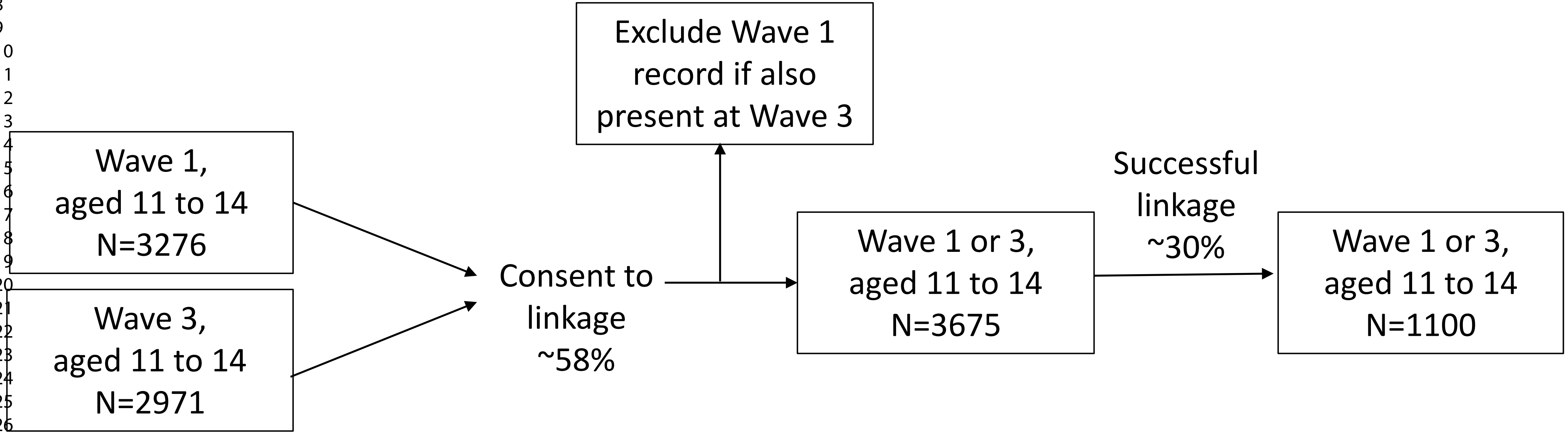
Exclude Wave 1
record if also
present at Wave 3
Wave 1,
aged 11 to 14
N=3276
Wave 3,
aged 11 to 14
N=2971
Consent to
linkage
~58%
Wave 1 or 3,
aged 11 to 14
N=3675
Successful
linkage
~30%
Wave 1 or 3,
aged 11 to 14
N=1100

Supplementary table A: Odds ratios for low attainment at Key Stage 4 by total mental health difficulties and domain scores, adjusted stepwise for explanatory factors, by sex.

	Emotional		Peer		Conduct		Hyperactivity		Total score	
	Males	Females	Males	Females	Males	Females	Males	Females	Males	Females
Model 1	**3.07** [1.48,6.38]**	1.49 [0.91,2.43]	**2.36** [1.39,4.02]**	**2.55** [1.45,4.48]**	**1.65* [1.03,2.66]**	**2.17** [1.22,3.86]**	**2.35*** [1.49,3.71]**	**2.63*** [1.59,4.35]**	**3.16*** [1.79,5.60]**	**3.36*** [1.97,5.71]**
Model 2	**3.22** [1.55,6.71]**	1.54 [0.95,2.50]	**2.43** [1.43,4.14]**	**2.78*** [1.59,4.86]**	**1.71* [1.06,2.76]**	**2.15* [1.18,3.89]**	**2.40*** [1.51,3.79]**	**2.61*** [1.56,4.37]**	**3.30*** [1.85,5.87]**	**3.47*** [2.03,5.92]**
Model 3	**2.91* [1.17,7.22]**	1.41 [0.77,2.57]	**2.34* [1.20,4.56]**	1.31 [0.61,2.79]	1.56 [0.86,2.85]	**2.67** [1.35,5.26]**	**2.40** [1.36,4.24]**	**3.45*** [1.90,6.27]**	**3.39*** [1.74,6.62]**	**3.91*** [2.04,7.51]**
Model 4	**2.89* [1.05,7.92]**	1.23 [0.63,2.42]	**2.23* [1.07,4.63]**	1.26 [0.55,2.90]	1.44 [0.76,2.72]	**2.42* [1.16,5.05]**	**2.68** [1.43,5.04]**	**3.43*** [1.75,6.73]**	**3.38** [1.64,6.98]**	**3.52*** [1.69,7.32]**
Model 5	2.37 [0.85,6.59]	0.98 [0.49,1.97]	1.90 [0.93,3.87]	1.12 [0.48,2.60]	1.03 [0.50,2.12]	1.82 [0.85,3.93]	**2.23* [1.15,4.31]**	**2.64** [1.35,5.18]**	**2.66* [1.25,5.70]**	**2.61* [1.22,5.57]**
Model 6	2.51 [0.87,7.28]	0.76 [0.35,1.65]	1.85 [0.88,3.90]	1.09 [0.45,2.64]	0.98 [0.45,2.14]	1.35 [0.54,3.32]	**2.17* [1.13,4.19]**	**2.73* [1.24,6.03]**	**2.86** [1.30,6.29]**	1.79 [0.76,4.25]
Model 7	2.36 [0.83,6.64]	0.73 [0.34,1.57]	1.79 [0.83,3.84]	0.99 [0.41,2.40]	0.93 [0.42,2.05]	1.29 [0.52,3.18]	**2.17* [1.11,4.23]**	**2.85** [1.30,6.23]**	**2.77* [1.24,6.16]**	1.69 [0.72,3.95]

Note: Imputed model, Males N=550; Females N=560

Model 1: unadjusted odds of socioemotional difficulties; Model 2: adjusts for Model 1 + age, ethnicity; Model 3: adjusts for Model 2 + prior attainment at KS2; Model 4: adjusts for Model 3 + household social class, maternal education, household poverty, family composition Model 5: adjusts for Model 4 + happy with school work, happy with school; Model 6: adjusts Model 5 + parental interest in school, parents attend parent evening, family support, quarrels with parents; Model 7: adjusts for Model 6 +parental mental and physical health.

Significant odds ratios (95% confidence interval) shown in bold text.

For peer review only

STROBE 2007 (v4) Statement—Checklist of items that should be included in reports of *cohort studies*

Section/Topic	Item #	Recommendation	Reported on page #
**Title and abstract**	1	(*a*) Indicate the study's design with a commonly used term in the title or the abstract	0
		(*b*) Provide in the abstract an informative and balanced summary of what was done and what was found	1
**Introduction**			
Background/rationale	2	Explain the scientific background and rationale for the investigation being reported	3
Objectives	3	State specific objectives, including any prespecified hypotheses	4
**Methods**			
Study design	4	Present key elements of study design early in the paper	4, 5
Setting	5	Describe the setting, locations, and relevant dates, including periods of recruitment, exposure, follow-up, and data collection	5
Participants	6	(*a*) Give the eligibility criteria, and the sources and methods of selection of participants. Describe methods of follow-up	5
		(*b*) For matched studies, give matching criteria and number of exposed and unexposed	n/a
Variables	7	Clearly define all outcomes, exposures, predictors, potential confounders, and effect modifiers. Give diagnostic criteria, if applicable	5, 6
Data sources/ measurement	8*	For each variable of interest, give sources of data and details of methods of assessment (measurement). Describe comparability of assessment methods if there is more than one group	5, 6
Bias	9	Describe any efforts to address potential sources of bias	6, 15
Study size	10	Explain how the study size was arrived at	6
Quantitative variables	11	Explain how quantitative variables were handled in the analyses. If applicable, describe which groupings were chosen and why	5, 6
Statistical methods	12	(*a*) Describe all statistical methods, including those used to control for confounding	6, 7
		(*b*) Describe any methods used to examine subgroups and interactions	7
		(*c*) Explain how missing data were addressed	7
		(*d*) If applicable, explain how loss to follow-up was addressed	5, 7
		(*e*) Describe any sensitivity analyses	n/a
**Results**			

For peer review only

Participants	13*	(a) Report numbers of individuals at each stage of study—eg numbers potentially eligible, examined for eligibility, confirmed eligible, included in the study, completing follow-up, and analysed	5, 8, 9. 10, 11
		(b) Give reasons for non-participation at each stage	5
		(c) Consider use of a flow diagram	5
Descriptive data	14*	(a) Give characteristics of study participants (eg demographic, clinical, social) and information on exposures and potential confounders	8-11
		(b) Indicate number of participants with missing data for each variable of interest	8-11
		(c) Summarise follow-up time (eg, average and total amount)	n/a
Outcome data	15*	Report numbers of outcome events or summary measures over time	8-11
Main results	16	(*a*) Give unadjusted estimates and, if applicable, confounder-adjusted estimates and their precision (eg, 95% confidence interval). Make clear which confounders were adjusted for and why they were included	12, 13
		(*b*) Report category boundaries when continuous variables were categorized	5, 6
		(*c*) If relevant, consider translating estimates of relative risk into absolute risk for a meaningful time period	n/a
Other analyses	17	Report other analyses done—eg analyses of subgroups and interactions, and sensitivity analyses	13
**Discussion**			
Key results	18	Summarise key results with reference to study objectives	14
**Limitations**			
Interpretation	20	Give a cautious overall interpretation of results considering objectives, limitations, multiplicity of analyses, results from similar studies, and other relevant evidence	14,15
Generalisability	21	Discuss the generalisability (external validity) of the study results	15
**Other information**			
Funding	22	Give the source of funding and the role of the funders for the present study and, if applicable, for the original study on which the present article is based	15,16

*Give information separately for cases and controls in case-control studies and, if applicable, for exposed and unexposed groups in cohort and cross-sectional studies.

Note: An Explanation and Elaboration article discusses each checklist item and gives methodological background and published examples of transparent reporting. The STROBE checklist is best used in conjunction with this article (freely available on the Web sites of PLoS Medicine at http://www.plosmedicine.org/, Annals of Internal Medicine at http://www.annals.org/, and Epidemiology at http://www.epidem.com/). Information on the STROBE Initiative is available at www.strobe-statement.org.

For peer review only

BMJ Open

Adolescent mental health difficulties and educational attainment: findings from the UK Household Longitudinal Study

Journal:	*BMJ Open*
Manuscript ID	bmjopen-2020-046792.R3
Article Type:	Original research
Date Submitted by the Author:	21-Jun-2021
Complete List of Authors:	SMITH, NEIL; NatCen Social Research, Policy Research Centre<br>Marshall, Lydia; NatCen Social Research, Policy Research Centre<br>Albakri, Muslihah; NatCen Social Research, Policy Research Centre<br>Smuk, Melanie; London School of Hygiene & Tropical Medicine<br>Hagell, Ann; Association for Young People's Health<br>Stansfeld, Stephen; Barts and the London, Queen Marys School of Medicine and Dentistry, Psychiatry
**Primary Subject Heading**:	Epidemiology
Secondary Subject Heading:	Mental health
Keywords:	MENTAL HEALTH, PUBLIC HEALTH, Child & adolescent psychiatry < PSYCHIATRY, EPIDEMIOLOGY

SCHOLARONE™
Manuscripts

I, the Submitting Author has the right to grant and does grant on behalf of all authors of the Work (as defined in the below author licence), an exclusive licence and/or a non-exclusive licence for contributions from authors who are: i) UK Crown employees; ii) where BMJ has agreed a CC-BY licence shall apply, and/or iii) in accordance with the terms applicable for US Federal Government officers or employees acting as part of their official duties; on a worldwide, perpetual, irrevocable, royalty-free basis to BMJ Publishing Group Ltd ("BMJ") its licensees and where the relevant Journal is co-owned by BMJ to the co-owners of the Journal, to publish the Work in this journal and any other BMJ products and to exploit all rights, as set out in our licence.

The Submitting Author accepts and understands that any supply made under these terms is made by BMJ to the Submitting Author unless you are acting as an employee on behalf of your employer or a postgraduate student of an affiliated institution which is paying any applicable article publishing charge ("APC") for Open Access articles. Where the Submitting Author wishes to make the Work available on an Open Access basis (and intends to pay the relevant APC), the terms of reuse of such Open Access shall be governed by a Creative Commons licence – details of these licences and which Creative Commons licence will apply to this Work are set out in our licence referred to above.

Other than as permitted in any relevant BMJ Author's Self Archiving Policies, I confirm this Work has not been accepted for publication elsewhere, is not being considered for publication elsewhere and does not duplicate material already published. I confirm all authors consent to publication of this Work and authorise the granting of this licence.

For peer review only

Adolescent mental health difficulties and educational attainment: findings from the UK Household Longitudinal Study

Corresponding author:
Neil R Smith, PhD
NatCen Social Research
Policy Research Centre
35 Northampton Square
London
EC1V 0AX, UK
Neil.Smith@natcen.ac.uk

Lydia Marshall, PhD
NatCen Social Research
Policy Research Centre
35 Northampton Square
London
EC1V 0AX, UK
Lydia.Marshall@natcen.ac.uk

Muslihah Albakri
NatCen Social Research
Policy Research Centre
35 Northampton Square
London
EC1V 0AX, UK
Muslihah.Albakri@natcen.ac.uk

Melanie Smuk, PhD
London School of Hygiene & Tropical Medicine
Keppel Street
London
WC1E 7HT, UK
Melanie.Smuk@lshtm.ac.uk

Ann Hagell, PhD
Association for Young People's Health
7-14 Great Dover Street
London
SE1 4YR, UK
Ann@youngpeopleshealth.org.uk

Stephen Stansfeld, PhD
Centre for Psychiatry
Bart's and the London School of Medicine and Dentistry
London
EC1M 6BQ, UK
S.A.Stansfeld@qmul.ac.uk

3076 words

Keywords: mental health, adolescence, education, social epidemiology, life course

For peer review only - http://bmjopen.bmj.com/site/about/guidelines.xhtml

ABSTRACT

Objective: This study examines whether there is an independent association between mental difficulties in adolescence and educational attainment at age 16.

Design: Longitudinal study.

Setting: Nationally representative data from the UK Household Longitudinal Study (UKHLS) were linked to the National Pupil Database for England.

Participants: Respondents (N=1,100) to the UKHLS between 2009-2012 were linked to the National Pupil Database to investigate longitudinal associations between mental difficulties at ages 11-14 and educational attainment at age 16 (GCSE).

Primary outcome measure: Not gaining five or more GCSE qualifications at age 16, including English and maths at grade A*-C.

Results: An atypical total mental health difficulties score measured using the Strengths and Difficulties Questionnaire at ages 11-14 predicted low levels of educational attainment at age 16 (OR: 3.11 (95% CI: [2.11, 4.57]). Controlling for prior attainment and family sociodemographic factors, happiness with school(/work) and parental health, school engagement and relationship with the child partially attenuated the association which was significant in the fully adjusted model (2.05, 95% CI: [1.15,3.68]). The association was maintained in the fully adjusted model for males only (OR: 2.77 (95% CI: [1.24, 6.16]) but not for females. Hyperactivity disorder strongly predicted lower attainment for males (OR: 2.17 (95% CI: [1.11, 4.23]) and females (OR: 2.85 (95% CI: [1.30, 6.23]).

Conclusion: Mental difficulties at ages 11-14 were independently linked to educational success at age 16, highlighting an important pathway through which health in adolescence can determine young people's life chances.

For peer review only

STRENGTHS AND LIMITATIONS OF THIS STUDY.

- This is a large, nationally representative longitudinal cohort study containing self-assessed measures of mental health among young people linked to a National Pupil Database of educational records.
- The study captures a diverse range of social, demographic, economic and behavioural factors affecting young people in their home and school environment, permitting statistical adjustment for multiple confounding relationships which might explain the association between mental health and educational attainment.
- Consent to data linkage between the longitudinal study and the National Pupil Database was incomplete, though factors which predicted patterns of non-consent were controlled for within our models.
- Missing data was accounted for using multiple imputation methods which exploited the wide range of associations within the observed data to minimise errors within estimates of effect.

For peer review only

INTRODUCTION

Growing evidence of the prevalence of poor child and adolescent mental health has led to this issue becoming a key policy priority in the UK. Mental ill-health in children and young people in England increases age with around 14.4% of 11-16 years experiencing a mental disorder compared to 5.5% in their pre-school counterparts aged 2-4 years.(1) With 75% of adult mental health problems (excluding dementia) starting by the age of 18,(2) adolescence is a key period in the development of long-lasting mental health difficulties. The UK government's *Future in Mind* report (2) presented an important economic case for investment in early prevention of mental ill-health to mitigate against the costs of longer-term support for health needs. However, this argument neglects the impact that early life mental health potentially has on other early life outcomes fundamental in determining life chances, such as educational attainment.(3) Educational outcomes are closely associated with later-life chances with well-established links to employment, income, housing and offending as well as physical health and on-going mental health disorders. If poor mental health diminishes the capacity for individuals to fulfil their academic potential, mental health itself is likely to be a driver of educational inequality and consequent on-going social inequality.

On the other hand, the association between mental health and educational outcomes might not be direct, but rather incorporate the influence of confounding factors. A range of demographic and socioeconomic factors, such as gender, ethnicity, socioeconomic disadvantage and maternal education and parental health(4–6) have known relationships with educational attainment and must be accounted for when assessing the impact of poor mental health. Similarly, the home environment and specifically parental interest in schooling has been associated with higher attainment,(7) as have positive environmental "school effects", (8) whereas lower attainment has been associated with absence from school (1) or poor classroom behaviours. (9) What is less clear is the extent to which differential exposure to these factors also underpin disparities in mental health, and whether resulting differences in mental health might influence differences in attainment.

International research has demonstrated numerous associations between mental health and educational attainment.(10–12). The evidence base for England is less well-established which is of particular relevance during a time of policy interest in boosting mental health provision in schools.(13) There is some evidence of longitudinal associations between psychological distress in early adolescence and achievement at GCSE in England.(14,15) Similarly, poor mental health between ages 13 and 15 has been shown to be associated with low GCSE attainment and later unemployment, (9) demonstrating how the effects of poor early life mental health can extend into adulthood (16). Though many of these findings support the association between mental health and educational outcomes, they are often of low generalisability being based on

For peer review only - http://bmjopen.bmj.com/site/about/guidelines.xhtml

regional data or non-probability samples (14) or unable to account for a range of potentially explanatory factors. (15) There appears to be a strengthening of the relationship between adolescent mental health and educational outcomes in recent generations (17) so there is a pressing need for an up-to-date examination of nationally representative data for England.

Therefore, this study uses a novel and contemporary data linkage between the nationally representative UK Household Longitudinal Study linked to objectively measured official education records, to test associations between poor mental health and poor educational attainment. The study is significant in estimating the extent to which mental health in early adolescence has an independent association with attainment at age 16 in England in males and females. Robust evidence of a causal relationship between poor mental health and lower academic attainment could be crucial in inspiring investment in researching “what works” in supporting children and adolescents’ mental health. Although schools already appreciate the importance of supporting pupils’ health and wellbeing, (18) a proven link to academic outcomes could also encourage education and public health policymakers to invest more in mental health.

For peer review only

For peer review only - http://bmjopen.bmj.com/site/about/guidelines.xhtml

METHODS

UK Household Longitudinal Study (UKHLS)

The UKHLS is a nationally representative household panel survey (19) which began in 2009, aiming to understand social and economic change in Britain at the household and individual levels. Each wave of the survey collects information on approximately 100,000 individuals from 40,000 households, with adult household residents (aged 16 and over) responding using computer-assisted interview and self-completion questionnaire. Young people aged between 10-15 were offered a self-completion questionnaire. Further detail on the sampling design and data collection is available. (20) National educational records from the from the National Pupil Database (NPD) (21) for school-age children between ages 3 and 18 were linked to the UKHLS if parents and their children were living in England and consented to linkage at wave 1. Linkage consent rates did not differ systematically by parental class, or parental education though they were lower within ethnic minority groups which is consistent with other cohort studies. (22)

This analysis used a nationally representative sample of 11 to 14 year olds present at wave 1 (2009-2011) and wave 3 (2011-2013) linked to the NPD. Wave 2 (2010-2012) was excluded as it did not ask for information about mental health. Where respondents were present at both waves, data from wave 3 was selected as the respondent was further into adolescence. Figure 1 tracks the study population down to the final analytic sample.

The final sample consisted of all consenting youth panel respondents aged 11 to 14 years with data on mental health in wave 1 or wave 3 of UKHLS as well as NPD data on GCSE scores at ages 15 or 16 years (N=1110). The analytic sample covers England only due to the limited geographical coverage of the NPD.

Educational attainment

The primary outcome was a binary variable indicating low educational attainment, defined as whether the young person did not achieve 5 or more grades A*-C for the General Certificate of Secondary Education (GCSE), including English and maths. This was the benchmark measure of educational attainment at Key Stage 4 (KS4) at secondary schools in England during the study period. (23)

Mental difficulties

Young people completed the Strengths and Difficulties questionnaire (SDQ) validated for ages 4-15 years. (24) The SDQ asks questions about four domains of negative behaviours which have varying strengths of association with educational attainment, namely: conduct problems (11); hyperactivity (25); emotional symptoms (14); peer problems. (26) Scores from the four subscales were summed to construct a total difficulties score, where a higher score refers to a greater level of mental difficulties. Binary measures of mental difficulties were

For peer review only - http://bmjopen.bmj.com/site/about/guidelines.xhtml

derived based on developer guidance. (24) An "atypical" level of total difficulties was derived from the top 10% of the population scores (>=18 out of 40) and individual SDQ domains used validated "atypical" cut points which have also been used in a recent prevalence survey in England. (27)

Explanatory variables

We focussed on risk factors where the literature has established potentially causal associations with educational attainment and mental health respectively. All analyses were controlled for gender, age, ethnic group as well as the three tiered classification household's highest parental occupational class, household deprivation and mother's highest educational qualifications. (28–30) Parents' highest current or previous occupational class was based on the National Statistics Socioeconomic Classification (NS-SEC) which was collapsed into a three-tier hierarchical scale (professional/managerial; intermediate; manual/routine) (31) with an additional category for overseas or no qualifications. The mother's highest qualification was summarised on a three-tier hierarchical scale (degree or higher; A-level or equivalent; GCSE or equivalent with a separate category for none or other. Household poverty was derived based on income poverty, material poverty, subjective poverty and the receipt of benefits and was categorised into 'not at all deprived', 'somewhat deprived' or 'highly deprived'.(32) Additionally, family type was grouped into two parent households, lone parent household or other family types.(28)

Parental relationships were assessed using a binary measures of young people's self-reports on how interested their parent(s) were at how they did at school, attendance at parents' evenings, frequency of quarrelling with either parent(s) and how often they feel supported by their family. (7) Parental physical and mental health was assessed using the SF-12 Physical and Mental Component Summary respectively, (33) with a score from either parent in the lowest quintile representing poor physical health and a mental health score of >=45.6 representing poor mental health.

Young people reported levels of happiness specifically with school-work as well as with school generally on a 7-point scale with a score of 5 or greater indicating happiness. (34) Prior attainment was measured based on whether young people achieved the expected level 4 reading, writing and mathematics at Key Stage 2 (KS2) (ages 10-11 years).

All non-educational attainment measures were taken at the time adolescent mental health was assessed.

Statistical analysis

Complete data was available was available for age, sex, ethnicity and family composition. Missing data was most common for household poverty (13%) so data was imputed under the missing at random assumption as poverty was associated with poorer explanatory outcomes, specifically lower level of

For peer review only - http://bmjopen.bmj.com/site/about/guidelines.xhtml

occupational class, maternal education, family composition and prior attainment. Given the overall low level of missingness, twenty imputed datasets were created. All explanatory variables and measures of mental difficulties shown in Table 1 were used in the imputation and missing data for explanatory variables (ranging between 1% and 13%) and mental difficulties (0.2%) was imputed. Data on GCSE grades were not imputed due to a high proportion of missing data (70%) due to a lack of linkage consent, and for ethical reasons given these individuals had not consented to their data being used for research.

The prevalence of low attainment and mental difficulties are described separately according to a range of selected socioeconomic, demographic and parent-related factors. Data was weighted using the cross-sectional self-completion weights in the UKHLS youth panel in wave 1 and wave 3.

Logistic regression was used to estimate separately the odds ratio of not achieving 5 A*-C GCSE grades including English and mathematics and of being classed as having mental difficulties. Stepwise regression models adjusted the odds ratios of having total mental difficulties and difficulties within each domain to examine the relative impact of prior attainment, sociodemographic factors, parent-child relationships, young person's happiness with school and parental health on educational attainment. Models were stratified to explore gender differences in total and domain specific mental difficulties. All analyses were performed in Stata v16.1 (StataCorp, College Station, TX, USA).

For peer review only

RESULTS

The analytic sample was evenly split by gender and the overwhelming majority were aged 13 or 14 years old. Respondents tended to be from relatively socioeconomically advantaged backgrounds. A third of mothers were degree educated and 41.8% of households belonged to the highest social class. Over three-quarters of the sample reported high parental engagement with school and happiness with school-work. Prior attainment levels were positive for reading (93.3%), writing (82.6%) and maths (71.5%).

The proportion of young people not achieving the Key Stage 4 (KS4) benchmark of 5 GCSEs A*-C including English and maths varied by selected characteristics (Table 2). Low prior attainment at Key Stage 2 (KS2) was most strongly associated with not reaching the educational benchmark at KS4. Low attainment at KS4 was also associated with lower social class, lower maternal education, higher household poverty scores and poorer parent-child relationships as well as poor parental mental and physical health. Reported unhappiness with school and school-work, and lower parental involvement in schooling was also significantly associated with low attainment.

Table 1: Prevalence % of low educational attainment at Key Stage 4 by sociodemographic and parental characteristics.

	% (N)	Low attainment %	Odds ratio	95% CI
Sex				
Male	51.6 (550)	42.0	1	Ref
Female	48.4 (560)	31.5***	0.64***	[0.49,0.83]
Age (years)				
11	1.1 (14)	65.5*	3.42*	[1.05,11.15]
12	9.7 (111)	38.4	1.12	[0.72,1.76]
13	38.9 (432)	37.3	1.07	[0.81,1.42]
14	50.4 (553)	35.7	1.00	Ref
Ethnic group				
White British	86.1 (839)	36.9	1	Ref
Other ethnic group	13.9 (271)	37.0	1.00	[0.72,1.40]
Parental highest social class (NS-SEC)				
Management & professional	41.8 (439)	23.4	1.00	Ref
Intermediate	22.7 (253)	34.2**	1.70**	[1.19,2.44]
Routine & manual	31.0 (345)	53.6**	3.79***	[2.74,5.25]
Unemployed	4.4 (53)	61.3**	5.18***	[2.60,10.35]
Mother's highest qualification				
Degree or higher	33.2 (351)	24.0	1	Ref
A-level or equivalent	17.5 (185)	21.8	0.88	[0.57,1.38]

For peer review only

GCSE or equivalent	29.5 (309)	41.3***	2.23***	[1.57,3.19]
None/other	19.8 (239)	65.4***	6.00***	[4.06,8.86]
Household poverty score				
Not at all deprived	20.9 (179)	16.2	1	Ref
Somewhat deprived	54.0 (493)	35.8***	2.89***	[1.84,4.56]
Highly deprived	25.1 (266)	56.5***	6.74***	[4.08,11.13]
Family composition				
Two-parent	69.7 (759)	32.9	1	Ref
Single parent	27.8 (321)	47.6***	1.86***	[1.39,2.47]
Other	2.5 (30)	suppressed	-	-
Happy with school-work				
Happy	74.7 (840)	29.6	1	Ref
Not happy	25.3 (263)	58.6***	3.38***	[2.49,4.57]
Happy with school				
Happy	78.6 (876)	32.0	1	Ref
Not happy	21.4 (220)	54.7***	2.57***	[1.86,3.53]
Parental interest in school				
Always or nearly always	79.0 (871)	34.4	1	Ref
Sometimes or rarely	21.0 (220)	46.4**	1.66**	[1.20,2.28]
Regularly attends parents' evenings				
Always or nearly always	81.1 (896)	29.6	1	Ref
Sometimes or rarely	18.9 (199)	68.0***	5.05***	[3.56,7.16]
Feels supported by family				
Always or mostly	76.3 (837)	34.7	1	Ref
Not supported	23.7 (269)	44.1*	1.49*	[1.10,2.02]
Regularly quarrels with either parent				
Less than once a week	60.0 (662)	33.1	1	Ref
More than once a week	40.0 (423)	42.6**	1.50**	[1.14,1.97]
Parental mental health				
Not poor	56.8 (539)	30.0	1	Ref
Poor	43.2 (423)	46.0***	1.98***	[1.50,2.62]
Parental physical health				
Not poor	58.6 (564)	32.9	1	Ref
Poor	41.4 (402)	42.6**	1.52**	[1.15,2.00]
Attainment at Key Stage 2 Maths				
Achieved level 4	71.5 (860)	26.6	1	Ref
Did not achieve level 4	17.4 (169)	85.9***	16.92***	[10.65,26.87]
Attainment at Key Stage 2 Writing				
Achieved level 4	82.6 (270)	22.2	1	Ref
Did not achieve level 4	28.4 (759)	73.9***	9.96***	[7.14,13.90]
Attainment at Key Stage 2 Reading				
Achieved level 4	92.3 (947)	32.4	1	Ref
Did not achieve level 4	7.7 (74)	91.5***	22.65***	[9.85,52.09]

For peer review only

For peer review only - http://bmjopen.bmj.com/site/about/guidelines.xhtml

Notes: Ref=Reference group; Unweighted N; Imputed and weighted percentages shown; low educational attainment defined as < 5 GCSEs at A*-C including English and maths; some values are suppressed due to small base sizes and risk of disclosure; *** p<0.001, **p<0.01, *p<0.05

There was a similar patterning to the prevalence of mental difficulties (Table 2). Poorer household socioeconomic circumstances, parental engagement with school and health, parent-child relationships and the young person's happiness with school and school-work were all significantly associated with increased odds of being classified with mental difficulties. However, there was no significant difference in the prevalence of mental health difficulties by sex, and the association between prior attainment and current mental difficulties was relatively weak and significant only for writing at KS2.

Table 2: Prevalence % of mental difficulties by sociodemographic and parental characteristics.

	% (N)	SDQ score >=18 %	Odds ratio	95% CI
Sex				
Male	51.6 (550)	12.1	1	Ref
Female	48.4 (560)	15.0	1.28	[0.88,1.86]
Age (years)				
11	1.1 (14)	35.3*	3.79*	[1.11,12.93]
12	9.7 (111)	18.4	1.57	[0.86,2.86]
13	38.9 (432)	12.9	1.03	[0.68,1.55
14	50.4 (553)	12.6	1	Ref
Ethnic group				
White British	86.1 (839)	14.1	1	Ref
Other ethnic group	13.9 (271)	9.6	0.65	[0.37,1.11]
Parental highest social class (NS-SEC)				
Management & professional	41.8 (439)	9.0	1	Ref
Intermediate	22.7 (253)	14.0	1.64	[0.99,2.74]
Routine & manual	31.0 (345)	17.3**	2.11**	[1.34,3.33]
Unemployed	4.4 (53)	26.9**	3.71**	[1.56,8.84]
Mother's highest qualification				
Degree or higher	33.2 (351)	11.1	1	Ref
A-level or equivalent	17.5 (185)	11.1	1.00	[0.55,1.84]
GCSE or equivalent	29.5 (309)	13.3	1.23	[0.75,2.01]
None/other	19.8 (239)	20.0**	2.00**	[1.20,3.33]
Household poverty score				
Not at all deprived	20.9 (179)	8.0	1	Ref
Somewhat deprived	54.0 (493)	11.6	1.50	[0.78,2.88]
Highly deprived	25.1 (266)	22.1***	3.26***	[1.67,6.36]
Family composition				
Two-parent	69.7 (759)	12.0	1	Ref

For peer review only - http://bmjopen.bmj.com/site/about/guidelines.xhtml

Single parent	27.8 (321)	18.5*	1.66*	[1.12,2.47]
Other	2.5 (30)	suppressed	-	-
Happy with school-work				
Happy	74.7 (840)	9.0	1	Ref
Not happy	25.3 (263)	26.8***	3.71***	[2.52,5.47]
Happy with school				
Happy	78.6 (876)	9.3	1	Ref
Not happy	21.4 (220)	28.9***	3.96***	[2.66,5.90]
Parental interest in school				
Always or nearly always	79.0 (871)	10.6	1	Ref
Sometimes or rarely	21.0 (220)	24.4***	2.73***	[1.81,4.10]
Regularly attends parents' evenings				
Always or nearly always	81.1 (896)	10.8	1	Ref
Sometimes or rarely	18.9 (199)	24.9***	2.73***	[1.79,4.16]
Feels supported by family				
Always or mostly	76.3 (837)	9.0	1	Ref
Not supported	23.7 (269)	27.8***	3.87***	[2.62,5.71]
Regularly quarrels with either parent				
Less than once a week	60.0 (662)	7.5	1	Ref
More than once a week	40.0 (423)	22.5***	3.59***	[2.40,5.36]
Parental mental health				
Not poor	56.8 (539)	11.3	1	Ref
Poor	43.2 (423)	16.4*	1.55*	[1.02,2.36]
Parental physical health				
Not poor	58.6 (564)	11.3	1	Ref
Poor	41.4 (402)	16.6*	1.57*	[1.04,2.37]
Attainment at Key Stage 2 Maths				
Achieved level 4	71.5 (860)	12.5	1	Ref
Did not achieve level 4	17.4 (169)	18.2	1.56	[0.98,2.48]
Attainment at Key Stage 2 Writing				
Achieved level 4	82.6 (270)	11.5	1	Ref
Did not achieve level 4	28.4 (759)	18.4**	1.72**	[1.15,2.58]
Attainment at Key Stage 2 Reading				
Achieved level 4	92.3 (947)	13.4	1	Ref
Did not achieve level 4	7.7 (74)	15.1	1.15	[0.56,2.37]

Notes: Ref=Reference group; Unweighted N; Imputed and weighted percentages shown; some values are suppressed due to small base sizes and risk of disclosure; *** p<0.001, **p<0.01, *p<0.05

Young people classified with mental health difficulties were over three times more likely to not reach the KS4 GCSE benchmark (OR 3.11, 95% CI [2.11-4.57]) in the unadjusted model (Table 3). Incrementally controlling for prior attainment and

For peer review only - http://bmjopen.bmj.com/site/about/guidelines.xhtml

household socioeconomic factors did not attenuate this risk. Controlling for a young person's happiness with school and school-work (Model 5) and parental relationships and support (Model 6) partially diminished this risk. However, the fully adjusted model demonstrated that young people with poor mental health were over twice as likely (OR 2.05, 95% CI [1.15-3.68]) to not reach the educational benchmark than their counterparts with sub-clinical difficulties. Within individual sub-domains, the fully adjusted model could not account for the higher odds of not reaching the educational benchmark for those with hyperactivity disorder (OR 2.38, 95% CI [1.48-3.82]), implying that hyperactivity disorder largely drives the association between mental difficulties scores and lower attainment. For emotional and peer disorders, these risks were no longer significant once adjusted for prior attainment and sociodemographic factors, and conduct disorder no longer predicted lower attainment following adjustment for happiness with school and school-work.

Table 3: Odds ratios for low attainment at Key Stage 4 by total mental health difficulties and domain scores, adjusted stepwise for explanatory factors.

	Model 1	Model 2	Model 3	Model 4	Model 5	Model 6	Model 7
**Emotional**	1.64* [1.11,2.41]	1.88** [1.27,2.78]	1.75* [1.07,2.85]	1.55 [0.91,2.65]	1.22 [0.71,2.10]	1.12 [0.63,1.99]	1.07 [0.61,1.90]
**Peer**	2.44*** [1.66,3.58]	2.45*** [1.66,3.61]	1.67* [1.02,2.75]	1.50 [0.88,2.55]	1.31 [0.78,2.20]	1.26 [0.74,2.16]	1.20 [0.70,2.08]
**Conduct**	1.92*** [1.33,2.76]	1.83** [1.26,2.65]	1.91** [1.22,3.01]	1.65* [1.02,2.67]	1.25 [0.74,2.11]	1.10 [0.62,1.94]	1.07 [0.60,1.90]
**Hyperactivity**	2.52*** [1.80,3.52]	2.46*** [1.75,3.45]	2.77*** [1.84,4.18]	2.94*** [1.89,4.57]	2.39*** [1.52,3.78]	2.35*** [1.46,3.78]	2.38*** [1.48,3.82]
**Total score**	3.11*** [2.11,4.57]	3.25*** [2.20,4.80]	3.55*** [2.22,5.70]	3.20*** [1.90,5.37]	2.38** [1.38,4.12]	2.10* [1.17,3.77]	2.05* [1.15,3.68]

Note: Imputed model, N=1100
Model 1: unadjusted odds of low KS4 attainment
Model 2: adjusts for Model 1 + age, sex, ethnicity
Model 3: adjusts for Model 2 + prior attainment at KS2
Model 4: adjusts for Model 3 + household social class, maternal education, household poverty, family composition
Model 5: adjusts for Model 4 + happy with school work, happy with school

For peer review only - http://bmjopen.bmj.com/site/about/guidelines.xhtml

Model 6: adjusts Model 5 + parental interest in school, parents attend parent evening, family support, quarrels with parents
Model 7: adjusts for Model 6 +parental mental and physical health
*** p<0.001, **p<0.01, *p<0.05

Table 4 describes the sex-specific association between mental health difficulties and attainment to explore the well-established and significantly lower level of attainment in males than females observed in Table 1. There was an independent relationship between poor mental health and low attainment in males after controlling for all explanatory variables (OR 2.77, [1.30 to 6.29]). For females, the relationship between poor mental health and low attainment was no longer significant once prior attainment, sociodemographic factors and school enjoyment and parental support and engagement with school was controlled for.

For both sexes there were significant and generally strong associations between sub-domains of mental health and attainment. The single noteworthy exception was a lack of association with attainment in females with emotional disorder (OR 1.49, [0.91-2.43]). With exception to hyperactivity disorder, there were no significant associations with attainment in males and females after adjusting for sociodemographic factors and happiness with school. Hyperactivity disorder predicted poor academic attainment for males (OR 2.17, 95% CI 1.13 to 4.19) and females (OR 2.85, 95% CI 1.24 to 6.03) after controlling for the effects of all explanatory variables.

Table 4: Unadjusted and adjusted odds ratios for low attainment at Key Stage 4, as predicted by mental health difficulties, stratified by sex.

		Unadjusted		Fully adjusted	
Emotional	Male	3.07**	[1.48,6.38]	2.36	[0.83,6.64]
	Female	1.49	[0.91,2.43]	0.73	[0.34,1.57]
Peer	Male	2.36**	[1.39,4.02]	1.79	[0.83,3.84]
	Female	2.55**	[1.45,4.48]	0.99	[0.41,2.40]
Conduct	Male	1.65*	[1.03,2.66]	0.93	[0.42,2.05]
	Female	2.17**	[1.22,3.86]	1.29	[0.52,3.18]
Hyperactivity	Male	2.35***	[1.49,3.71]	2.17*	[1.11,4.23]
	Female	2.63***	[1.59,4.35]	2.85**	[1.30,6.23]
Total score	Male	3.16***	[1.79,5.60]	2.77*	[1.24,6.16]
	Female	3.36***	[1.97,5.71]	1.69	[0.72,3.95]

Note: Imputed model, Males N=550; Females N=560
Unadjusted: unadjusted odds of low attainment.
Fully adjusted: odds of low attainment controlling for age, ethnicity, prior attainment at KS2, household social class, maternal education, household poverty, family composition, happy with school work, happy with school, parental interest in school, parents attend parent evening, family support, quarrels with parents, parental mental and physical health. *** p<0.001, **p<0.01, *p<0.05

For peer review only - http://bmjopen.bmj.com/site/about/guidelines.xhtml

Results for the stepwise adjustment towards the full model are found in supplementary table A.

For peer review only

For peer review only - http://bmjopen.bmj.com/site/about/guidelines.xhtml

DISCUSSION

This longitudinal sample of adolescents observed a strong association between mental health difficulties between the ages of 11 and 14 and later educational attainment at age 16. After accounting for the confounding effects of a range of socioeconomic, school-based and parenting factors known to predict lower attainment, young people with mental difficulties were twice as likely to not reach the educational benchmark in England.

The association between lower attainment and overall mental difficulties was largely driven by the presence of hyperactivity disorder which remained highly significant after accounting for other explanatory factors. The relationship between hyperactivity disorder and lower attainment is has been documented elsewhere (35). Our data support the on-going development early interventions targeted towards hyperactivity disorders (36) focussing on meeting the specific needs of children and young people to enable them to reach their academic potential. Importantly, these interventions are and ought to continue to be school-based as it offers a suitable medium for universal support and equal access to provision to nearly all young people. (37)

While males and females with overall mental difficulties were equally likely to not achieve the GCSE benchmark, this relationship was only significant for males after controlling for explanatory factors. This is concurrent with previous work on the same sample assessing educational attainment at older ages, (38) which demonstrated that females at age 18 exhibited a weaker relationship between mental difficulties and attainment than males. However, in contrast to our findings at ages 11 to 14 years, females at age 18 were significantly more likely to experience poor mental health than males - females being more likely to be conscientious high achievers was suggested as a possible explanation. Although the reason for this difference needs further investigation, these findings confirm important age and sex differences which ought to be accounted for when devising interventions aimed at promoting adolescent mental health.

It is noteworthy that although family socioeconomic circumstances are well-established predictors of later performance at school (39) the association with mental health difficulties was robust to adjustment. Although the association between poorer mental health and lower attainment operated regardless of socioeconomic background, interventions to improve mental health delivered via universal and inclusive mainstream or alternative education-based settings are likely to disproportionately impact those from disadvantaged backgrounds as they are more likely to experience mental health difficulties. Based on findings presented here, improving mental health could possibly increase average attainment levels within this group to a greater extent than within the majority population who are not disadvantaged. The potential effect at a population level would be to reduce the average difference in attainment

For peer review only - http://bmjopen.bmj.com/site/about/guidelines.xhtml

between socioeconomic groups, and narrow educational and consequent social inequalities.

Overall, these data are of interest to a range for stakeholders as they offer a contemporary and contextually rich data useful for wider policymaking and practice. Furthermore, showing the strong association between social factors with attainment and mental health makes the fully adjusted independent link between mental health and attainment all the more striking highlighting that they are both important predictors of attainment.

Limitations

Consent to data linkage and successful linkage between the UKHLS and the NPD was predicted by ethnicity, household structure and social class. The inclusion of these variables in the imputation and the final models may mitigate against some of these selection effects, the lack of an analytic weight and the ethical limitation of being unable to impute missing data for sensitive information which has been actively protected by the respondent means that data may not be representative; prevalence estimates should be interpreted cautiously and may not be generalisable to the English population. This does not, however, diminish confidence in the associations identified by the prospective approach taken. Although the collection of mental difficulties data from young people is preferable than from their parents, this information was self-reported rather than a clinical diagnosis. Other measures of wellbeing and mental health ought to be considered in future analysis as associations with different constructs may differ from those presented here. Cut points for the SDQ are contested with researchers in different contexts opting for different thresholds. The SDQ developer adds the caveats to a recently devised set of cut-points that these systems “only provide a rough-and-ready way of screening for disorders”. (40) Lastly, mediation analysis has not been conducted in this study though predictors of attainment such as happiness with school may be candidate variables. Caution should be applied to interpreting these candidate mediators as current estimates of the effect of mental difficulties on attainment may be considered overadjusted.

Contributions

NS and LM designed the analysis which was carried out by LM and MA under guidance from MS and NS. NS drafted the manuscript. AH and SS contributed to the study design and drafting of the manuscript. We thank the peer-reviewers for the constructive comments on this manuscript.

Declarations of interest

None

Funding. The project was funded by the Economic and Social Research Council Secondary Data Analysis Initiative (ES/R005400/1 to NS).

For peer review only - http://bmjopen.bmj.com/site/about/guidelines.xhtml

Competing interests: None declared.

Patient consent: Obtained.

Patient and public involvement:
It was not appropriate or possible to involve patients or the public in the design, or conduct, or reporting, or dissemination plans of our research.

Provenance and peer review: Not commissioned; externally peer reviewed.

Data Sharing Statement: Data are available in a public, open access repository. All data are hosted by the UK Data Service (UKDS):

National Pupil Database data is available under secure access licence agreement to registered and approved researchers.
10.5255/UKDA-SN-7642-2

Understanding Society Main Survey Data are available to registered users under standard terms of the UKDS End User Licence Agreement.
http://doi.org/10.5255/UKDA-SN-6614-13.

Ethics Statement: The data used are publicly available via UK Data Service repository (study numbers 6614 and 8644), and do not require ethical assessment for academic research purposes. The University of Essex Ethics Committee approved the survey data collection. No ethics approval number was produced. Ethics approval for data collection was granted by letter dated 6 July 2007 for Waves 1 and 2 and by letter dated 17 December 2010 for Waves 3 to 5

https://www.understandingsociety.ac.uk/documentation/mainstage/user-guides/main-survey-user-guide/ethics

Figure 1: Flow chart describing the breakdown of the combined Wave 1 and Wave 3 study population of the UKHLS into the analytic sample

For peer review only

1. NHS Digital. Mental Health of Children and Young People in England, 2017. [Internet]. 2018. Available from: https://digital.nhs.uk/data-and-information/publications/statistical/mental-health-of-children-and-young-people-in-england/2017/2017

2. Department of Health. Future in mind - Promoting, protecting and improving our children and young people's mental health and wellbeing [Internet]. 2015 p. 81–81. Available from: https://assets.publishing.service.gov.uk/government/uploads/system/uploads/attachment_data/file/414024/Childrens_Mental_Health.pdf

3. Chowdry H, McBride T. Disadvantage, Behaviour And Cognitive Outcomes: Longitudinal Analysis From Age 5 To 16. London; 2017.

4. Crawford C, Greaves E. Socio-economic, ethnic and gender differences in HE participation. [Internet]. Department for Business, Innovation and Skills; 2015 p. 116–116. Available from: https://assets.publishing.service.gov.uk/government/uploads/system/uploads/attachment_data/file/474273/BIS-15-85-socio-economic-ethnic-and-gender-differences.pdf

5. Lessof C, Ross A, Brind R. Multiple disadvantage and KS4 attainment: evidence from LSYPE2 [Internet]. GOV.UK. 2019 [cited 2020 Apr 7]. Available from: https://www.gov.uk/government/publications/multiple-disadvantage-and-ks4-attainment-evidence-from-lsype2

6. Schoon I. Planning for the Future: Changing Education Expectations in Three British Cohorts. Historical Social Research [Internet]. 2010;35(2). Available from: http://www.jstor.org/stable/20762452?seq=1#page_scan_tab_contents

7. Flouri E. Parental interest in children's education, children's self-esteem and locus of control, and later educational attainment: twenty-six year follow-up of the 1970 British Birth Cohort. The British journal of educational psychology. 2006;76(Pt 1):41–55.

8. Sellström E, Bremberg S. Is there a "school effect" on pupil outcomes? A review of multilevel studies. J Epidemiol Community Health. 2006 Feb;60(2):149–55.

9. Hale DR, Viner RM. How adolescent health influences education and employment: investigating longitudinal associations and mechanisms. J Epidemiol Community Health. 2018 Jun 1;72(6):465–70.

10. Dalsgaard S, McGrath J, Østergaard SD, Wray NR, Pedersen CB, Mortensen PB, et al. Association of Mental Disorder in Childhood and Adolescence With Subsequent Educational Achievement. JAMA Psychiatry. 2020 Aug 1;77(8):797–805.

11. Kessler C, Saunders B, Ph D, Foster L, Stang PE, Al E. Social Consequences of Psychiatric Disorders, I: Educational Attainment. American Journal of Psychiatry. 1995;15(July):1026–32.

For peer review only - http://bmjopen.bmj.com/site/about/guidelines.xhtml

12. Veldman K, Bültmann U, Stewart RE, Ormel J, Verhulst FC, Reijneveld SA. Mental Health Problems and Educational Attainment in Adolescence: 9-Year Follow-Up of the TRAILS Study. PLOS ONE. 2014 Jul 21;9(7):e101751.

13. L Marshall RW A Dunatchik, NR Smith. Supporting Mental Health in Schools and Colleges. https://www.gov.uk/government/publications/supporting-mental-health-in-schools-and-colleges. 2017;(ISBN 978-1-78105-757-5, DFE-RR697b).

14. Rothon C, Head J, Clark C, Klineberg E, Cattell V, Stansfeld S. The impact of psychological distress on the educational achievement of adolescents at the end of compulsory education. Social Psychiatry and Psychiatric Epidemiology. 2009 May 1;44(5):421–7.

15. Deighton J, Humphrey N, Belsky J, Boehnke J, Vostanis P, Patalay P. Longitudinal pathways between mental health difficulties and academic performance during middle childhood and early adolescence. The British journal of developmental psychology. 2018 Mar 1;36(1):110–26.

16. Clayborne ZM, Varin M, Colman I. Systematic Review and Meta-Analysis: Adolescent Depression and Long-Term Psychosocial Outcomes. J Am Acad Child Adolesc Psychiatry. 2019 Jan;58(1):72–9.

17. Sellers R, Warne N, Pickles A, Maughan B, Thapar A, Collishaw S. Cross-cohort change in adolescent outcomes for children with mental health problems. Journal of Child Psychology and Psychiatry. 2019 Jul 31;60(7):813–21.

18. Marshall L, Wishart R, Dunatchik A, Smith N. Supporting Mental Health in Schools and Colleges [Internet]. 2017 [cited 2020 Jul 4]. Available from: https://assets.publishing.service.gov.uk/government/uploads/system/uploads/attachment_data/file/634726/Supporting_Mental-Health_survey_report.pdf

19. University of Essex, Institute for Social and Economic Research, NatCen Social Research, Kantar Public. Understanding Society: Waves 1-9, 2009-2018 and Harmonised BHPS: Waves 1-18, 1991-2009. [data collection]. 12th Edition. UK Data Service. 2019. 2019.

20. Institute for Social and Economic Research. Understanding Society: The UK Household Longitudinal Study, Waves 1-9 - User Guide [Internet]. University of Essex; 2019 [cited 2020 Sep 4]. Available from: https://www.understandingsociety.ac.uk/sites/default/files/downloads/documentation/mainstage/user-guides/mainstage-user-guide.pdf

21. Jay MA, Grath-Lone LM, Gilbert R. Data Resource: the National Pupil Database (NPD). International Journal of Population Data Science [Internet]. 2019 Mar 20 [cited 2021 Jun 7];4(1). Available from: https://ijpds.org/article/view/1101

22. Knies G, Burton J. Analysis of four studies in a comparative framework reveals: health linkage consent rates on British cohort studies higher than

For peer review only - http://bmjopen.bmj.com/site/about/guidelines.xhtml

on UK household panel surveys. BMC medical research methodology. 2014 Nov;14:125–125.

23. Department for Education. GCSE and equivalent results: 2011 to 2012 (revised) [Internet]. 2013 Jan [cited 2021 Jun 7]. Available from: https://www.gov.uk/government/statistics/revised-gcse-and-equivalent-results-in-england-academic-year-2011-to-2012

24. Goodman R. The Strengths and Difficulties Questionnaire: a research note. Journal of Child Psychology and Psychiatry. 1997;38(5):581–6.

25. Biederman J, Monuteaux MC, Doyle AE, Seidman LJ, Wilens TE, Ferrero F, et al. Impact of Executive Function Deficits and Attention-Deficit/Hyperactivity Disorder (ADHD) on Academic Outcomes in Children. Journal of Consulting and Clinical Psychology. 2004 Oct;72(5):757–66.

26. Woodward LJ, Fergusson DM. Childhood peer relationship problems and later risks of educational under-achievement and unemployment. Journal of child psychology and psychiatry, and allied disciplines. 2000 Feb;41(2):191–201.

27. Deighton J, Lereya ST, Casey P, Patalay P, Humphrey N, Wolpert M. Prevalence of mental health problems in schools: poverty and other risk factors among 28 000 adolescents in England. British Journal of Psychiatry. 2019;215(3):565–7.

28. Strand S. The limits of social class in explaining ethnic gaps in educational attainment. British Educational Research Journal. 2011;37(2):197–229.

29. Goodman A, Gregg P. Poorer children’s educational attainment: how important are attitudes and behaviour? [Internet]. JRF. 2010 [cited 2021 Jun 7]. Available from: https://www.jrf.org.uk/report/poorer-children%E2%80%99s-educational-attainment-how-important-are-attitudes-and-behaviour

30. Carneiro P, Meghir C, Parey M. Maternal Education, Home Environments, and the Development of Children and Adolescents. Journal of the European Economic Association. 2013 Jan 1;11(suppl_1):123–60.

31. Rose D, Pevalin DJ. The National Statistics Socio-economic Classification (NS-SEC rebased on the SOC2010). Palgrave Macmillan; 2011.

32. Knies G. Income effects on children’s life satisfaction: Longitudinal evidence for England [Internet]. ISER Working Paper Series; 2017 [cited 2021 Jun 7]. Report No.: 2017–02. Available from: https://www.econstor.eu/handle/10419/163545

33. Ware Jr JE, Kosinski M, Keller SD. A 12-Item Short-Form Health Survey: construction of scales and preliminary tests of reliability and validity. Medical care. 1996;34(3):220–33.

For peer review only - http://bmjopen.bmj.com/site/about/guidelines.xhtml

34. Suldo SM, Riley KN, Shaffer EJ. Academic Correlates of Children and Adolescents' Life Satisfaction. School Psychology International. 2006 Dec 1;27(5):567–82.

35. Birchwood J, Daley D. Brief report: The impact of Attention Deficit Hyperactivity Disorder (ADHD) symptoms on academic performance in an adolescent community sample. Journal of Adolescence. 2012 Feb 1;35(1):225–31.

36. May F, Ford T, Janssens A, Newlove-Delgado T, Russell AE, Salim J, et al. Attainment, attendance, and school difficulties in UK primary schoolchildren with probable ADHD. British Journal of Educational Psychology. 2021;91(1):442–62.

37. Department for Education, Department of Health and Social Care. Transforming children and young people's mental health provision: a green paper [Internet]. GOV.UK. 2018 [cited 2021 Jun 7]. Available from: https://www.gov.uk/government/consultations/transforming-children-and-young-peoples-mental-health-provision-a-green-paper

38. Tregaskis O, Nandi A, Watson D. An examination of the relationship between adolescent mental health and educational outcomes in early adulthood: Secondary analysis of Understanding Society data wave 1 to wave 8. 2020.

39. Benton T, Such, T. Analysis of use of Key Stage 2 data in GCSE predictions [Internet]. OFQUAL; 2014 [cited 2021 Jun 7]. Available from: https://assets.publishing.service.gov.uk/government/uploads/system/uploads/attachment_data/file/429074/2014-06-16-analysis-of-use-of-key-stage-2-data-in-gcse-predictions.pdf

40. Goodman R. Scoring the Strengths & Difficulties Questionnaire for age 4-17 or 18+ [Internet]. Youth in Mind. [cited 2021 Jun 7]. Available from: https://www.sdqinfo.org/py/sdqinfo/b3.py?language=Englishqz(UK)

For peer review only

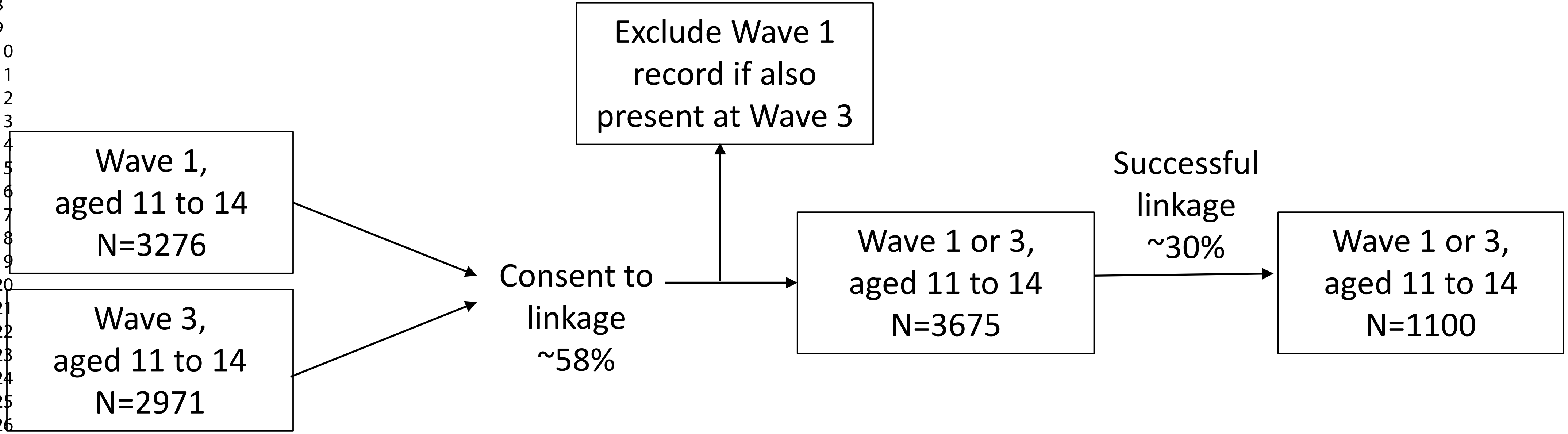
Exclude Wave 1 record if also present at Wave 3
Wave 1, aged 11 to 14 N=3276
Wave 3, aged 11 to 14 N=2971
Consent to linkage ~58%
Wave 1 or 3, aged 11 to 14 N=3675
Successful linkage ~30%
Wave 1 or 3, aged 11 to 14 N=1100

Supplementary table A: Odds ratios for low attainment at Key Stage 4 by total mental health difficulties and domain scores, adjusted stepwise for explanatory factors, by sex.

	Emotional		Peer		Conduct		Hyperactivity		Total score	
	Males	Females	Males	Females	Males	Females	Males	Females	Males	Females
Model 1	**3.07** [1.48,6.38]**	1.49 [0.91,2.43]	**2.36** [1.39,4.02]**	**2.55** [1.45,4.48]**	**1.65* [1.03,2.66]**	**2.17** [1.22,3.86]**	**2.35*** [1.49,3.71]**	**2.63*** [1.59,4.35]**	**3.16*** [1.79,5.60]**	**3.36*** [1.97,5.71]**
Model 2	**3.22** [1.55,6.71]**	1.54 [0.95,2.50]	**2.43** [1.43,4.14]**	**2.78*** [1.59,4.86]**	**1.71* [1.06,2.76]**	**2.15* [1.18,3.89]**	**2.40*** [1.51,3.79]**	**2.61*** [1.56,4.37]**	**3.30*** [1.85,5.87]**	**3.47*** [2.03,5.92]**
Model 3	**2.91* [1.17,7.22]**	1.41 [0.77,2.57]	**2.34* [1.20,4.56]**	1.31 [0.61,2.79]	1.56 [0.86,2.85]	**2.67** [1.35,5.26]**	**2.40** [1.36,4.24]**	**3.45*** [1.90,6.27]**	**3.39*** [1.74,6.62]**	**3.91*** [2.04,7.51]**
Model 4	**2.89* [1.05,7.92]**	1.23 [0.63,2.42]	**2.23* [1.07,4.63]**	1.26 [0.55,2.90]	1.44 [0.76,2.72]	**2.42* [1.16,5.05]**	**2.68** [1.43,5.04]**	**3.43*** [1.75,6.73]**	**3.38** [1.64,6.98]**	**3.52*** [1.69,7.32]**
Model 5	2.37 [0.85,6.59]	0.98 [0.49,1.97]	1.90 [0.93,3.87]	1.12 [0.48,2.60]	1.03 [0.50,2.12]	1.82 [0.85,3.93]	**2.23* [1.15,4.31]**	**2.64** [1.35,5.18]**	**2.66* [1.25,5.70]**	**2.61* [1.22,5.57]**
Model 6	2.51 [0.87,7.28]	0.76 [0.35,1.65]	1.85 [0.88,3.90]	1.09 [0.45,2.64]	0.98 [0.45,2.14]	1.35 [0.54,3.32]	**2.17* [1.13,4.19]**	**2.73* [1.24,6.03]**	**2.86** [1.30,6.29]**	1.79 [0.76,4.25]
Model 7	2.36 [0.83,6.64]	0.73 [0.34,1.57]	1.79 [0.83,3.84]	0.99 [0.41,2.40]	0.93 [0.42,2.05]	1.29 [0.52,3.18]	**2.17* [1.11,4.23]**	**2.85** [1.30,6.23]**	**2.77* [1.24,6.16]**	1.69 [0.72,3.95]

Note: Imputed model, Males N=550; Females N=560

Model 1: unadjusted odds of socioemotional difficulties; Model 2: adjusts for Model 1 + age, ethnicity; Model 3: adjusts for Model 2 + prior attainment at KS2; Model 4: adjusts for Model 3 + household social class, maternal education, household poverty, family composition Model 5: adjusts for Model 4 + happy with school work, happy with school; Model 6: adjusts Model 5 + parental interest in school, parents attend parent evening, family support, quarrels with parents; Model 7: adjusts for Model 6 +parental mental and physical health. Significant odds ratios (95% confidence interval) shown in bold text.

For peer review only

For peer review only

STROBE 2007 (v4) Statement—Checklist of items that should be included in reports of *cohort studies*

Section/Topic	Item #	Recommendation	Reported on page #
**Title and abstract**	1	(*a*) Indicate the study's design with a commonly used term in the title or the abstract	0
		(*b*) Provide in the abstract an informative and balanced summary of what was done and what was found	1
**Introduction**			
Background/rationale	2	Explain the scientific background and rationale for the investigation being reported	3
Objectives	3	State specific objectives, including any prespecified hypotheses	4
**Methods**			
Study design	4	Present key elements of study design early in the paper	4, 5
Setting	5	Describe the setting, locations, and relevant dates, including periods of recruitment, exposure, follow-up, and data collection	5
Participants	6	(*a*) Give the eligibility criteria, and the sources and methods of selection of participants. Describe methods of follow-up	5
		(*b*) For matched studies, give matching criteria and number of exposed and unexposed	n/a
Variables	7	Clearly define all outcomes, exposures, predictors, potential confounders, and effect modifiers. Give diagnostic criteria, if applicable	5, 6
Data sources/ measurement	8*	For each variable of interest, give sources of data and details of methods of assessment (measurement). Describe comparability of assessment methods if there is more than one group	5, 6
Bias	9	Describe any efforts to address potential sources of bias	6, 15
Study size	10	Explain how the study size was arrived at	6
Quantitative variables	11	Explain how quantitative variables were handled in the analyses. If applicable, describe which groupings were chosen and why	5, 6
Statistical methods	12	(*a*) Describe all statistical methods, including those used to control for confounding	6, 7
		(*b*) Describe any methods used to examine subgroups and interactions	7
		(*c*) Explain how missing data were addressed	7
		(*d*) If applicable, explain how loss to follow-up was addressed	5, 7
		(*e*) Describe any sensitivity analyses	n/a
**Results**			

For peer review only

Participants	13*	(a) Report numbers of individuals at each stage of study—eg numbers potentially eligible, examined for eligibility, confirmed eligible, included in the study, completing follow-up, and analysed	5, 8, 9. 10, 11
		(b) Give reasons for non-participation at each stage	5
		(c) Consider use of a flow diagram	5
Descriptive data	14*	(a) Give characteristics of study participants (eg demographic, clinical, social) and information on exposures and potential confounders	8-11
		(b) Indicate number of participants with missing data for each variable of interest	8-11
		(c) Summarise follow-up time (eg, average and total amount)	n/a
Outcome data	15*	Report numbers of outcome events or summary measures over time	8-11
Main results	16	(*a*) Give unadjusted estimates and, if applicable, confounder-adjusted estimates and their precision (eg, 95% confidence interval). Make clear which confounders were adjusted for and why they were included	12, 13
		(*b*) Report category boundaries when continuous variables were categorized	5, 6
		(*c*) If relevant, consider translating estimates of relative risk into absolute risk for a meaningful time period	n/a
Other analyses	17	Report other analyses done—eg analyses of subgroups and interactions, and sensitivity analyses	13
**Discussion**			
Key results	18	Summarise key results with reference to study objectives	14
**Limitations**			
Interpretation	20	Give a cautious overall interpretation of results considering objectives, limitations, multiplicity of analyses, results from similar studies, and other relevant evidence	14,15
Generalisability	21	Discuss the generalisability (external validity) of the study results	15
**Other information**			
Funding	22	Give the source of funding and the role of the funders for the present study and, if applicable, for the original study on which the present article is based	15,16

*Give information separately for cases and controls in case-control studies and, if applicable, for exposed and unexposed groups in cohort and cross-sectional studies.

Note: An Explanation and Elaboration article discusses each checklist item and gives methodological background and published examples of transparent reporting. The STROBE checklist is best used in conjunction with this article (freely available on the Web sites of PLoS Medicine at http://www.plosmedicine.org/, Annals of Internal Medicine at http://www.annals.org/, and Epidemiology at http://www.epidem.com/). Information on the STROBE Initiative is available at www.strobe-statement.org.

For peer review only
